# A Meta-Analysis on Antecedents and Outcomes of Detachment from Work

**DOI:** 10.3389/fpsyg.2016.02072

**Published:** 2017-01-13

**Authors:** Johannes Wendsche, Andrea Lohmann-Haislah

**Affiliations:** ^1^Federal Institute for Occupational Safety and Health, Regional Transfer/Special SectorsDresden, Germany; ^2^Federal Institute for Occupational Safety and Health, Mental WorkloadBerlin, Germany

**Keywords:** detachment, meta-analysis, recovery, rumination, stress, work reflection

## Abstract

Detachment from work has been proposed as an important non-work experience helping employees to recover from work demands. This meta-analysis (86 publications, *k* = 91 independent study samples, *N* = 38,124 employees) examined core antecedents and outcomes of detachment in employee samples. With regard to outcomes, results indicated average positive correlations between detachment and self-reported mental (i.e., less exhaustion, higher life satisfaction, more well-being, better sleep) and physical (i.e., lower physical discomfort) health, state well-being (i.e., less fatigue, higher positive affect, more intensive state of recovery), and task performance (small to medium sized effects). However, average relationships between detachment and physiological stress indicators and work motivation were not significant while associations with contextual performance and creativity were significant, but negative. Concerning work characteristics, as expected, job demands were negatively related and job resources were positively related to detachment (small sized effects). Further, analyses revealed that person characteristics such as negative affectivity/neuroticism (small sized effect) and heavy work investment (medium sized effect) were negatively related to detachment whereas detachment and demographic variables (i.e., age and gender) were not related. Moreover, we found a medium sized average negative relationship between engagement in work-related activities during non-work time and detachment. For most of the examined relationships heterogeneity of effect sizes was moderate to high. We identified study design, samples' gender distribution, and affective valence of work-related thoughts as moderators for some of these aforementioned relationships. The results of this meta-analysis point to detachment as a non-work (recovery) experience that is influenced by work-related and personal characteristics which in turn is relevant for a range of employee outcomes.

## Introduction

Early research on employee recovery from work indicates that stressful work characteristics can negatively affect physiological unwinding after work (Frankenhaeuser, 1981). However, it was the study of Etzion et al. (1998) that turned scholars interest to the specific role of *psychological* after-work recovery processes by introducing the concept of *detachment from work* (Sonnentag and Fritz, 2007). In a recent narrative review, Sonnentag and Fritz (2015) summarized relationships between work stressors, detachment, and employee well-being in the so-called “Stressor-Detachment Model” (SDM). Building on the SDM, our meta-analysis will examine antecedents and outcomes of detachment. While Sonnentag and Fritz (2015) focus on job stressors as antecedents and strain and well-being as outcomes, our meta-analysis will expand their work in several ways. First, we go beyond the narrative approach of Sonnentag and Fritz (2015) by *statistically synthesizing* previous empirical data which allows a more precise interpretation of relationships between variables (Borenstein et al., 2009). Second, in addition to examining job demands and stressors as work-related antecedents of detachment we also include *job-related resources* which have been proposed as further antecedent (Kinnunen et al., 2011). Third, in our study, we also investigate the associations between detachment and person characteristics such as *demographic* factors (i.e., age, gender) and *psychological individual differences* (i.e., negative affectivity/neuroticism, heavy work investment). Moreover, fourth, we will explore the relationship between employees' work-related activities during non-work time and detachment. Fifth, in additional to well-being and strain, we also examine work motivation and job performance as outcomes of detachment. Finally, we statistically examine the potential moderating role of various study-related, individual, and conceptual variables for the functional relationships of detachment from work.

### Detachment concept

Etzion et al. (1998) introduced the term detachment to describe “the individual's sense of being away from the work situation” (p. 579). Sonnentag and Bayer (2005) describe psychological detachment as a state of distancing oneself mentally from job-related thoughts during non-work time. Thus, by definition employees are no longer occupied with work-related tasks neither in a physical nor in a mental way. Moreover, psychological detachment is an important recovery experience (Sonnentag and Fritz, 2007). Recovery is a process in which psychophysiological systems that were activated due to effort expenditure return to their baseline levels after work demands are removed (Meijman and Mulder, 1998). As a result, recovery from work can improve employees' mental and physical well-being, as it reduces physiological activation and increases work motivation as well as job performance by replenishing individuals' mental and physiological resources (Sonnentag and Geurts, 2009; Zijlstra et al., 2014). Below we discuss constructs similar to psychological detachment to create a broader conceptualization for this meta-analysis: detachment from work.

#### Dimension

In contrast to psychological detachment, which focusses on mental disengagement from or absence of work-related thoughts, other concepts like work rumination (Cropley and Zijlstra, 2011) or work reflection (Fritz and Sonnentag, 2005) point to the presence of work-related thoughts during non-work time. In our review, we regard them as opposite ends of one dimension of mental distancing from work during off-job time.

#### Context of detachment

Detachment is a recovery process that occurs during non-work time (Sonnentag and Fritz, 2007). Geurts and Sonnentag (2006) contrast internal recovery (i.e., within working time) and external recovery (i.e., after finishing daily work). Thus, detachment can occur during work breaks (Coffeng et al., 2015), in the afternoon (Sonnentag and Bayer, 2005), in the evening (Sonnentag and Fritz, 2007), at the weekend (Fritz et al., 2010a), or in a longer recovery period like vacation (de Bloom et al., 2013). In our study, we only focus on detachment from work during *daily* non-work time (afternoon, evening) as it is most frequently studied in this research domain (Sonnentag and Fritz, 2015).

#### Valence of work-related thoughts

According to Geurts (2014), recovery is characterized by three interrelated processes: behavioral reactions (no exposure to work demands) and cognitive processes (no work-related thoughts)—both are included in the definition of psychological detachment (Sonnentag and Fritz, 2015). The third type of process—affective reactions—refers to a decrease in negative and an increase in positive affect. Sonnentag and Fritz (2007, 2015) consider psychological detachment as an affectively neutral concept (Sonnentag and Fritz, 2015). However, if one cannot detach from work, the affective valence of work-related thoughts might determine the outcomes of recovery. For example, some authors propose that thinking about positive work events (positive work reflection: Binnewies et al., 2009; positive rumination: Frone, 2015) increases meaningfulness of work and self-efficacy, and helps to reevaluate work stressors and to develop new goals and plans. Thus, positively thinking about work during non-work time may even improve well-being, and performance capacity. Some research has pointed to negative affective processes (negative rumination: Frone, 2015; affective rumination: Cropley and Zijlstra, 2011; negative work reflection: Binnewies et al., 2009). More specifically, negative thoughts about work during non-work time might hamper recovery as they foster prolonging physiological activation and may reduce self-efficacy, control, and attentional capacity (Binnewies et al., 2009). Thus, we follow Sonnentag and Fritz's (2015) recommendation to examine the potential moderating role of valence of work-related thoughts during non-work time.

#### Recovery process vs. cognitive irritation

While Sonnentag and colleagues (Sonnentag and Bayer, 2005; Sonnentag and Fritz, 2007, 2015) define detachment as a recovery process, Mohr et al. (2005) discuss problems in switching-off mentally from work as a mid-to-long-term strain reaction (cognitive irritation). In our research, we examine detachment as a daily recovery process that antecedes strain outcomes (i.e., health, well-being, motivation, and performance). Thus, we will not include studies that examine cognitive irritation according to the concept of Mohr et al. (2005).

### Outcomes of detachment

Recovery from work can affect psychological, physiological, and behavioral outcomes (Sonnentag and Geurts, 2009). For example—in the case of detachment—if an employee cannot detach from work in the evening, sleep might be impaired which could translate into adverse psychological (e.g., feeling fatigued), physiological (e.g., lower nocturnal recovery of blood pressure), and behavioral (e.g., shorter sleeping time) reactions. At work the next morning, symptoms of fatigue and exhaustion might linger and, consequently, attention deficits occur which could result in more errors or lower work speed. In such situations, employees might also use adaptive strategies such as more effort investment to prevent a decline in work performance (Hockey, 1997). However, psychological and physiological costs of this strategy accumulate with time and at a certain point, work performance will decrease and more severe health problems will occur.

Considering the multi-symptomatic consequences of detachment from work, our meta-analysis will investigate the following outcome variables: *mental* (e.g., exhaustion, general well-being, life satisfaction) and *physical* (e.g., physical discomfort, dysregulation of physiological parameters) *health, state well-being* (e.g., fatigue, positive affect), *work motivation* (e.g., job engagement, intrinsic motivation), and *job performance* (e.g., task performance, contextual performance).

The Effort-Recovery Model (ERM; Meijman and Mulder, 1998) is helpful in explaining how detachment affects those four groups of variables. At work employees mobilize physical and psychological resources for goal-attainment (Quinn et al., 2012). Throughout the workday, these (limited) resources are depleted (Quinn et al., 2012) and adverse immediate load reactions develop with time-on-task. However, such consequences are reversible if resources are replenished during times in which demands are removed. The ERM further proposes, that if recovery is impaired repetitively, negative short-term load reactions will accumulate to adverse long-term consequences over time, especially when strategies like compensatory effort investment additionally decrease the remaining resources. For instance, Brosschot et al. (2005, 2006) argued that perseverative cognition about stressors prolong stress experiences and physiological stress reactions which was confirmed in a recent meta-analysis (Ottaviani et al., 2016). Thus, detachment from work is a useful recovery strategy to stop this chain of processes, because it separates employees mentally from further work demands and, thus, facilitates resource replenishment.

Some scholars have also noted that effects of detachment on these outcomes might be translated indirectly by other variables. For instance, high detachment was found to be positively related to a healthier life style (eating behavior: Cropley et al., 2012; alcohol use: Frone, 2015). In addition, detachment is positively related to non-work experiences like relaxation, mastery, or control (Sonnentag and Fritz, 2007) that further improve recovery from strain.

Based on this literature, we expect that detachment from work will be positively related to health, state well-being, work motivation, and job performance.

### Antecedents of detachment

Within the meta-analysis, we consider two broad groups of antecedents that have gained scholarly interest in research on detachment from work: *work-characteristics* and *person characteristics*. Moreover, we will focus on the role of *work-related activities during non-work time* covering both categories.

#### Work characteristics

The SDM (Sonnentag, 2011; Sonnentag and Fritz, 2015) assumes job stressors as core negative antecedents of psychological detachment. Job stressors are factors in the work environment that lead to strain reactions. More broadly, Bakker and Demerouti (2007) define *job demands* as work characteristics that require sustained physical and/or psychological effort and, thus, are associated with physiological and/or psychological costs. Job demands can turn into job stressors if recovery from load reactions is impaired (Meijman and Mulder, 1998). In contrast, physical, psychological, or organizational factors that promote goal achievement, reduce job demands or their strain-related consequences, and stimulate personal growth, learning, and development are called *job resources* (Bakker and Demerouti, 2007). Kinnunen et al. (2011) proposed a *Job Demands-Resources-Recovery-Model* in which job demands are negatively and job resources are positively related to recovery (e.g., detachment) from work. Cognitive and affective processes may link work characteristics and detachment from work. Smit (2016) argues that low detachment might result from unfulfilled work goals that remain active and accessible even after work. On the one hand, high job demands might induce severe goal-discrepancies, which then result in problems cognitively disengaging from work (Martin and Tesser, 1996). On the other hand, by definition, job resources promote goal attainment (Bakker and Demerouti, 2007) and, therefore, should be positively related to detachment from work. Referring to affective processes, an experimental study Radstaak et al. (2011) showed that participants reported more severe problems to detach from a stressful work condition after inducing negative emotions than after an affectively neutral condition. In a more naturalistic work setting, Bono et al. (2013) showed that negative work events were related to lower detachment from work in the evening whereas positive work events were related to higher detachment. A recent study by Ohly and Schmitt (2015) revealed that positive and negative work events might arise from specific work characteristics such as higher job resources in the first case and higher job demands in the second case. Thus, specific affective work events and coupled positive or negative emotions might drive the relationship between work characteristics and detachment from work. We expect that detachment from work will be negatively related to job demands and positively related to job resources.

#### Work-related activities during non-work time

New information and communication technology (e.g., internet, smartphones, and laptops) makes work more flexible but also leads to increased employee availability during non-work time (Duranova and Ohly, 2016). The ERM (Meijman and Mulder, 1998) predicts that work-related activities during non-work time will increase strain reactions because they further deplete resources and shorten time for resource replenishment. Moreover, employees who engage in work-related activities during non-work time will report lower detachment from work as the physical and mental presence of work increases. Therefore, we expect that engagement in work-related activities during non-work time will be negatively associated with detachment from work.

#### Person characteristics

Research has pointed to the potential role of person characteristics in recovery processes (Geurts and Sonnentag, 2006; Sonnentag and Fritz, 2007, 2015). Such factors might be *attitudinal* in nature or more *biologically* determined. In our review, we focus on two important groups of attitudinal factors. First, among the well-known big five factors, emotional instability showed the highest negative correlation to detachment (Sonnentag and Fritz, 2007). *Neuroticism and negative affectivity* have been both defined as individuals' stable tendency to be more sensitive to experience negative emotions across time and situations (Watson et al., 1988; Costa and McCrae, 1992). We expect persons high in neuroticism and negative affectivity to report lower detachment as they experience work demands more intensively (Bowling et al., 2015). Second, some scholars discuss effects of work-related personality constructs that are all related to a general tendency of *heavy work investment* (Snir and Harpaz, 2012; workaholism: Oates, 1971; overcommitment: Siegrist et al., 2004; obsessive work passion: Vallerand and Houlfort, 2003). People high in heavy work investment spend more time and invest more energy in work-related activities. These behaviors reflect an inappropriate dependence on work activities. Thus, people high in heavy work investment should have more problems in becoming distracted mentally from work during non-work time and should report lower detachment from work.

For biological or demographic variables like age and gender there is to our knowledge no reason on the basis of existing literature to assume direct relationships with detachment from work. Therefore, we examine the relationships between detachment and both variables in an exploratory manner.

#### Moderator variables

Detachment from work has been studied in various contexts, with different study designs, and with different samples over the years (Sonnentag and Fritz, 2015). Therefore, we examine possible moderating effects of *study location, study design*, and *demographic sample characteristics* (mean age, percentage of females) on all analyzed relationships. As mentioned above, Sonnentag and Fritz (2015) point to the *valence of work-related thoughts* during non-work time as potentially important moderating variable for the relationship between detachment and employee outcomes. Low detachment from work may not always be detrimental to employee outcomes (Cropley and Zijlstra, 2011). Based on these assumptions, we expect that recovery will be impaired the most if employees cannot detach from negative work-related thoughts during non-work time. Thus, we expect the strongest correlation between affectively negative measures of detachment and employee outcomes. In contrast, thinking about positive aspects of work during non-work time might even have positive effects on employee outcomes. Thus, we expect weaker correlations between detachment from work and employee outcomes here. For studies that measure detachment as affective neutral concept, we expect correlations that lie in-between.

### Research framework

**Figure 1:**
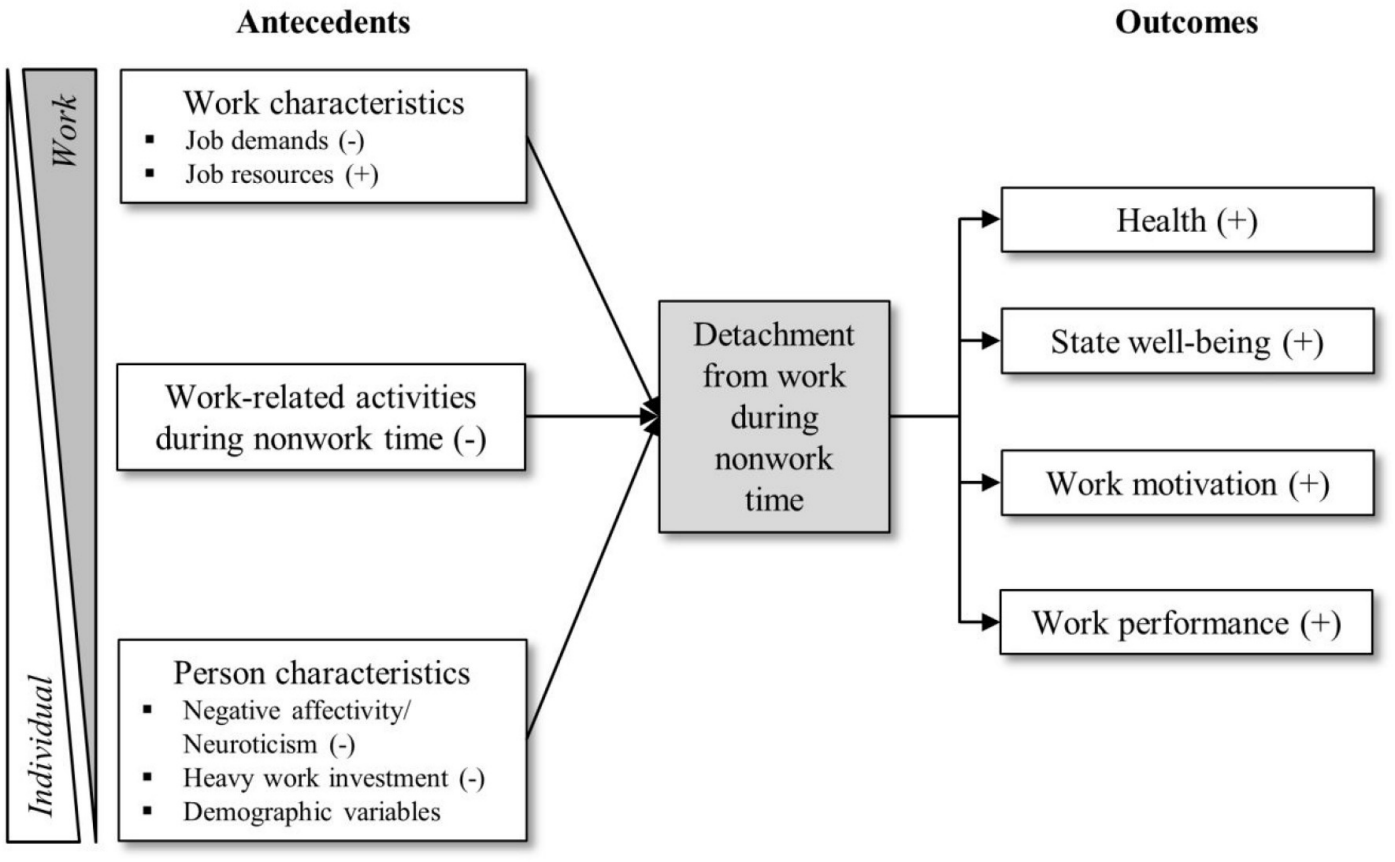
**Conceptual research model for relationships between detachment from work during non-work time and selected antecedents and outcomes (direction of assumed relationships in brackets)**.

We have summarized our general conceptual research model in Figure 1.

## Methods

### Literature search and study inclusion

Studies had to meet the following criteria to be included in our meta-analysis: (a) quantitative data reported, (b) employee sample, (c) assessment of absence (e.g., detachment) or presence (e.g., rumination, work reflection) of work-related thoughts during non-work time after a work shift (i.e., studies measuring work-related thoughts during time-intervals longer than 1 day or during work were excluded), (d) statistical association (*r*s, regression coefficients) reported between detachment from work and variables measuring work characteristics, or physical and mental health, or state well-being, or work motivation, or work performance, or person characteristics (age, gender, negative affectivity, neuroticism, heavy work investment), or time for daily work-related activities during non-work time, (e) publication in a scientific journal, and (f) article written in English.

**Figure 2:**
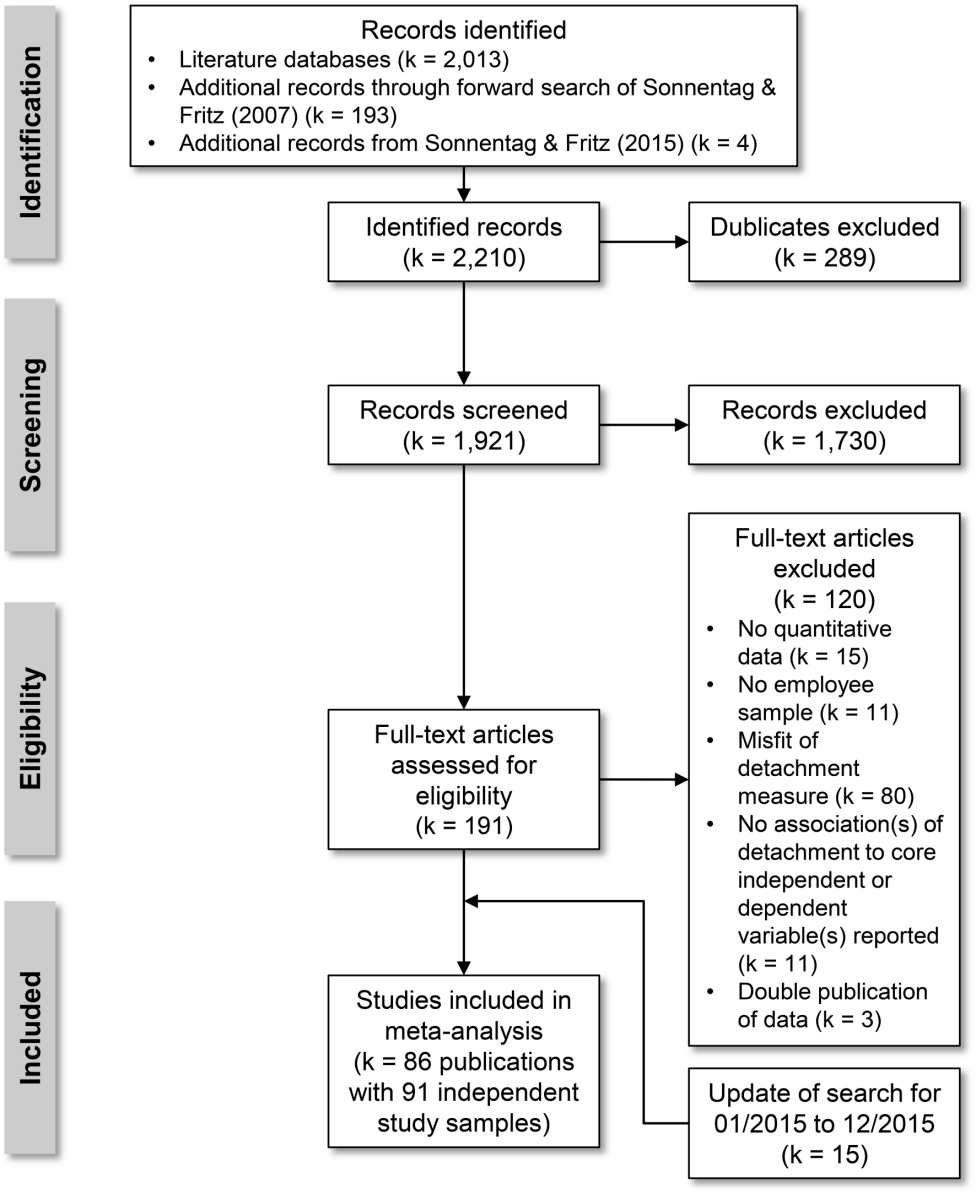
**PRSIMA study flow diagram for identification of studies to include in the meta-analysis**.

We conducted a systematic and stepwise literature search according to the PRISMA statement (Moher et al., 2009) to identify such studies. The PRISMA study flow diagram is shown in Figure 2.

#### Identification of studies

First, we screened several literature databases (PsycINFO, PsycARTICLES, and PubMed) with the following search string: (detachment OR switch-off OR off-job engagement OR ruminat^*^ OR work reflection) AND (work^*^ OR occupation^*^ OR job^*^ OR employ^*^) for the years 1998 (first publication on detachment from work, Etzion et al., 1998) to December 2014. Second, we used a forward search for the first publication of the major measurement instrument in this type of research (Recovery Experience Questionnaire; Sonnentag and Fritz, 2007). Third, we checked the references of the last systematic and narrative review on psychological detachment (Sonnentag and Fritz, 2015) for additional publications. This initial search yielded 2210 studies. After removing duplicates 1921 potential studies remained.

#### Screening

We excluded 1730 studies after screening titles and abstracts (no thematic fit: *k* = 1722, no English publication: *k* = 6, no scientific journal publication: *k* = 2).

#### Eligibility

We checked *k* = 191 full-text articles for inclusion. We excluded *k* = 120 studies for the following reasons: (a) no quantitative study (*k* = 15), (b) no employee sample (*k* = 11), (c) measure of detachment from work was inappropriate (trait measures, no work-related detachment, detachment from work during rest breaks or non-work periods longer than 1 day, *k* = 80), (d) no associations to our core independent and dependent variables reported (*k* = 11), (e) double publication of data (*k* = 3).

#### Inclusion

The sample for inclusion consisted of 71 publications with *k* = 75 independent study samples. After this original search and a first run of analyses, we updated our literature search for the period 01/2015 to 12/2015 according to the scheme described above. This yielded 15 further publications for study inclusion. The final sample in our meta-analysis consisted of data from 86 publications with *k* = 91 independent study samples (*N* = 38,124 employees).

### Study coding

#### Detachment from work

Detachment was operationalized as absence of work-related thoughts during daily non-work time. However, we also coded studies using inverse measures, thus, asking for the presences of work-related thoughts during non-work time (e.g., rumination, work reflection, problem solving pondering).

#### Outcomes of detachment

We examined the following employee outcome variables: (a) physical and mental health, (b) state well-being, (c) work motivation, and (d) work performance. *Health-related variables* were physiological stress indicators (e.g., systolic and diastolic blood pressure, cortisol, heart rate variability), self-reported physical discomfort (e.g., negative physical symptoms and physical complaints), sleep (e.g., sleep quantity, sleep quality, sleep inconsistency, sleeping time), well-being (e.g., general well-being, psychological health, trait positive affect, mental complaints, anxiety, distress, depressive symptoms, trait negative affect), burnout (exhaustion, other dimensions as cynicism), and life satisfaction. As *state well-being* we coded measures as fatigue (e.g., fatigue, need for recovery), affect (e.g., state positive and negative affect; all coded in a positive affective direction), and self-reported state of recovery. *Work motivation* was represented by measures such as work engagement, intrinsic work motivation, and commitment. We coded three aspects of *work performance*: task performance (e.g., task or job or work performance, service rule commitment), contextual performance (e.g., personal initiative, organizational citizenship behavior), and creativity.

#### Antecedents of detachment

##### Work characteristics

We coded correlations to two types of work characteristics according to the Job Demands-Resources Model (Bakker and Demerouti, 2007): job demands and job resources. We used the definitions of Bakker and Demerouti (2007) as well as the checklist of Schaufeli and Taris (2014) for categorization of measures in the primary studies. *Job demands* included measures of quantitative work demands (e.g., time pressure), social conflicts (e.g., bullying), role stressors (e.g., role conflicts), emotional demands, working hours, and others (e.g., shift work, situational constraints, cognitive demands, insecurity, self-control demands, illegitimate tasks, and physical demands). *Job resources* included measures as job control (e.g., autonomy, control), social support (e.g., supervisor support, support from colleagues), and others (e.g., organizational justice, positive affective events, learning and developmental opportunities, cognitive resources, emotional resources, physical resources, task variety).

##### Work-related activities during non-work time

We assessed correlations to two measures of engagement in work-related activities during non-work time. First, self-reports about time employees use for work-related activities during non-work time and second, self-reports about media or technology use for work-related activities during non-work time (e.g., work-related smartphone use during non-work time, work-related technology use).

##### Person characteristics

As person characteristics, we investigated demographic variables and psychological traits. We coded age and gender (positive correlations for females) as *demographic variables*. As psychological traits, we coded measures of *negative affectivity/neuroticism* (e.g., negative affectivity, neuroticism, emotional instability) and *heavy work* investment (i.e., workaholism, overcommitment, work addiction, working compulsory).

#### Potential moderator variables

We assessed *study-related variables* as study location (Europe, USA/Canada, others) and study design (cross-sectional with one measurement occasion, diary study, and longitudinal) for each sample. Moreover, we recorded *demographic study differences* as the percentage of females and employees' mean age. In addition, as highlighted above, we examined the moderating role of *affective valence* concerning detachment. Thus, we coded the valence of work-related thoughts in these measures as neutral (e.g., detachment), negative (e.g., rumination, negative work rumination, negative work reflection, affective rumination), and positive (e.g., positive work reflection, positive work rumination, problem solving pondering).

### Meta-analytic strategy

For each study, we assessed between-person correlation coefficients as measures of effect sizes. In one case (Coffeng et al., 2015) we used Comprehensive Meta-Analysis software 2.2 (Biostat, Inc, Englewood, NJ) to convert the reported unstandardized beta-weight into a correlation coefficient. We standardized the direction of correlation coefficients between studies to produce consistent meanings of effect sizes. For instance, associations of variables to inverse measures of detachment (e.g., rumination or work-related thoughts during non-work time) were recoded. We used prospective and lagged correlations in longitudinal studies and between-person associations of aggregated measures in diary studies. Moreover, we calculated composite effect sizes according to the formula of Hunter and Schmidt (2004) wherever multiple associations between constructs of interest were reported. This occurred in three cases: (a) correlations between multiple measures of psychological detachment (e.g., positive and negative work reflection) and the outcome, (b) correlations with multiple outcome variables of one construct (e. g, sleep latency and sleep quality), and (c) correlations with constructs that are measured several times (e.g., emotional exhaustion after 6 months and 1 year). This procedure is necessary given that independency of effect sizes is required to pool those (Borenstein et al., 2009).

We aggregated effect sizes according to the approach suggested by Hedges and Olkin (1985) and calculated sample size weighted mean correlations $\overset{-}{r}$ with a random-effects model (Borenstein et al., 2009). For all relationships we report the number of studies *k*, the cumulative sample size *N*, the sample size weighted mean correlation $\overset{-}{r}$ and its 95% confidence interval (CI). If the 95% CI excludes zero, the mean effect is significant with *p* < 0.05 (two-tailed). We used Cohen's (1992) rules of thumb for effect size evaluation (*r* ≥ 0.1 small, *r* ≥ 0.3 medium, and *r* ≥ 0.5 large) and only interpreted pooled correlations based on *k*s ≥ 5.

We calculated *I*^2^-statistics to estimate the heterogeneity of effect sizes. The *I*^2^ indicates the proportion of the observed variance reflecting real differences in effect sizes and is a measure for the inconsistency of findings across the aggregated study effects (Borenstein et al., 2009). Values of *I*^2^ ≥ 25% indicate at least some (25% = low, 50% = moderate, 75% = high) heterogeneity, and thus point to the potential existence of moderator variables. In addition, we report 95% prediction intervals (PI; Borenstein et al., 2009; IntHout et al., 2016) indicating the distribution of true effects around the pooled mean effect. While the 95% CI is a measure for the precision of the mean effect, the 95% PI describes the range of effects that may be expected in future studies. We examined effects of categorical moderators with subgroup analysis (*Q*_Between_-statistics). We applied mixed effects meta-regression modeling (unrestricted maximum likelihood method) for interval scaled moderators. We conducted moderator analyses only for variables with a sufficient number of studies (*k*s = 10) and interpreted categorical moderator effects only in cases of subgroup-*k*s ≥ 5. For significant moderator effects we also report the τ^2^-based index *R*^2^ indicating the amount of between-study variance that is explained by the moderator variable (Borenstein et al., 2009). As multiple comparisons in meta-analytical moderator analyses might inflate type I error rates (Cafri et al., 2010) these results should be interpreted with caution.

In addition, we present results from several analyses regarding the sensitivity of results and the impact of a potential publication bias.

All analyses were conducted with Comprehensive Meta-Analysis software 2.2 (Biostat, Inc, Englewood, NJ).

## Results

### Description of samples and measures

The mean age of employees in the samples was 39.9 years and the mean percentage of females in the studies was 55%. Fifty-four studies (over 50%) were published between 2013 and 2015. Most samples were from Europe (*k* = 63). Study samples sizes were left skewed distributed with *Mdn* = 143 and *M* = 419 (range: 48–5210) participants.

Most of the studies assessed detachment from work as an affectively *neutral* type of absent work-related thoughts during non-work time (*k* = 75) and used the detachment scale of the Recovery Experience Questionnaire (*k* = 67; Sonnentag and Fritz, 2007). We found only a few studies that assessed detachment as absence or presence of negative (*negative valence, k =* 17) or positive (*positive valence, k* = 8) work-related thoughts during non-work time.

For all core constructs the measures' mean Cronbach's αs were > 0.75 (detachment_negative_: 0.87, detachment_neutral_: 0.86, detachment_positive_: 0.87, physical discomfort: 0.80, sleep 0.76, life satisfaction: 0.87, exhaustion: 0.87, burnout-others: 0.80, well-being: 0.84, fatigue: 0.85, affect: 0.84, state of recovery: 0.92, work motivation: 0.88, task performance: 0.81, contextual performance: 0.82, creativity: 0.84, job demands: 0.81, job resources: 0.80, work-related activities during non-work time: 0.78, negative affectivity/neuroticism: 0.81, heavy work investment: 0.78).

### Outcomes of detachment

We aggregated data from *k* = 75 studies (*N* = 29,587; 319 effect sizes) to examine relationships between detachment and health, state well-being, work motivation, and work performance. Table 1 presents the pooled effect sizes and heterogeneity statistics (forest plots are depicted in the Supplementary Material).

**Table 1:** **Meta-analytic correlations between detachment from work and different outcomes**.

**Variable**	***k***	***N***	***$\overset{-}{r}$***	**95% CI**		**95% PI**		***I*^2^**
				***LL***	***UL***	***LL***	***UL***	
**HEALTH**								
Burnout (exhaustion)	23	7007	−0.36	−0.42	−0.30	−0.60	−0.07	85.76
Life satisfaction^a^	5	1236	0.32	0.11	0.50	−0.46	0.82	91.96
Well-being	16	11,133	0.32	0.26	0.37	0.01	0.57	84.72
Sleep	18	12,028	0.30	0.22	0.38	−0.09	0.61	94.55
Physical discomfort^a^	6	5544	−0.23	−0.31	−0.15	−0.12	0.53	86.70
Burnout (others)^a^	7	1867	−0.14	−0.28	−0.01	−0.56	0.33	88.53
Physiological activation^a^^,^ ^b^	3	219	0.03	−0.12	0.17	−0.80	0.82	13.26
**STATE WELL-BEING**								
Fatigue	17	12,510	−0.42	−0.52	−0.30	−0.78	0.17	97.86
Affect	24	5145	0.28	0.21	0.34	−0.03	0.53	79.99
State of recovery^a^	7	2824	0.31	0.18	0.43	−0.14	0.66	88.76
**WORK MOTIVATION**								
Work motivation	11	6083	0.04	−0.06	0.13	−0.30	0.36	90.29
**WORK PERFORMANCE**								
Task performance^a^	8	4551	0.09	0.03	0.14	−0.05	0.22	42.72
Contextual performance^a^	5	2106	−0.13	−0.21	−0.05	−0.36	0.12	58.58
Creativity^a^	5	2398	−0.11	−0.19	−0.04	−0.33	0.11	62.48

*k, number of independent effect sizes; N, sample size; $\overset{-}{r}$, sample size weighted mean correlation; CI, confidence interval of $\overset{-}{r}$; PI, prediction interval of $\overset{-}{r}$; LL, lower limit of the 95% CI/PI; UL, upper limit of the 95% CI/PI; I^2^, (Variance_Between_/Variance_Total_) × 100%*.

a *Analysis of moderator variables rejected for k < 10*.

b *Direct effect should be interpreted with caution as it is based on a low study sample size (k < 5)*.

We found several significant mean correlations between detachment and health. In particular, detachment from work was positively associated with psychological (moderate effects with a range of 0.30 ≤ $\left|\overset{-}{r}\right|$ ≤ 0.36: lower exhaustion, higher life satisfaction, higher well-being, better sleep; small effect with *r* = −0.14: lower other burnout symptoms) and physical health (small effect: *r* = −0.23: lower physical discomfort). Only three studies examined relationships between detachment and physiological stress indicators and the average effect was non-significant. Our analysis further revealed mean small to moderate positive correlations between detachment and state well-being (lower fatigue, better affect, and better state of recovery; range: 0.28 ≤ $|\overset{-}{r}|\leq$ 0.42). There was no significant mean correlation with work motivation. Moreover, our analysis revealed significant but small mean relationships with measures of work performance. As predicted, detachment was positively related to task performance. However, unexpectedly, we found, on average, significant *negative* correlations with contextual performance and creativity.

Except for physiological stress indicators, *I*^2^-statistics indicated moderate (task performance, contextual performance, creativity) and high (exhaustion, life satisfaction, well-being, sleep, physical discomfort, burnout-others, fatigue, affect, state of recovery) heterogeneity of effect sizes (see Table 1). This large dispersion in effect sizes between studies is also reflected by 95% PIs crossing zero for most relationships. Only for relationships between detachment and exhaustion and detachment and well-being 95% PIs indicated that there is a chance of 95% that a new study will report a negative or rather positive correlation. However, we note that on the primary study level (see the forest plots in the Supplementary Material) the direction of effects were fully (e.g., exhaustion, life satisfaction, well-being, physical discomfort, fatigue, contextual performance) or predominantly (e.g., sleep, burnout others, affect, state of recovery, task performance, creativity) consistent for most of the examined relationships.

### Antecedents of detachment

#### Work characteristics

We aggregated data from *k* = 61 studies (*N* = 28,588; 197 effect sizes) to examine relationships between work characteristics and detachment. Table 2 presents the pooled effect sizes and heterogeneity statistics (forest plots are depicted in the Supplementary Material).

**Table 2:** **Meta-analytic correlations between work characteristics and detachment from work**.

**Variable**	***k***	***N***	**$\overset{-}{r}$**	**95% CI**		**95% PI**		***I*^2^**
				***LL***	***UL***	***LL***	***UL***	
**JOB DEMANDS**								
Composite measure	60	28,507	−0.25	−0.29	−0.22	−0.49	0.02	89.34
Quantitative demands	33	16,687	−0.28	−0.32	−0.23	−0.49	−0.02	86.89
Social conflicts	12	7233	−0.25	−0.36	−0.14	−0.62	0.20	95.31
Emotional demands^a^	7	9534	−0.22	−0.28	−0.15	−0.40	−0.02	83.55
Working time	30	10,464	−0.17	−0.21	−0.12	−0.38	0.07	79.31
Role stressors^a^	9	5684	−0.12	−0.18	−0.07	−0.28	0.04	67.74
**JOB RESOURCES**								
Composite measure	24	15,010	0.10	0.04	0.17	−0.20	0.39	91.71
Social support^a^	7	8871	0.21	0.13	0.28	−0.06	0.45	89.51
Job control	20	11,570	0.06	0.02	0.10	−0.10	0.21	70.85

*k, number of independent effect sizes; N, sample size; $\overset{-}{r}$, sample size weighted mean correlation; CI, confidence interval of $\overset{-}{r}$; PI, prediction interval of $\overset{-}{r}$; LL, lower limit of the 95% CI/PI; UL, upper limit of the 95% CI/PI; I^2^, (Variance_Between_/Variance_Total_) × 100%*.

a *Analysis of moderator variables rejected for k < 10*.

Results indicated that detachment from work correlated, on average, significantly negatively with job demands and significantly positively with job resources. Both pooled effect size estimates were of small magnitude (range: 0.10 ≤ $\left|\overset{-}{r}\right|$ ≤ 0.25) and differed significantly [*Q*_Between_(1) = 91.03, *p* < 0.001]. Specifically, the average correlation between job demands and detachment was significantly stronger than between job resources and detachment (no overlap of 95% CIs for absolute values of both *r*s). Furthermore, these average correlations were also significant, when using more proximal indicators of both types of work characteristics (see Table 2).

Contrast analyses revealed that average correlations to detachment differed significantly between different types of job demands [*Q*_Between_(4) = 14.57, *p* < 0.01] and job resources [*Q*_Between_(1) = 11.74, *p* = 0.001]. For job demands, we found that the average correlation between detachment and quantitative demands was significantly stronger than between detachment and working hours [*Q*_Between_(1) = 10.82, *p* = 0.001] and role stressors [*Q*_Between_(1) = 9.23, *p* < 0.01]. Moreover, the average correlation between detachment and emotional demands was significantly stronger than between detachment and role stressors [*Q*_Between_(1) = 4.48, *p* < 0.05]. For job resources, we found that detachment was, on average, stronger positively related to social support than to job control [*Q*_Between_(1) = 11.72, *p* = 0.001]. The *I*^2^-statistics indicated a moderate to large heterogeneity of effect sizes (all *I*^2^s > 67%) for all relationships between work characteristics and detachment from work. This large between-study dispersion of effect sizes is also reflected by 95% PIs crossing zero for most relationships. Only for relationships between quantitative demands and detachment and emotional demands and detachment the 95% PIs indicated a high chance that future studies will report negative correlations. Again, we found rather consistently reported directions of effects for different kinds of job demands and also for social support as job resource (see forest plots in the Supplementary Material).

#### Work-related activities during non-work time

We aggregated data from *k* = 17 studies (*N* = 4736; 21 effect sizes; a forest plot is depicted in the Supplementary Material). Most of these studies used single-item measures (*k* = 11). We found an average moderate and negative correlation between engagement in work-related activities during non-work time and detachment from work ($\overset{-}{r}$ = −0.31, 95% CI [−0.38, −0.23]). Study effect sizes were heterogeneous (*I*^2^ = 82.31%) but all aggregated primary studies reported negative correlations. The 95% PI [−0.56, 0.00] indicated a high chance of finding negative correlations to detachment in future studies.

#### Person characteristics

We aggregated data from *k* = 63 studies (*N* = 21,208; 137 effect sizes) to estimate the associations between person characteristics and detachment from work (forest plots are depicted in the Supplementary Material). Age (*k* = 43, *N* = 14,408, $\overset{-}{r}$ = −0.02, 95% CI [−0.06, 0.01], 95% PI [−0.19, 0.15], *I*^2^ = 68.21%) and gender (*k =* 44, *N* = 14,598, $\overset{-}{r}$ = 0.03, 95% CI [−0.01, 0.07], 95% PI [−0.18, 0.24], *I*^2^ = 76.90%) were, on average, unrelated to detachment from work. However, the moderate to large heterogeneity (*I*^2^) and dispersion of effect sizes (95% PIs crossing zero) point to the existence of additional moderator variables.

Detachment was, on average, significantly negatively associated with negative affectivity/neuroticism (*k* = 17, *N* = 9372, $\overset{-}{r}$ = −0.22, 95% CI [−0.35, −0.08], 95% PI [−0.69, 0.39], *I*^2^ = 97.46%; small effect) and heavy work investment (*k* = 5, *N* = 2.801, $\overset{-}{r}$ = −0.32, 95% CI [−0.39, −0.25], 95% PI [−0.49, −0.19], *I*^2^ = 58.31%; moderate effect). Again, we found a substantial amount of heterogeneity for both relationships. Only for the relationship between heavy work investment and detachment the range of the prediction interval indicated a high chance that future studies will report negative correlations. This is also underlined on the primary study level as all five studies reported significant negative correlations (see forest plot in the Supplementary Material).

### Moderator analyses

For most of the investigated relationships between detachment and possible outcomes and antecedents, we found a substantial amount of heterogeneity and dispersion in effect sizes. Therefore, we examined potential moderating effects of several study-related (i.e., study location, study design) and demographic variables (i.e., mean age and gender distribution in the samples), as well as affective valence of work-related thoughts during non-work time. Table 3 provides an overview of results. Note that in several cases moderator analyses were not possible because of an insufficient number of studies (low total *k*s or subgroup-*k*s; see also results above in Tables 1, 2).

**Table 3:** **Summary of results for moderator analyses**.

**Moderator**		***k***	***ES***	**95% CI**		***Q***	***I^2^***
				***LL***	***UL***		
**OUTCOMES**							
**Sleep**							
***Location***							
Europe	*r*	16	0.31	0.22	0.40	0.40^a^	94.65
USA/Canada	*r*	2	0.23	−0.02	0.46		0.00
***Study design***							
Longitudinal	*r*	1	0.29	−0.05	0.57	7.37^*^^a^	0.00
CS-one time	*r*	9	0.39	0.29	0.49		96.54
CS-diary	*r*	8	0.17	0.04	0.30		33.70
***Mean age (years)***	*b*	17	0.02	−0.01	0.04	2.20	
***Females (%)***	*b*	18	0.00	−0.01	0.00	2.36	
***Affective valence***							
Negative	*r*	8	0.41	0.32	0.50	9.00^*^^a^	96.30
Neutral	*r*	11	0.22	0.12	0.32		56.03
Positive	*r*	1	0.14	−0.16	0.41		0.00
**Well-Being**							
***Location***							
Europe	*r*	11	0.31	0.23	0.37	2.65^a^	67.51
USA/Canada	*r*	2	0.46	0.28	0.61		94.33
Others	*r*	3	0.31	0.18	0.43		92.01
***Study design***							
Longitudinal	*r*	2	0.19	0.05	0.32	10.62^**^^a^	78.56
CS-one time	*r*	11	0.30	0.24	0.36		79.53
CS-diary	*r*	3	0.49	0.37	0.60		87.27
***Mean age (years)***	*b*	14	−0.01	−0.02	0.01	0.78	
***Females (%)***	*b*	16	0.00	0.00	0.01	2.41	
***Affective valence***							
Negative	*r*	1	0.31	0.07	0.52	0.01^a^	0.00
Neutral	*r*	15	0.32	0.25	0.39		83.97
**Burnout (Exhaustion)**							
***Location***							
Europe	*r*	18	−0.38	−0.44	−0.32	2.29^a^	82.27
USA/Canada	*r*	3	−0.27	−0.42	−0.09		91.48
Others	*r*	2	−0.31	−0.47	−0.12		28.75
***Study design***							
Longitudinal	*r*	4	−0.34	−0.47	−0.19	0.25^a^	79.28
CS-one time	*r*	15	−0.37	−0.44	−0.30		89.50
CS-diary	*r*	4	−0.34	−0.49	−0.17		38.67
***Mean age (years)***	*b*	21	−0.01	−0.02	0.00	1.23	
***Females (%)***	*b*	23	0.00	0.00	0.01	1.50	
***Affective valence***							
Negative	*r*	3	−0.53	−0.64	−0.39	6.29^*^^a^	93.12
Neutral	*r*	21	−0.35	−0.41	−0.28		84.78
Positive	*r*	1	−0.25	−0.50	0.04		0.00
**Fatigue**							
***Location***							
Europe	*r*	16	−0.43	−0.54	−0.31	0.92^a^	97.93
USA/Canada	*r*	1	−0.17	−0.62	0.37		0.00
***Study design***							
Longitudinal	*r*	1	−0.34	−0.71	0.18	4.37^a^	0.00
CS-one time	*r*	10	−0.50	−0.62	−0.37		98.58
CS-diary	*r*	6	−0.25	−0.45	−0.02		67.32
***Mean age (years)***	*b*	16	0.01	0.00	0.05	0.53	
***Females (%)***	*b*	17	0.00	−0.01	0.01	0.09	
***Affective valence***							
Negative	*r*	5	−0.56	−0.70	−0.39	4.16^a^	99.17
Neutral	*r*	13	−0.36	−0.48	−0.23		89.58
Positive	*r*	1	−0.25	−0.64	0.24		0.00
**State Affect**							
***Location***							
Europe	*r*	19	0.28	0.21	0.35	3.51^a^	81.99
USA/Canada	*r*	4	0.18	0.08	0.28		0.00
Others	*r*	1	0.48	0.35	0.60		0.00
***Study design***							
CS-one time	*r*	5	0.35	0.22	0.48	1.75	90.17
CS-diary	*r*	19	0.25	0.17	0.33		74.98
***Mean age (years)***	*b*	24	−0.01	−0.02	0.01	0.41	
***Females (%)***	*b*	23	0.00	0.00	0.01	1.60	
***Affective valence***							
Negative	*r*	6	0.38	0.26	0.50	17.05^***^	87.51
Neutral	*r*	19	0.29	0.22	0.36		61.74
Positive	*r*	5	−0.02	−0.18	0.13		92.60
**Work Motivation**							
***Location***							
Europe	*r*	9	0.10	0.02	0.17	13.00^**^^a^	64.63
USA/Canada	*r*	1	−0.24	−0.42	−0.04		0.00
Others	*r*	1	−0.08	−0.25	0.09		0.00
***Study design***							
Longitudinal	*r*	2	0.06	−0.16	0.28	1.14^a^	0.00
CS-one time	*r*	6	0.00	−0.14	0.13		94.25
CS-diary	*r*	3	0.13	−0.09	0.34		83.22
***Mean age (years)***	*b*	9	0.00	−0.02	0.01	0.13	
***Females (%)***	*b*	11	0.02	0.01	0.02	17.69^***^	
***Affective valence***							
Neutral	*r*	11	0.04	−0.06	0.13	0.00	90.29
**ANTECEDENTS**							
**Job Demands**							
***Location***							
Europe	*r*	44	−0.25	−0.30	−0.21	0.02	88.01
USA/Canada	*r*	7	−0.25	−0.36	−0.13		43.96
Others	*r*	9	−0.26	−0.35	−0.17		91.08
***Study design***							
Longitudinal	*r*	3	−0.32	−0.47	−0.15	1.11^a^	90.59
CS-one time	*r*	35	−0.26	−0.31	−0.21		92.31
CS-diary	*r*	22	−0.23	−0.30	−0.16		74.64
***Mean age (years)***	*b*	55	0.00	−0.01	0.01	0.02	
***Females (%)***	*b*	59	0.00	0.00	0.00	0.83	
***Affective valence***							
Negative	*r*	12	−0.32	−0.40	−0.24	4.80^a^	93.18
Neutral	*r*	49	−0.24	−0.29	−0.20		80.81
Positive	*r*	4	−0.14	−0.29	0.01		98.57
**Quantitative Demands**							
***Location***							
Europe	*r*	23	−0.29	−0.35	−0.24	1.49	83.46
USA/Canada	*r*	5	−0.21	−0.33	−0.09		69.29
Others	*r*	5	−0.26	−0.36	−0.16		85.99
***Study design***							
Longitudinal	*r*	3	−0.37	−0.50	−0.22	3.55^a^	85.52
CS-one time	*r*	20	−0.28	−0.34	−0.23		90.49
CS-diary	*r*	10	−0.21	−0.30	−0.11		47.86
***Mean age (years)***	*b*	29	−0.01	−0.02	0.00	3.23	
***Females (%)***	*b*	33	0.00	0.00	0.00	0.25	
***Affective valence***							
Negative	*r*	5	−0.33	−0.45	−0.20	1.88^a^	69.41
Neutral	*r*	28	−0.27	−0.33	−0.21		81.55
Positive	*r*	3	−0.18	−0.35	0.01		98.94
**Social Conflicts**							
***Location***							
Europe	*r*	7	−0.24	−0.38	−0.08	0.30^a^	96.70
USA/Canada	*r*	2	−0.23	−0.50	0.08		65.69
Others	*r*	3	−0.31	−0.51	−0.08		93.04
***Study design***							
CS-one time	*r*	7	−0.27	−0.40	−0.12	0.13	96.77
CS-diary	*r*	5	−0.23	−0.40	−0.04		90.70
***Mean age (years)***	*b*	12	0.01	−0.01	0.03	1.74	
***Females (%)***	*b*	11	0.00	0.00	0.01	0.06	
***Affective valence***							
Negative	*r*	3	−0.47	−0.56	−0.37	22.35^***^^a^	94.15
Neutral	*r*	9	−0.16	−0.24	−0.08		60.28
**Working Time**							
***Location***							
Europe	*r*	24	−0.18	−0.23	−0.13	0.91	72.46
USA/Canada	*r*	6	−0.13	−0.22	−0.04		81.80
***Study design***							
Longitudinal	*r*	3	−0.19	−0.33	−0.04	2.12^a^	91.71
CS-one time	*r*	17	−0.14	−0.20	−0.08		80.70
CS-diary	*r*	10	−0.22	−0.30	−0.13		63.16
***Mean age (years)***	*b*	29	0.01	0.00	0.02	1.08	
***Females (%)***	*b*	30	0.00	0.00	0.00	0.02	
***Affective valence***							
Negative	*r*	4	−0.09	−0.21	0.03	1.76^a^	0.00
Neutral	*r*	28	−0.17	−0.22	−0.13		80.03
Positive	*r*	2	−0.20	−0.34	−0.04		52.45
**Job Resources**							
***Location***							
Europe	*r*	14	0.08	0.00	0.16	2.03	66.64
USA/Canada	*r*	5	0.09	−0.05	0.22		94.17
Others	*r*	5	0.18	0.06	0.29		89.73
***Study design***							
Longitudinal	*r*	2	0.13	−0.09	0.34	0.22^a^	0.00
CS-one time	*r*	18	0.11	0.03	0.18		93.69
CS-diary	*r*	4	0.07	−0.11	0.24		42.63
***Mean age (years)***	*b*	21	0.00	−0.01	0.01	0.19	
***Females (%)***	*b*	23	0.00	0.00	0.00	0.74	
***Affective valence***							
Negative	*r*	5	0.15	0.05	0.24	6.72^*^^a^	0.00
Neutral	*r*	19	0.07	0.02	0.13		77.75
Positive	*r*	3	0.25	0.12	0.37		97.09
**Job control**							
***Location***							
Europe	*r*	13	0.06	0.00	0.12	4.27^a^	60.31
USA/Canada	*r*	3	−0.07	−0.21	0.07		0.00
Others	*r*	4	0.10	0.01	0.19		89.79
***Study design***							
Longitudinal	*r*	2	0.04	−0.09	0.18	0.94^a^	71.45
CS-one time	*r*	16	0.07	0.02	0.11		74.74
CS-diary	*r*	2	−0.03	−0.21	0.16		9.21
***Mean age (years)***	*b*	17	0.00	−0.01	0.01	0.00	
***Females (%)***	*b*	20	0.00	0.00	0.00	0.44	
***Affective valence***							
Negative	*r*	3	0.11	0.00	0.21	1.29^a^	77.22
Neutral	*r*	17	0.05	0.00	0.10		72.76
Positive	*r*	2	0.11	−0.05	0.25		0.00
**Work Related-Activities**							
***Location***							
Europe	*r*	14	−0.28	−0.35	−0.20	3.05^a^	80.41
USA/Canada	*r*	3	−0.42	−0.54	−0.28		39.49
***Study design***							
CS-one time	*r*	7	−0.34	−0.44	−0.23	0.78	91.96
CS-diary	*r*	10	−0.27	−0.37	−0.17		38.32
***Mean age (years)***	*b*	17	0.01	−0.01	0.01	0.03	
***Females (%)***	*b*	17	0.00	−0.01	0.00	1.13	
***Affective valence***							
Negative	*r*	2	−0.29	−0.49	−0.06	0.03^a^	85.71
Neutral	*r*	15	−0.31	−0.38	−0.23		83.18
**Age**							
***Location***							
Europe	*r*	31	−0.03	−0.07	0.01	0.64^a^	59.19
USA/Canada	*r*	9	−0.01	−0.08	0.06		72.22
Others	*r*	3	0.01	−0.09	0.11		0.00
***Study design***							
Longitudinal	*r*	1	−0.04	−0.23	0.16	0.12^a^	0.00
CS-one time	*r*	21	−0.02	−0.06	0.03		77.24
CS-diary	*r*	21	−0.03	−0.09	0.03		49.99
***Mean age (years)***	*b*	42	0.00	−0.01	0.00	0.48	
***Females (%)***	*b*	41	0.00	0.00	0.00	1.09	
***Affective valence***							
Negative	*r*	6	0.05	−0.02	0.11	7.89^*^^a^	48.80
Neutral	*r*	37	−0.03	−0.06	−0.00		50.53
Positive	*r*	4	0.05	−0.02	0.12		31.37
**Gender**							
***Location***							
Europe	*r*	32	0.02	−0.03	0.06	2.61^a^	74.80
USA/Canada	*r*	9	0.04	−0.04	0.12		71.37
Others	*r*	3	0.13	0.00	0.24		0.00
***Study design***							
Longitudinal	*r*	1	0.13	−0.13	0.37	1.09^a^	0.00
CS-one time	*r*	23	0.02	−0.03	0.07		85.89
CS-diary	*r*	20	0.05	−0.02	0.12		33.99
***Mean age (years)***	*b*	42	0.00	−0.01	0.01	0.44	
***Females (%)***	*b*	43	0.00	0.00	0.00	0.63	
***Affective valence***							
Negative	*r*	4	−0.03	−0.16	0.10	1.15^a^	91.83
Neutral	*r*	40	0.03	−0.01	0.08		75.76
Positive	*r*	3	−0.02	−0.16	0.13		92.27
**Negative Affectivity/Neuroticism**							
***Location***							
Europe	*r*	11	−0.26	−0.41	−0.11	1.00^a^	97.34
USA/Canada	*r*	5	−0.14	−0.37	0.10		0.00
Others	*r*	1	−0.08	−0.54	0.43		0.00
***Study design***							
Longitudinal	*r*	3	−0.19	−0.46	0.12	4.51^a^	89.10
CS-one time	*r*	6	−0.38	−0.55	−0.18		98.90
CS-diary	*r*	8	−0.09	−0.28	0.10		0.00
***Mean age (years)***	*b*	17	−0.01	−0.04	0.01	1.68	
***Females (%)***	*b*	16	0.01	0.00	0.01	4.58^*^	
***Affective valence***							
Negative	*r*	7	−0.40	−0.50	−0.29	27.32^***^^a^	96.65
Neutral	*r*	12	−0.16	−0.25	−0.05		68.90
Positive	*r*	4	0.13	−0.04	0.29		0.00

*CS, cross-sectional; r, correlation coefficient; b, unstandardized regression weight of the slope (meta-regression); k, number of independent effect sizes; ES, effect size; 95% CI, 95% confidence interval of ES; LL, lower limit of the 95% CI; UL, upper limit of the 95% CI; Q, results of the Q-test for moderator effects; I^2^, (Variance_Between_/Variance_Total_) × 100% (only for subgroups of moderators)*.

* *p ≤ 0.05*;

** *p ≤ 0.01*;

*** *p ≤ 0.001*.

a *Moderator effects should be not interpreted or with caution as there are fewer than five studies in one or more subgroups of the moderator*.

#### Detachment-outcomes relationships

Due to the small number of subgroup samples, it was not possible to examine the moderating role of *study location*. An insufficient number of longitudinal studies also restricted moderator analyses for *study design*. However, we found that the average correlations between detachment from work and sleep, as well as detachment and fatigue were significantly stronger in cross-sectional studies with one measurement occasion (sleep: $\overset{-}{r}$ = 0.40, 95% PI [−0.01, 0.69]; fatigue: $\overset{-}{r}$ = −0.50, 95% PI [−0.84, 0.10]) than in diary studies (sleep: $\overset{-}{r}$ = 0.17, 95% PI [−0.07, 0.39], *Q*_Between_(1) = 7.36, *p*=0.007, *R*^2^ = 0.19; fatigue: $\overset{-}{r}$ = −0.25, 95% PI [−0.66, 0.27], *Q*_Between_(1) = 6.20, *p* = 0.013, *R*^2^ = 0.09). Average effect sizes were significant under both study designs for both outcomes and heterogeneity of effect sizes was lower in diary studies. The relationship between detachment and affect was not moderated by study design. At least for relationships to well-being and exhaustion, the average correlations in longitudinal studies were significant.

*Samples' mean age* did not significantly moderate the relationships between detachment and sleep, well-being, exhaustion, fatigue, affect, and work motivation. We found that *samples' percentage of females* only moderated the relationship between detachment and work motivation. The mean positive relationship between both variables increased with more females in the samples (*b* = 0.015, *SE* = 0.004, *Z* = 4.21, *p* < 0.001, *R*^2^ = 0.74).

Due to the small number of samples (low subgroup-*k*s < 5), we could only analyze the moderating role of *valence* for sleep, fatigue, and affect as outcomes. In addition, for sleep and fatigue as outcomes fewer than five studies assessed detachment as absence from positive work-related thoughts. In both cases, we only could contrast results from studies assessing detachment as an affectively neutral construct and those assessing detachment as absence from negative work-related thoughts. The average positive correlation between detachment and sleep was significantly stronger in studies assessing detachment as absence from negative work-related thoughts during non-work time ($\overset{-}{r}\;=$ 0.42, 95% PI [0.02, 0.69]) than assessing it as an affectively neutral concept ($\overset{-}{r}$ = 0.22, 95% PI [0.00, 0.42], *Q*_Between_(1) = 7.69, *p* = 0.006, *R*^2^ = 0.28). However, heterogeneity only decreased for the neutral type of detachment. Under both conditions 95% prediction intervals indicate a high chance that future studies will report positive correlations for the relationship between detachment and sleep. There was some indication that the negative relationship between detachment and fatigue was stronger in studies assessing detachment as absence from negative work-related thoughts ($\overset{-}{r}$ = −0.56, 95% PI [−0.92, 0.29]) than as affectively neutral concept ($\overset{-}{r}$ = −0.36, 95% PI [−0.68, 0.07]). However, this moderator effect was not significant [*Q*_Between_(1) = 3.61, *p* = 0.056, *R*^2^ = 0.11]. Moreover, we found a significant moderator effect of valence for affect as outcome (*R*^2^ = 0.06). Specifically, mean relationships between detachment and affect were stronger and only significant when accessing detachment as absence from negative work-related thoughts during non-work time ($\overset{-}{r}$ = 0.38, 95% PI [−0.12, 0.73]) and as affectively neutral concept ($\overset{-}{r}$ = 0.29, 95% PI [0.08, 0.48]) than as absence from positive work-related thoughts during non-work time ($\overset{-}{r}$ = −0.02, 95% PI [−0.59, 0.56]). Results of contrast analyses revealed both subgroup differences as significant [negative vs. positive: *Q*_Between_(1) = 9.01, *p*=0.003; neutral vs. positive: *Q*_Between_(1) = 13.35, *p* < 0.001; negative vs. neutral: *Q*_Between_(1) = 2.15, *p* = 0.142]. Only for affectively neutral measures of detachment heterogeneity was reduced and the 95% PI indicated a high chance that future studies will report positive correlations for relationships between detachment and affect.

The relationship between detachment and sleep was significantly moderated by two variables: study design and valence. A further analysis showed that both variables were not significantly interrelated [${\text{X}}_{\left(1\right)}^{2}=1.17$, *p* = 0.367]. Therefore, it is less likely that both moderator effects are based on confounding and should be interpreted independently.

#### Antecedents-detachment relationships

Study-related (location, study design) and demographic (mean age, percentage of females) variables did not moderate the relationships between detachment and work-related antecedents (i.e., job demands and job resources). At least for job demands, quantitative demands, and working time we found significant mean correlations in longitudinal studies.

As expected, valence of work-related thoughts moderated the relationship between general job demands and detachment. Because of a low subgroup sample size for positive valence we only could contrast the negative and neutral condition [*Q*_Between_(1) = 3.94, *p* = 0.047, *R*^2^ = 0.29]. More specifically, mean correlations were stronger negative for detachment assessed as absence of negative work-related thoughts during non-work time ($\overset{-}{r}$ = −0.32, 95% PI [−0.43, 0.20]) than for affectively neutral measures ($\overset{-}{r}$ = −0.24, 95% PI [−0.45, −0.01]). We could not analyze the moderating impact of study location and valence for the relationship between work-related activities during non-work time and detachment. We found no moderating impact of study design, mean age, and percentage of females for this relationship. Study-related (location and design) and demographic (mean age, percentage of females) variables did moderate the relationships between detachment and age and gender. However, our analyses revealed that the average correlation between age and detachment was significantly negative for affectively neutral measures of detachment ($\overset{-}{r}$ = −0.03, 95% PI [−0.16, 0.10]) but insignificant for studies assessing detachment as absence of negative work-related thoughts ($\overset{-}{r}$ = 0.05, 95% PI [−0.11, 0.19], *Q*_Between_(1) = 4.43, *p* = 0.035, *R*^2^ = 0.36).

Moreover, we found that study design [*Q*_Between_(1) = 4.30, *p* = 0.038, *R*^2^ = 0.17], studies' gender composition (*R*^2^ = 0.58), and valence of detachment [*Q*_Between_(1) = 9.50, *p* = 0.002, *R*^2^=0.49] moderated the relationship between negative affectivity/neuroticism and detachment. More specifically, the average correlation was only significantly negative in cross-sectional studies with one measurement occasion ($\overset{-}{r}$ = −0.38, 95% PI [−0.85, 0.42]) but not in diary studies ($\overset{-}{r}$ = −0.09, 95% PI [−0.32, 0.15]). The strength of negative relationship between negative affectivity/neuroticism and detachment decreased with more females in the study samples (*b* = 0.008, *SE* = 0.004, *Z* = 2.14, *p* = 0.032). Moreover, the average correlation between both variables was stronger negative in studies assessing detachment as absence of negative work-related thoughts during non-work time ($\overset{-}{r}$ = −0.40, 95% PI [−0.73, 0.07]) than assessing it as an affectively neutral concept ($\overset{-}{r}$ = −0.16, 95% PI [−0.46, 0.19]). We further analyzed a possible confounding of these significant moderating variables. However, intercorrelations were not significant (study design-females: |*r*| = 0.33, *p* = 0.257; study design-valence: ${\text{X}}_{\left(1\right)}^{2}$ = 0.64, *p* = 0.607; gender-valence: |*r*| = 0.15, *p* = 0.547). This suggests interpreting the three moderator effects independently.

### Sensitivity analyses

We conducted sensitivity analyses to further examine the robustness of our results. At first, we aimed to analyze the impact of potential outliers in effect sizes. For none of the examined relationships we found evidence for such outliers (all primary study *r*s between ± 3 *SD* from the *M*). Second, we examined how average effect sizes would change if one study out of the whole set of studies for each relationship is excluded from the analysis. Only for two relationships the significance of the average correlation would change (Table 4). Regarding the relationship between detachment and burnout (outhers), we found that the exclusion of each out of four studies (Els et al., 2015; Rivkin et al., 2015 Sample 1 and 2; Sonnentag and Fritz, 2007) would eliminate significance of the average correlation. However, the size of the average effects would be reduced only slightly. Regarding the relationship between gender and detachment, we found that the exclusion of each out of three studies (Moreno-Jiménez et al., 2009a; Kinnunen et al., 2010; Querstret and Cropley, 2012) would bring the average correlation to significance, however, only coupled with a small increase in the average effect size. Furthermore, regarding the relationship between detachment and life satisfaction an exclusion of two studies would largely reduce (Moreno-Jiménez et al., 2009a) or increase (Park and Fritz, 2015) the average effect size. Taken together, we conclude that our results are not biased by extreme outliers in effect sizes and that for most of the examined relationships the presented average effect size estimates are quite robust with regard to removing effect sizes of single studies from the overall analyses.

**Table 4:** **Summary of results for the impact of removing one study out of the whole set of studies**.

	**Lowest** $\overset{-}{r}$			**Highest** $\overset{-}{r}$		
		**95% CI**			**95% CI**	
	**$\overset{-}{r}$**	***LL***	***UL***	**$\overset{-}{r}$**	***LL***	***UL***
**OUTCOMES OF DETACHMENT**						
**Health**						
Exhaustion	−0.38	−0.43	−0.33	−0.35	−0.41	−0.29
Life satisfaction	0.24	0.07	0.39	0.39	0.21	0.54
Well-being	0.30	0.24	0.35	0.33	0.28	0.39
Sleep	0.28	0.21	0.35	0.32	0.23	0.40
Physical discomfort	−0.26	−0.36	−0.16	−0.19	−0.24	−0.13
Burnout (others)	−0.19	−0.31	−0.06	−0.10	−0.23	0.04
Physiological activation	−0.04	−0.20	0.12	0.08	−0.16	0.31
**State Well-Being**						
Fatigue	−0.43	−0.54	−0.31	−0.39	−0.46	−0.30
Affect	0.26	0.20	0.32	0.29	0.23	0.35
State of recovery	0.26	0.14	0.38	0.36	0.25	0.46
Work motivation	0.01	−0.09	0.10	0.07	−0.03	0.16
**Work Performance**						
Task performance	0.07	0.00	0.14	0.10	0.06	0.14
Contextual performance	−0.18	−0.24	−0.11	−0.10	−0.16	−0.03
Creativity	−0.13	−0.22	−0.05	−0.09	−0.16	−0.02
**ANTECEDENTS OF DETACHMENT**						
**Job Demands**						
Combined	−0.26	−0.30	−0.22	−0.25	−0.28	−0.21
Quantitative demands	−0.28	−0.33	−0.24	−0.27	−0.31	−0.22
Social conflicts	−0.29	−0.39	−0.18	−0.22	−0.33	−0.10
Emotional demands	−0.25	−0.29	−0.21	−0.20	−0.28	−0.12
Working time	−0.17	−0.22	−0.12	−0.15	−0.20	−0.11
Role stressors	−0.14	−0.20	−0.08	−0.12	−0.17	−0.06
**Job Resources**						
Combined	0.10	0.03	0.16	0.11	0.05	0.18
Social support	0.17	0.10	0.25	0.23	0.16	0.30
Job control	0.05	0.01	0.09	0.07	0.02	0.11
**Work-related Activities**	−0.32	−0.38	−0.26	−0.29	−0.36	−0.22
**Person Characteristics**						
Age	−0.03	−0.06	0.00	−0.02	−0.05	0.02
Gender	0.03	−0.01	0.07	0.04	0.01	0.08
Negative affectivity/neuroticism	−0.24	−0.37	−0.10	−0.18	−0.30	−0.07
Heavy work investment	−0.36	−0.46	−0.25	−0.29	−0.32	−0.25

*$\overset{-}{r}$, sample size weighted mean correlation; CI, confidence interval of $\overset{-}{r}$; LL, lower limit of the 95% CI; UL, upper limit of the 95% CI*.

### Publication bias

As the accuracy of meta-analytic results might be impacted by a potential publication bias (Borenstein et al., 2009), we examined the results using three techniques: visual inspection of funnel plots (see Supplementary Material), Egger's regression test, and Duval and Tweedie's (2000) trim and fill method (Borenstein et al., 2009; see Table 5 for the latter tests).

**Table 5:** **Summary of results for statistical tests to detect a publication bias**.

	**Egger Test**			**Trim and Fill**				
	***b***	***SE***	***t***	***n***	**Side**	**$\overset{-}{r}$**	***LL***	***UL***
**HEALTH**								
Exhaustion	1.17	1.41	0.83	0				
Life satisfaction^a^	7.98	2.33	3.43^*^	0				
Well-being	1.02	0.97	1.05	0				
Sleep	−3.03	1.39	2.18^*^	4	Right	0.35	0.28	0.43
Physical discomfort^a^	−4.10	1.91	2.15	1	Left	−0.26	−0.35	−0.18
Burnout (others)^a^	5.82	3.18	1.83	0				
Physiological activation^a^	1.83	4.70	0.39	0				
**STATE WELL-BEING**								
Fatigue	3.74	2.54	1.47	2	Left	−0.45	−0.55	−0.35
Affect	−0.21	0.89	0.23	2	Left	0.25	0.18	0.32
State recovery^a^	1.72	2.19	0.79	0				
**MOTIVATION**								
Work motivation	0.71	1.75	0.41	0				
**WORK PERFORMANCE**								
Task performance^a^	−0.41	0.81	0.50	0				
Contextual performance^a^	−1.23	1.66	0.74	0				
Creativity^a^	−0.55	2.24	0.24	0				
**WORK CHARACTERISTICS**								
Job demands	0.61	0.68	0.88	0				
Quantitative demands	0.26	0.80	0.32	0				
Social conflicts	3.86	2.37	1.63	0				
Emotinal demands^a^	1.63	1.57	1.04	1	Right	−0.21	−0.27	−0.14
Working time	−0.67	0.84	0.79	2	Left	−0.18	−0.22	−0.13
Role stressors^a^	−2.19	0.89	2.46^*^	2	Right	−0.11	−0.16	−0.06
Job resources	−1.85	1.13	1.64	1	Right	0.11	0.05	0.17
Social support^a^	−0.08	2.36	0.03	0				
Job control	0.30	0.70	0.42	0				
Work-related activities	−0.48	1.09	0.45	0				
**PERSON CHARACTERISTICS**								
Age	−1.01	0.48	2.11^*^	0				
Gender (Females)	−0.72	0.58	1.25	5	Left	0.01	−0.03	0.05
Negative affectivity/neuroticism	5.04	2.28	2.21^*^	4	Left	−0.28	−0.39	−0.16
Heavy work investment^a^	−1.70	1.10	1.55	0				

*b, unstandardized regression weight; SE, standard error; t, t-value with df, k – 2; n, number of imputed effect sizes; $\overset{-}{r}$, sample size weighted mean correlation after effect size imputation; LL, lower limit of the 95% CI of $\overset{-}{r}$; UL, upper limit of the 95% CI of $\overset{-}{r}$*;

* *p < 0.05*.

a *Results of Egger test for funnel plot asymmetry should not be interpreted as total k < 10 (Sterne et al., 2011)*.

Regarding the funnel plots, to our interpretation (we only interpret funnel plots based on *k*s > 9; Sterne et al., 2011), an asymmetrical distribution of effect sizes and their standard errors appeared for the variables sleep, fatigue, and negative affectivity/neuroticism. For sleep and negative affectivity/neuroticism this was also underlined by significant results of a statistical test procedure (Egger's test; Table 5). Moreover, results of Egger's test revealed an asymmetric effect size distribution for age. However, it has to be noted that funnel plot asymmetry might also arise from other sources than publication bias, for instance, high heterogeneity of effect sizes as in our sample of studies (Sterne et al., 2011). Thus, we stratified funnel plot analysis for the variables sleep, fatigue, and negative affectivity/neuroticism by the subgroups of two significant moderators: study design and affective valence of work-related thoughts (plots are not shown here). Funnel plot asymmetry disappeared for fatigue in these subgroups. We found no longer asymmetrical funnel plots for sleep and negative affectivity/neuroticism when analyzing them separately under the subgroups of study design. However, for both variables funnel plot asymmetry was still present under the condition that studies assessed detachment as absence of negative work-related thoughts during non-work time. Note that under this condition heterogeneity of effect sizes was high and likewise might have caused these results.

In the next step, we applied the trim-and-fill-method to estimate how average effect sizes would change if potentially missing study effect sizes, necessary for funnel plot symmetry, are included in the analysis (see Table 5). Results revealed that average effect size estimates only marginally changed and the direction and significance of effects remained stable.

To sum up, we found only minor evidence for a potential publication bias, most likely present for relationships between detachment and sleep and negative affectivity/neuroticism. However, these results might have been also caused by heterogeneity of effect sizes. Results of the trim-and-fill method showed that a potential publication bias is not a serious threat to the validity of most of the presented average effects.

## Discussion

After a period of work, recovery is required to replenish drained mental and physical resources (Meijman and Mulder, 1998). In the last years, detachment from work has been proposed as an important psychological recovery process that might explain how effects of work characteristics translate into employees' state well-being, physical and mental health, work motivation, and work performance (Sonnentag, 2012; Sonnentag and Fritz, 2015). The objective of the present meta-analysis was to examine the direction and strength of associations between theoretically proposed antecedents and outcomes of daily detachment from work and to investigate effects of potential moderator variables. Below we discuss our findings.

### Outcomes and antecedents of detachment

#### Outcomes

In line with the SDM (Sonnentag and Fritz, 2015), we found that detachment from work is positively related to indicators of self-reported mental health and state well-being. Detachment was, on average, most strongly related to employees' fatigue, exhaustion, short- and long-term well-being, and sleep. Moreover, we found an average positive relationship between detachment and self-reported state of recovery. These results support the assumption that detachment from work is a strong indicator of psychological recovery from work during non-work time (Sonnentag and Fritz, 2015).

Notably, we found some preliminary evidence, albeit for a small sample of studies, that detachment from work might also be beneficial for recovery from work-related physical strain. Whereas, detachment at its core is a psychological construct, it is, by definition, also linked with a relief from physical job demands, which might explain these results.

Detachment from stress-related thoughts might also prevent prolonged physiological stress reactions (Brosschot et al., 2005, 2006). Results of a recent meta-analysis supported this assumption (Ottaviani et al., 2016). In contrast to this review, we used a more narrow focus concerning the conceptualization of detachment, the study samples, and the study context. Thus, we only found three studies that examined this hypothesis in a work context with employee samples. In our study, detachment did, on average, not significantly correlate with physiological stress indicators. However, the types of assessed indicators differed between the studies and might represent different physiological subsystems. This might explain dispersion of effect sizes in the primary studies. Moreover, desynchronization of physiological activation due to low detachment might be a more long-lasting process that needs to be studied over longer periods than covered here (see also results of Ottaviani et al., 2016).

In contrast to our assumptions, we found, on average, no significant linear relationship between detachment and work motivation. However, only recently Shimazu et al. (2016) showed that this relationship might be more complex. Accordingly, they found a curvilinear relationship between detachment and work engagement. Thus, work engagement was lowest under conditions of low and high detachment and highest at mean levels of detachment. While low detachment from work means that recovery is impaired as resources are further drained even after work, situations of high detachment might turn into motivational problems as employees need longer to get back into “working mode” (Shimazu et al., 2016).

Similarly, we found unexpected patterns concerning the average relationships between detachment and work performance. As expected, detachment from work positively correlated with task performance. However, the effect size was small. Again, at least one study found an inverted u-shaped pattern between detachment and task performance (Fritz et al., 2010b). This might put our assumptions of linear relationships somewhat into question. Unexpectedly, we found that detachment significantly *negatively* correlated with measures of contextual work performance and creativity, which comprises activities that go beyond stipulated tasks as helping others at work or innovating behavior (Sonnentag et al., 2008c). In other words, persons that mentally engaged in work-related thoughts even during non-work time showed higher contextual performance and creativity. One might speculate about the reason for this relationship as well as about the causal mechanisms. On the one hand, contextual performance and creativity might involve additional work-related activities during non-work time, which hinders detachment from work. On the other hand, the valence of work-related thoughts might be important. For example, Binnewies et al. (2009) found that only positive but not negative work reflection positively predicted contextual performance and creativity 6 months later. Thus, low detachment might not always be detrimental for all types of work performance. In our study, it was not possible to disentangle the role of affective valence for the relationship between detachment and work performance measures due to a limited sample of studies. In conclusion, future studies should investigate motivational and performance-related outcomes of detachment with more fine-grained approaches. This concerns the expected patterns of relationships, i.e., also non-linear ones, the use of more objective indicators for different types of work performance, and the content of work-related thoughts during non-work time.

An important caveat on the interpretation of the *average* relationships discussed above regards the dispersion of effect sizes in the primary studies. Heterogeneity of effect sizes was mainly moderate to high (except for physiological activation as outcome) and only for exhaustion and well-being as outcomes the chance that future studies find effects in similar direction was high. Thus, mean effects should be interpreted with caution and also in the context of further moderating variables. We discuss the impact of moderators later in this paper.

#### Antecedents

In our meta-analysis, we examined three groups of variables as antecedents of detachment from work: work characteristics, work-related activities during non-work time, and individual variables.

First, we found that, on average, job demands negatively and job resources positively correlate with detachment from work. Mean effects were small sized. Our results support the assumption of the SDM (Sonnentag and Fritz, 2015) that job stressors impair detachment from work. Moreover, we found that qualitatively different types of job demands (i.e., quantitative work demands, social stressors, emotional demands, working time, and role conflicts) all significantly negatively correlated with detachment from work. However, further studies are necessary to confirm such relationships, including types of job demands, which to date are less well-researched in this field (e.g., shift work, job uncertainty, and illegitimate tasks). Furthermore, the pattern of our results supports prior assumptions that both cognitive (e.g., goal-discrepancies in cases of high quantitative demands) and emotional (e.g., negative affect in cases of social conflicts and emotional demands) processes might mediate these relationships. However, to our knowledge, there is still a gap concerning the causal mechanisms linking job demands and detachment. Thus, we encourage scholars to investigate this issue more systematically. Furthermore, Cavanaugh et al. (2000) have recently suggested considering appraisal processes when investigating strain effects of job demands. In line with these assumptions, two meta-analyses found higher positive correlations for hindrance (i.e., negative appraisal) than challenge (i.e., positive appraisal) stressors to strain outcomes (LePine et al., 2005; Podsakoff et al., 2007). Moreover, challenge stressors had a positive relationship and hindrance stressors a negative relationship to work motivation and work performance (Podsakoff et al., 2007). Thus, it might be promising to investigate the relationship between job demands and detachment according to stressor type. Our data suggests higher negative correlations for challenge (i.e., quantitative demands) than hindrance stressors (i.e., role stressors) to detachment from work which is in line with results of a recent cross-sectional study as well (Siu, 2013). However, affective valence of work-related thoughts might be a further moderating factor in this context. Thus, it would be interesting to see whether challenge stressors induce more positive work reflection and hindrance stressors induce more negative work-reflection, which might have different effects for recovery.

It is worth noting that we found, on average, a significant positive but small correlation between job resources and detachment. Kinnunen et al. (2011) recently suggested such a relationship according to their Job Demands-Resources-Recovery-Model. However, our results showed that the strength of this association is weaker than for relationships between job demands and detachment. Thus, increasing job resources might only have minor direct effects on detachment. Future studies should examine the mutual and interactive impact of job demands and job resources on detachment to better understand these linkages. On the one hand, job resources promote goal achievement (Bakker and Demerouti, 2007) which should prevent goal-discrepancies (Smit, 2016) and, in turn, a lack of detachment during non-work time. On the other hand, the relationship between job resources and detachment might be spurious and triggered by the often found small but negative relationship between job resources and job demands (for meta-analytic findings see Crawford et al., 2010; Nahrgang et al., 2011). According to the Job-Demands-Resources Model (Bakker and Demerouti, 2007) there might also be more complex interaction effects. Thus, as stated by Sonnentag and Fritz (2015), is possible that job resources moderate the relationship between job demands and detachment in a way that negative effects of job demands on detachment will diminish when job resources increase.

A second finding was the average negative correlation between work-related activities during non-work time and detachment. Thus, the continued presence of work-related stressors during non-work time hampers mental disengagement from work. Noteworthy, the size of effect was similar to that for job demands. Intuitively, such a correlation might not be surprising, as engagement in work-related activities during non-work time increases the *mental and physical presence* of work during non-work time. However, beyond such possible artificial methodological variance, there is also some empirical evidence that engagement in work-related activities during non-work time is translated into higher strain and lower well-being (Sonnentag, 2001; Sonnentag and Natter, 2004; Sonnentag and Zijlstra, 2006) because of lower detachment from work (ten Brummelhuis and Bakker, 2012). Accordingly, our results suggest controlling for work-related activities during non-work time in future studies when investigating other possible antecedents and outcomes of detachment.

Finally, we examined the anteceding role of person characteristics for detachment from work. We found that, on average, detachment is unrelated to age and gender. A recent meta-analysis found a significant small and positive correlation between age and cognitive irritation (*r* = 0.10; Rauschenbach et al., 2013). As noted above, cognitive irritation has some conceptual overlaps to the detachment concept but reflects more general and long-term problems of mental disengagement from work (Mohr et al., 2006). However, results of both meta-analyses illustrate that direct effects of employees' age on detachment are rather small or negligible. This also concerns direct effects of gender. Prior studies found that females have more difficulties to physiologically unwind after work than males (Frankenhaeuser et al., 1989; Lundberg and Frankenhaeuser, 1999). One possible explanation for this finding is that females often take on greater responsibilities for household and childcare tasks during leisure time than males (Sonnentag and Bayer, 2005; Mojza et al., 2011; ten Brummelhuis and Bakker, 2012; Volman et al., 2013). However, concerning detachment from work such gender differences seem to be negligible.

We further examined the anteceding role of negative affectivity/neuroticism and heavy work investment. Both variables significantly negatively correlated with detachment from work. Recent meta-analyses found that people with higher values on these variables report more severe experiences of negative emotions (DeNeve and Cooper, 1998; Clark et al., 2016) and work stressors (Bowling et al., 2015; Clark et al., 2016). Thus, our results underline the assumption of Sonnentag and Fritz (2015) that person characteristics related to appraisal processes of the work situation might further influence detachment. Hence, future research should control for these factors when analyzing other antecedents and outcomes of detachment. However, we should not forget that average correlations between detachment and both individual difference variables were only small to moderate (about 5 to 10% explained variance in detachment). Thus, it is unlikely that detachment only reflects these individual differences. Furthermore, several studies found the expected relationships between higher detachment and lower demands (Mojza et al., 2010; Sonnentag et al., 2010a), lower strain and higher well-being (Sonnentag et al., 2008a, 2010a; Fritz et al., 2010b; Donahue et al., 2012; Querstret and Cropley, 2012; Sonnentag and Binnewies, 2013; Wang et al., 2013) even after controlling for negative affectivity/neuroticism and heavy work investment. Thus, such a common variance between detachment and both individual differences variables does not restrict the expected relationships to work-related antecedents and outcomes of detachment according to the SDM (Sonnentag and Fritz, 2015).

Again, we note that all average effects discussed above should be interpreted with caution as heterogeneity of effect sizes was at least moderate to high. Moreover, the range of prediction intervals reveals that similar directed effects can be expected only for quantitative demands, emotional demands, engagement in work-related activities during non-work time, and heavy work investment.

#### Moderator variables

With one exception (the relationships between detachment and physiological stress indicators), we found for all analyzed relationships between detachment and its antecedents and outcomes a substantial moderate to high heterogeneity and dispersion of effect sizes. Thus, we examined several potential moderator variables to account for these variances. As noted above, multiple comparisons in moderator analyses problematically increase type I error rates (Cafri et al., 2010). Therefore, significant findings of these analyses should be interpreted with caution and as stimulating perspective for further research.

First, most studies in this field were conducted with European samples. Thus, a reliable comparison of effect sizes between different countries, which might uncover further cultural influences, was not possible for various variables (especially for outcomes of detachment). However, for several associations between detachment and work-related (job demands, quantitative demands, working time, job resources) and individual (age, gender, negative affectivity/neuroticism) variables we found no moderating impact of study location. Therefore, for most relationships the reported average effect sizes were rather robust in European and North American samples.

Second, study design moderated the associations between detachment and sleep, fatigue, and negative affectivity/neuroticism. This moderator variable explained between nine (fatigue) and 19% (sleep) between-study variance in effect sizes. More specifically, average between-person correlations with detachment were lower and less heterogeneous in diary studies than in cross-sectional studies with one measurement occasion. This finding might be explained by the more reliable measurement of variables in diary studies due to repeated measurement and a reduction of retrospective bias (Ohly et al., 2010). Moreover, the average negative association between negative affectivity/neuroticism and detachment was no longer significant in diary studies (and also in three longitudinal studies). This suggests that a potential bias of correlations to detachment due to further influences of negative affectivity/neuroticism (Spector et al., 2000) might be less problematic in studies with repeated measures (i.e., experience sampling and longitudinal designs).

Third, regarding the moderating impact of demographic study differences, we found no impact of mean age in our sample of studies. However, examining age as moderator for relationships between detachment and its work-related antecedents and strain-related outcomes might be a promising avenue for future research as indicated by a couple of findings from occupational health psychology (Zacher and Schmitt, 2016). We found two interesting results concerning the moderating impact of samples' gender distribution. More specifically, the correlations between detachment and work motivation shifted toward positively directed effects whereas the negative correlations between negative affectivity/neuroticism and detachment decreased with an increasing percentage of females in the samples. Samples' gender distribution explained more than half of the between-study variance for both relationships. To our knowledge, gender-moderated effects of detachment have not been examined yet. We think that these effects are difficult to explain. For instance, with regard to the gender-moderated relationship between detachment and work motivation the extended stressor-detachment model (Sonnentag and Fritz, 2015) suggests that for persons with high proactive and problem-focused coping the relationships between detachment and strain outcomes will be reduced or annihilated. The meta-analysis of Tamres et al. (2002) revealed that females engage in both coping styles more than males. However, this would suggest an opposite pattern of results and, moreover, in our data we found no support that women would neither reflect more positive nor more negative about work during non-work time than men. Moreover, regarding the second effect, gender-moderated relationships between negative affectivity/neuroticism and detachment, it might be that females' higher engagement in coping (see Tamres et al., 2002) mitigates adverse influences of negative affectivity/neuroticism for detachment. However, as the detected moderator effects of gender base on between-study differences and not on within-study relationships, we suggest that future research should develop and examine more sophisticated hypotheses to explain these effects.

Fourth, we found a moderating impact of valence of work-related thoughts during non-work time for several relationships of detachment (i.e., sleep, affect, demands, age, and negative affectivity/neuroticism). Explained between-study variance of this moderating variable varied between six (affect) to 49% (negative affectivity/neuroticism). It is important to note that our analyses were restricted to a limited number of relationships to detachment as well as subgroups of valence (neutral vs. negative). We found that low detachment has, on average, more severe negative consequences for sleep and affect when employees cannot detach *from negative work-related thoughts*. This pattern also emerged for fatigue as outcome. However, the moderator effect was not significant here. These results support the assumptions of Cropley and Zijlstra (2011) and Sonnentag and Fritz (2015) that the content of work-related thoughts is important when predicting outcomes of detachment. More specifically, low detachment might become more problematic if work-related coping and reappraisal processes during recovery periods are linked with negative affective states that are translated into sustained physiological and cognitive activation and, in turn, impaired recovery and prolonged and accumulated strain (see also Brosschot et al., 2005; Meurs and Perrewé, 2011). In contrast, positive work reflection might be not detrimental for affect. Furthermore, we found that valence of work-related thoughts also moderated relationships between specific anteceding variables and detachment. For instance, average negative associations of overall job demands and negative affectivity/neuroticism to detachment were strongest when assessing detachment as absence of negative work-related thoughts. For job demands as antecedent this pattern is in line with assumptions from the extended stressor-detachment model (Sonnentag and Fritz, 2015). Building upon transactional stress theory this model states that low detachment will be more likely if employees evaluate job demands as threatening or harmful, thus, affectively negative. For negative affectivity/neuroticism the moderator effect of valence might be explained by a better match of measures. As persons rating high in negative affectivity and neuroticism are more sensitive to experience negative emotions across time and situations (Watson et al., 1988; Costa and McCrae, 1992) they should report poorer detachment when they are specifically asked for *affectively negative* work-related thoughts than for global ones. Albeit there was a moderator effect of valence for the relationship between age and detachment, mean subgroup correlations remained negligible.

In sum, our results suggest a strong need that future studies focus on potential moderating variables affecting relationships to detachment. As uncovered in this study and also in line with recent theoretical developments (Sonnentag and Fritz, 2015), the valence and content of work-related thoughts during non-work time (e.g., Binnewies et al., 2009; Cropley et al., 2012) seems to be a promising factor. Thus, we encourage scholars to assess detachment from work with more fine-grained approaches and also in combination with further impacting variables, i.e., appraisal and coping processes and combinations of work-related and person-related antecedents. This is important to better understand the conditions under which high detachment will develop and, in turn, impact health, well-being, work motivation, and work performance.

### Limitations and future research directions

Our study is not without limitations that should be considered when interpreting the results. First, we only included English written studies published in scientific journals. Therefore, pooled effect size estimates and heterogeneity of effect sizes might change when including Non-English and unpublished studies. However, we found only weak evidence for a possible publication bias (Borenstein et al., 2009) and simulation analyses revealed only a minor impact of such a bias for the presented average effects. Thus, the reported pooled effect size estimates seem to be relatively representative and robust. This is also underlined by the results of the presented sensitivity analysis. Nevertheless, future meta-analyses might extend the scope of literature search. This concerns not only the type of included publications but also the role of detachment within other recovery periods (e.g., breaks, weekend, and vacation) than covered in the present study.

Second, our results are primarily based on individual-level and self-reported data (some exceptions are correlations to physiological data and sleep). Thus, correlations might be inflated by a common-method bias (Podsakoff et al., 2012). However, at least some of the included studies assessed predictors and dependent variables at different moments in time or used repeated cross-sectional assessments (diary studies) which both mitigate this problem. So far, longitudinal data is rare and should be assessed more often in future studies. At least for well-being and exhaustion as outcomes and job demands, quantitative demands, and working time as antecedent of detachment, we found similar average effects in a couple of longitudinal studies. Moreover, to our knowledge, experimental studies concerning antecedents and effects on detachment are scarce. While we found only two intervention studies investigating effects of manipulated work reflection during non-work time (Bono et al., 2013; Meier et al., 2016), there is a severe gap concerning the impact of work design interventions on detachment. Without any doubt, research on detachment from work would benefit from studies assessing objective and multi-source data, both for antecedents and outcomes, to further validate the core assumptions of the (extended) SDM.

Third, some of the work-related antecedents and outcomes of detachment are less well-examined. Thus, our combined measures of job demands and resources are at least to some extend biased by the higher proportion of frequently measured variables (e.g., quantitative demands, social conflicts, working time, job control). We tried to mitigate this problem by reporting separate correlations for meaningful groups of constructs. This was successful, given first insights for differential effect sizes. Nevertheless, future research should focus more frequently on constructs less investigated.

Another caveat might be that our results are biased by interdependence of constructs and measures stemming from one study which were treated as independent here. For instance, the meta-analysis of Clark et al. (2016) revealed workaholism to be significantly related to negative affectivity, job demands, job resources, and exhaustion. In our analysis, we only controlled for dependence of variables measuring the same construct (e.g., studies reporting separate correlations between detachment and different aspects of work engagement: vigor, dedication, and absorption). Future research might use meta-analytical multilevel structural equation modeling (Cheung, 2015) to face the problem of dependent effect size in a more sophisticated way.

Finally, future studies should concentrate more intensively on potentially moderating variables. Even after controlling for several study-related individual and conceptual moderator variables a substantial amount of variance in effect sizes remained unexplained for most of the examined relationships to detachment. However, we note that for some of these analyses sample sizes (also of subgroups) were small, limiting the statistical power of meta-analytical moderation analysis. We think that it is most likely to uncover this heterogeneity of effect sizes by investigating a more comprehensive and extended version of the SDM as recently suggested by Sonnentag and Fritz (2015). In addition, as indicated by our results, analyses should be adjusted for some of the most important confounding variables of detachment (i.e., negative affectivity/neuroticism, heavy work investment, and time for work-related activities during non-work time).

### Practical implications

Keeping in mind that detachment from work positively relates to employees' health, well-being, and performance, employees and organizations should seek successful interventions to improve detachment from work. Our results point to two general approaches.

The first strategy calls for *organizational-level interventions* (DeFrank and Cooper, 1987; Sonnentag and Frese, 2012). According to the results above, this would mean to minimize high quantitative demands (e.g., by adequate staffing), emotional demands and social conflicts (e.g., by organizational routines to prevent customer- and co-worker-conflicts), role stressors (e.g., by clear rules and structures about how to satisfy expectations for a single role), and an extension of working time above upper threshold values according to national regulation. Moreover, our data also suggests that promoting job resources as social support (e.g., by co-worker, supervisors, or the organization) and job control (e.g., timing control, scheduling control, control over supplies, and environmental control; Carayon and Zijlstra, 1999) might be helpful. So far, to our knowledge, there has been no intervention study examining effects of these job factors on detachment. Investigating a different job factor, Coffeng et al. (2014) probed a physical environment intervention in a sample of office workers. The intervention group's work environment was rearranged to stimulate different recovery behaviors and experiences (e.g., socializing by tables and chairs, plants, relaxing wall posters). However, detachment at work and after work did not significantly change in contrast to a control group after 6 and 12 months. This might suggest that it is more important to change task and organizational factors than workplace physical environment when aiming for an improved ability to detach from work.

Organizations should also promote boundary management by developing and communicating a clear and transparent policy regarding their expectations on availability and working during non-work time. This is important as new technological (e.g., smartphones and e-mail) and organizational (e.g., home-office) developments have increased availability of employees and work during non-work time. On the one hand, employees might enhance work-life balance by working outside the office. One the other hand, there is also some evidence that high quantitative demands (Derks et al., 2014b), low autonomy and low job control (Richardson and Thompson, 2012; Derks et al., 2014a), and longer working hours (Ohly and Latour, 2014) lead employees to spent more time with work-related activities during non-work time. Thus, organizations' boundary management activities should be based on healthy work-design to improve employees' recovery.

The second strategy focusses on *individual-level, person-centered interventions* (Sonnentag and Frese, 2012). Examples are stress management programs (e.g., relaxation and cognitive-behavioral techniques, sleep training), trainings for competence and skill enhancement, and lifestyle change programs (e.g., exercise programs, well-being interventions). So far, several studies found positive effects of such interventions to improve detachment from work. For instance, Bono et al. (2013) revealed that daily positive work reflection improves detachment from work. Moreover, more complex recovery trainings showed small (Michel et al., 2014; Siu et al., 2014; Querstret et al., 2016) to large (Hahn et al., 2011; Thiart et al., 2015) positive effects on detachment from work. In addition to guided trainings, employees might also improve detachment by using powerful recovery activities during non-work time. More specifically, this concerns the distractive nature of social activities (Mojza et al., 2010, 2011; ten Brummelhuis and Bakker, 2012; Cropley et al., 2015) and physical activities (Sonnentag and Bayer, 2005; Mojza et al., 2010; ten Brummelhuis and Bakker, 2012; Cropley et al., 2015).

## Conclusions

Balancing work and rest is, intuitively, necessary to live a healthy, happy, and productive life, and is, thus, a problem as old as humankind (Hockey, 2013). Hence, from a scholarly perspective, it is challenging to identify variables that shed light on this interplay. However, even in the last years, scholars identified detachment from work as one important psychological recovery process variable. Therefore, we quantitatively reviewed theoretically proposed outcomes and antecedents of detachment from work.

On a fundamental level, we found that detachment from work positively relates to mental and physical health, state well-being, and task performance. Moreover, improved work characteristics (i.e., lower job demands and higher job resources), less work-related activities during non-work time, and individual difference variables, such as a lower sensitivity to experience negative emotions or a working style characterized by less extensive work engagement, positively antecede detachment. However, our results reveal that the functional ties of detachment from work are probably even more complex. For example, we found that affective valence of work-related thoughts during non-work time might be a promising moderator variable that needs more attention.

We hope that this study stimulates future research to gain more insights into factors and processes of successful psychological recovery in the work-rest cycle.

## Author contributions

JW and AL-H designed the study. JW and AL-H reviewed the articles. JW analyzed the data. JW wrote the first draft of the paper. Both authors discussed the results. AL-H commented on the manuscript.

## Funding

This study was part of the project “Mental health in the working world—current state of scientific evidence” (F 2353) granted by the German Federal Institute for Occupational Safety and Health.

### Conflict of interest statement

The authors declare that the research was conducted in the absence of any commercial or financial relationships that could be construed as a potential conflict of interest.
