# Supplementary material for: A Meta-Analysis on Antecedents and Outcomes of Detachment from Work
Source: Front Psychol. 2017 Jan 13;7:2072. doi: 10.3389/fpsyg.2016.02072 (PMC5233687; doi:10.3389/fpsyg.2016.02072)
Supplement: Supplementary file 1 [file DataSheet1.PDF]

Supplementary Material

A Meta-Analysis on Antecedents and Outcomes of Detachment from Work

Johannes Wendsche[1,2*], Andrea Lohmann-Haislah[2]

[1]Federal Institute for Occupational Safety and Health, Regional Transfer/Special Sectors, Dresden, Germany

[2]Federal Institutefor Occupational Safety and Health, Mental Workload, Berlin, Germany

1 Supplementary Figures: Forest Plots

Study name	Variable	Detachment	Correlation	Lower limit	Upper limit	Z-Value	p-Value
Sonnentag & Fritz (2007)	Exhaustion	Composite	-0,560	-0,665	-0,433	-7,326	0,000
Rivkin et al. (2014)_Study 1	Exhaustion	Composite	-0,540	-0,603	-0,471	-12,702	0,000
Rivkin et al. (2014)_Study 2	Exhaustion	Composite	-0,510	-0,577	-0,436	-11,574	0,000
Querstret et al. (2015)	Exhaustion	Composite	-0,510	-0,600	-0,407	-8,422	0,000
Querstedt & Cropley (2012)	Exhaustion	Composite	-0,502	-0,554	-0,445	-14,757	0,000
Sonnentag et al. (2010b)	Exhaustion	Composite	-0,500	-0,616	-0,362	-6,335	0,000
Hernández et al. (2013)	Exhaustion	Composite	-0,470	-0,607	-0,306	-5,151	0,000
Donahue et al. (2012)	Exhaustion	Composite	-0,440	-0,575	-0,282	-5,064	0,000
Danner-Vlaardingerbroek et al. (201[illegible])	Exhaustion	Composite	-0,420	-0,482	-0,353	-11,174	0,000
Derks et al. (2014b)	Exhaustion	Composite	-0,400	-0,580	-0,182	-3,468	0,001
Fritz et al. (2010)	Exhaustion	Composite	-0,380	-0,532	-0,205	-4,080	0,000
Kinnunen et al. (2015)	Exhaustion	Composite	-0,350	-0,401	-0,297	-12,137	0,000
Sonnentag et al. (2010a)	Exhaustion	Composite	-0,340	-0,435	-0,237	-6,194	0,000
Allen et al. (2005)	Exhaustion	Composite	-0,340	-0,405	-0,271	-9,159	0,000
de Jonge et al. (2012)	Exhaustion	Composite	-0,300	-0,387	-0,208	-6,162	0,000
Demerouti et al. (2012)	Exhaustion	Composite	-0,275	-0,463	-0,063	-2,524	0,012
Els et al. (2015)Mostert & Els (2015)	Exhaustion	Composite	-0,270	-0,362	-0,172	-5,275	0,000
Kinunnen & Feldt (2013)	Exhaustion	Composite	-0,260	-0,370	-0,143	-4,283	0,000
Hahn et al. (2011)	Exhaustion	Composite	-0,220	-0,407	-0,016	-2,110	0,035
Derks & Bakker (2014)	Exhaustion	Composite	-0,190	-0,408	0,049	-1,563	0,118
Sonnentag et al. (2014)	Exhaustion	Composite	-0,190	-0,365	-0,002	-1,980	0,048
Virga & Paveloni (2015)	Exhaustion	Composite	-0,040	-0,217	0,139	-0,435	0,664
Barber & Santuzzi (2015)	Exhaustion	Composite	-0,010	-0,123	0,103	-0,173	0,862
			-0,361	-0,418	-0,302	-11,195	0,000

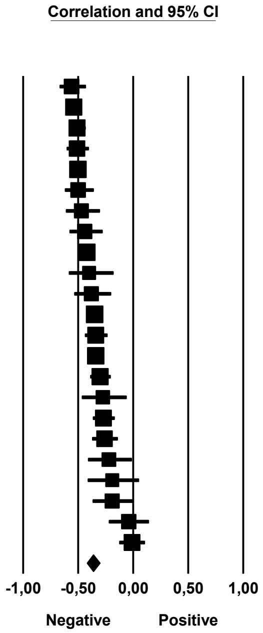

Supplementary Figure 1a. Forest plot for burnout (exhaustion).

Study name	Variable	Detachment	Correlation	Lower limit	Upper limit	Z-Value	p-Value
Park & Fritz (2014)	Life Satisfaction	Composite	0,050	-0,028	0,127	1,259	0,208
Hahn & Dormann (2013)	Life Satisfaction	Composite	0,230	0,103	0,349	3,513	0,000
Fritz et al. (2010)	Life Satisfaction	Composite	0,330	0,150	0,489	3,496	0,000
Sonnentag & Fritz (2007)	Life Satisfaction	Composite	0,370	0,216	0,506	4,496	0,000
Moreno-Jiménez et al. (2009a)	Life Satisfaction	Composite	0,590	0,464	0,693	7,577	0,000
			0,320	0,112	0,500	2,969	0,003

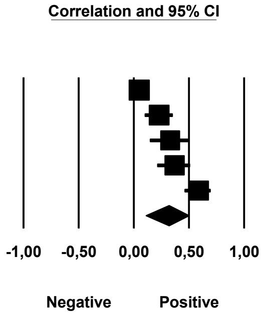

Supplementary Figure 1b. Forest plot for life satisfaction.

Study name	Variable	Detachment	Correlation	Lower limit	Upper limit	Z-Value	p-Value
			Statistics for each study				
Moreno-Jiménez et al. (2009b)	Well-being	Composite	0,120	0,034	0,205	2,718	0,007
Volman et al. (2013)	Well-being	Composite	0,170	-0,077	0,397	1,352	0,176
Shimazu et al. (2012)	Well-being	Composite	0,180	0,142	0,218	9,130	0,000
Richardson & Thompson (2012)	Well-being	Composite	0,230	0,066	0,382	2,731	0,006
Sonnentag et al. (2013)	Well-being	Composite	0,250	0,139	0,355	4,334	0,000
Sonnentag et al. (2010a)	Well-being	Composite	0,270	0,163	0,370	4,843	0,000
Els et al. (2015)Mostert & Els (2015)	Well-being	Composite	0,272	0,174	0,364	5,315	0,000
Hahn et al. (2011)	Well-being	Composite	0,300	0,105	0,473	2,969	0,003
Kompier et al. (2012)	Well-being	Composite	0,310	0,285	0,334	23,126	0,000
Moreno-Jiménez et al. (2012)	Well-being	Composite	0,340	0,284	0,394	11,124	0,000
Ward et al. (2013)	Well-being	Composite	0,340	0,143	0,511	3,303	0,001
Moreno-Jiménez et al. (2009a)	Well-being	Composite	0,360	0,199	0,502	4,214	0,000
White (2011)	Well-being	Composite	0,440	0,178	0,644	3,168	0,002
Sonnentag & Fritz (2007)	Well-being	Composite	0,476	0,335	0,596	5,995	0,000
Nicholson & Griffin (2014)	Well-being	Composite	0,510	0,391	0,612	7,380	0,000
Bono et al. (2013)	Well-being	Composite	0,713	0,562	0,818	6,800	0,000
			0,317	0,260	0,372	10,364	0,000

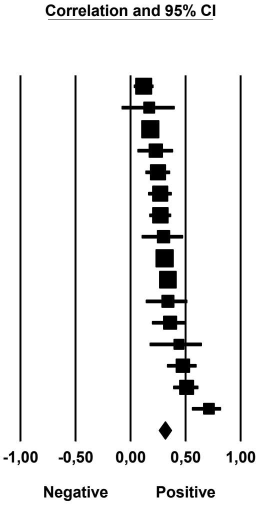

Supplementary Figure 1c. Forest plot for well-being.

Study name	Variable	Detachment	Correlation	Lower limit	Upper limit	Z-Value	p-Value
			Statistics for each study				
Niks et al. (2015)	Sleep	Composite	-0,018	-0,257	0,223	-0,141	0,888
Pereira et al. (2014)	Sleep	Composite	0,017	-0,209	0,242	0,149	0,882
Hülsheger et al. (2015)	Sleep	Composite	0,103	-0,064	0,265	1,213	0,225
Sonnentag & Binnewies (2013)	Sleep	Composite	0,170	-0,032	0,358	1,655	0,098
Vahle-Hinz et al. (2014)	Sleep	Composite	0,180	-0,104	0,436	1,248	0,212
Barber & Santuzzi (2015)	Sleep	Composite	0,192	0,081	0,299	3,373	0,001
Sonnentag et al. (2008a)	Sleep	Composite	0,221	0,071	0,361	2,865	0,004
Sonnentag & Fritz (2007)	Sleep	Composite	0,250	0,086	0,401	2,957	0,003
Barber & Jenkins (2014)	Sleep	Composite	0,266	0,160	0,366	4,815	0,000
Cropley et al. (2006)	Sleep	Composite	0,279	0,120	0,424	3,392	0,001
Querstret et al. (2015)	Sleep	Composite	0,290	0,166	0,405	4,469	0,000
Querstedt & Cropley (2012)	Sleep	Composite	0,312	0,244	0,376	8,625	0,000
Hülsheger et al. (2014)	Sleep	Composite	0,340	0,169	0,491	3,781	0,000
Kompier et al. (2012)	Sleep	Composite	0,380	0,357	0,403	28,863	0,000
Cropley et al. (2013)	Sleep	Composite	0,454	0,290	0,592	5,021	0,000
Hahn et al. (2011)	Sleep	Composite	0,470	0,289	0,619	4,703	0,000
Rodríguez-Muñoz et al. (2011)_Sample2	Sleep	Composite	0,510	0,477	0,541	25,354	0,000
Rodríguez-Muñoz et al. (2011)_Sample1	Sleep	Composite	0,620	0,593	0,646	32,682	0,000
			0,301	0,216	0,383	6,632	0,000

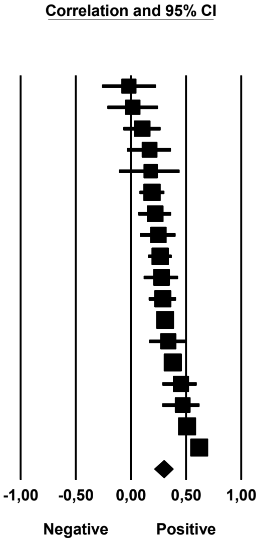

Supplementary Figure 1cd. Forest plot for sleep.

Study name	Variable	Detachment	Correlation	Lower limit	Upper limit	Z-Value	p-Value
Bono et al. (2013)	Physical Discomfort	Composite	-0,670	-0,789	-0,503	-6,174	0,000
Els et al. (2015)Mostert & Els (2015)	Physical Discomfort	Composite	-0,260	-0,353	-0,162	-5,070	0,000
Moreno-Jiménez et al. (2012)	Physical Discomfort	Composite	-0,250	-0,308	-0,191	-8,024	0,000
De Bloom et al. (2015)	Physical Discomfort	Composite	-0,190	-0,244	-0,135	-6,677	0,000
Shimazu et al. (2012)	Physical Discomfort	Composite	-0,140	-0,178	-0,102	-7,070	0,000
de Jonge et al. (2012)	Physical Discomfort	Composite	-0,103	-0,199	-0,005	-2,055	0,040
			-0,231	-0,308	-0,150	-5,508	0,000

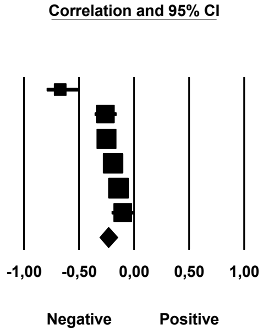

Supplementary Figure 1e. Forest plot for physical discomfort.

Study name	Variable	Detachment	Correlation	Lower limit	Upper limit	Z-Value	p-Value
Rivkin et al. (2014)_Study 1	Burnout_other	Composite	-0,370	-0,448	-0,287	-8,166	0,000
Rivkin et al. (2014)_Study 2	Burnout_other	Composite	-0,310	-0,393	-0,222	-6,593	0,000
Els et al. (2015)Mostert & Els (2015)	Burnout_other	Composite	-0,170	-0,268	-0,069	-3,271	0,001
Sonnentag & Fritz (2007)	Burnout_other	Composite	-0,160	-0,319	0,008	-1,868	0,062
Derks & Bakker (2014)	Burnout_other	Composite	-0,040	-0,274	0,199	-0,325	0,745
Barber & Santuzzi (2015)	Burnout_other	Composite	0,000	-0,113	0,113	0,000	1,000
Virga & Paveloni (2015)	Burnout_other	Composite	0,170	-0,009	0,338	1,865	0,062
			-0,140	-0,275	-0,000	-1,962	0,050

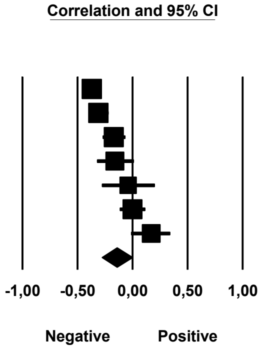

Supplementary Figure 1f. Forest plot for burnout(others).

Study name	Variable	Detachment	Correlation	Lower limit	Upper limit	Z-Value	p-Value
Vahle-Hinz et al. (2014)	Physiological Activation	Composite	-0,050	-0,324	0,232	-0,343	0,732
Bono et al. (2013)	Physiological Activation	Composite	0,190	-0,065	0,422	1,465	0,143
Cropley et al. (2013)	Physiological Activation	Composite	-0,038	-0,225	0,152	-0,390	0,697
			0,025	-0,121	0,171	0,337	0,736

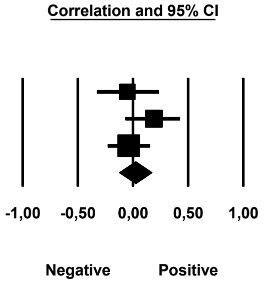

Supplementary Figure 1g. Forest plot for physiological activation.

Study name	Variable	Detachment	Correlation	Lower limit	Upper limit	Z-Value	p-Value
Rodríguez-Muñoz et al. (2011)_Sample 1	Fatigue	Composite	-0,790	-0,806	-0,773	-48,298	0,000
Rodríguez-Muñoz et al. (2011)_Sample 2	Fatigue	Composite	-0,620	-0,646	-0,592	-32,665	0,000
Rivkin et al. (2014)_Study 2	Fatigue	Composite	-0,585	-0,644	-0,519	-13,789	0,000
Sonnentag et al. (2010b)	Fatigue	Composite	-0,530	-0,641	-0,397	-6,806	0,000
Sonnentag & Fritz (2007)	Fatigue	Composite	-0,520	-0,633	-0,386	-6,672	0,000
Sonnentag & Bayer (2005)	Fatigue	Composite	-0,504	-0,646	-0,328	-5,082	0,000
Rivkin et al. (2014)_Study 1	Fatigue	Composite	-0,495	-0,562	-0,422	-11,421	0,000
Kompier et al. (2012)	Fatigue	Composite	-0,470	-0,491	-0,449	-36,799	0,000
Querstedt & Cropley (2012)	Fatigue	Composite	-0,464	-0,519	-0,404	-13,431	0,000
Hülsheger et al. (2014)	Fatigue	Composite	-0,360	-0,508	-0,191	-4,024	0,000
Kinunnen & Feldt (2013)	Fatigue	Composite	-0,340	-0,443	-0,228	-5,699	0,000
Nohe et al. (2014)	Fatigue	Composite	-0,180	-0,368	0,022	-1,746	0,081
Vahle-Hinz et al. (2014)	Fatigue	Composite	-0,170	-0,428	0,113	-1,180	0,238
Lapierre et al. (2012)	Fatigue	Composite	-0,170	-0,340	0,011	-1,841	0,066
Korunka et al. (2012)	Fatigue	Composite	-0,133	-0,367	0,117	-1,046	0,296
Coffeng et al. (2015)	Fatigue	Composite	-0,127	-0,221	-0,031	-2,582	0,010
Sonnentag et al. (2008a)	Fatigue	Composite	-0,090	-0,239	0,063	-1,152	0,249
			-0,415	-0,522	-0,296	-6,322	0,000

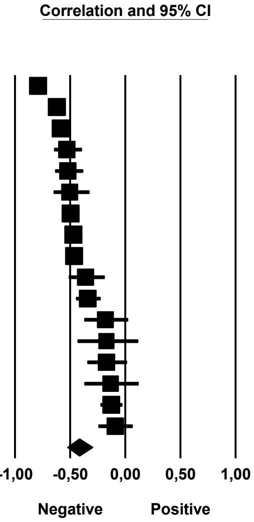

Supplementary Figure 1h. Forest plot for fatigue.

Study name	Variable	Detachment	Correlation	Lower limit	Upper limit	Z-Value	p-Value
Sonnentag & Grant (2012)	Affect	Composite	-0,307	-0,510	-0,072	-2,540	0,011
Mojza et al. (2011)	Affect	Composite	0,051	-0,142	0,241	0,520	0,603
Meier et al. (2015) Study 1	Affect	Composite	0,053	-0,120	0,222	0,598	0,550
Demerouti et al. (2012)	Affect	Composite	0,082	-0,136	0,292	0,732	0,464
Meier et al. (2015) Study 2	Affect	Composite	0,096	-0,119	0,301	0,873	0,382
Park et al. (2015)	Affect	Composite	0,100	-0,103	0,295	0,968	0,333
Hernández et al. (2013)	Affect	Composite	0,188	-0,004	0,366	1,918	0,055
Sonnentag & Binnewies (2013)	Affect	Composite	0,198	-0,002	0,384	1,940	0,052
Meier et al. (2015) Study 3	Affect	Composite	0,215	-0,014	0,423	1,840	0,066
Ohly & Latour (2014)	Affect	Composite	0,232	0,187	0,277	9,791	0,000
Danner-Vlaardingerbroek et al. (2013)	Affect	Composite	0,250	0,175	0,322	6,375	0,000
Sonnentag et al. (2008a)	Affect	Composite	0,277	0,130	0,412	3,628	0,000
van Hooff (2013)	Affect	Composite	0,290	0,069	0,484	2,550	0,011
Smit (2015)	Affect	Composite	0,290	0,102	0,458	2,986	0,003
Hahn et al. (2011)	Affect	Composite	0,340	0,149	0,507	3,396	0,001
Volmer et al. (2012)	Affect	Composite	0,340	0,152	0,504	3,451	0,001
Sonnentag et al. (2008b)	Affect	Composite	0,349	0,205	0,479	4,552	0,000
Pereira et al. (2014)	Affect	Composite	0,360	0,146	0,542	3,220	0,001
Hahn et al. (2014)	Affect	Composite	0,429	0,260	0,573	4,656	0,000
Querstedt & Cropley (2012)	Affect	Composite	0,460	0,400	0,516	13,302	0,000
ten Brummelhuis & Bakker (2012)	Affect	Composite	0,470	0,271	0,631	4,298	0,000
Wang et al. (2013)	Affect	Composite	0,482	0,349	0,597	6,356	0,000
Sonnentag & Bayer (2005)	Affect	Composite	0,539	0,371	0,673	5,527	0,000
de Bloom et al. (2014)	Affect	Composite	0,540	0,318	0,706	4,315	0,000
			0,275	0,209	0,338	7,866	0,000

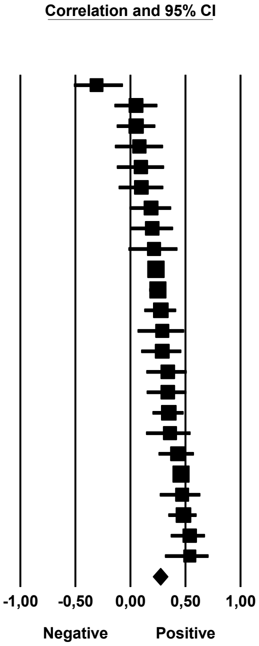

Supplementary Figure 1i. Forest plot for affect.

Study name	Variable	Detachment	Correlation	Lower limit	Upper limit	Z-Value	p-Value
Binnewies et al. (2009)	State recovery	Composite	-0,006	-0,110	0,097	-0,118	0,906
Jalonen et al. (2015)	State recovery	Composite	0,140	-0,005	0,279	1,891	0,059
Ohly & Latour (2014)	State recovery	Composite	0,260	0,215	0,304	11,007	0,000
Kinunnen & Feldt (2013)	State recovery	Composite	0,310	0,196	0,416	5,159	0,000
Nicholson & Griffin (2014)	State recovery	Composite	0,440	0,312	0,552	6,193	0,000
Niks et al. (2015)	State recovery	Composite	0,480	0,271	0,646	4,184	0,000
Volman et al. (2013)	State recovery	Composite	0,610	0,430	0,743	5,582	0,000
			0,311	0,181	0,430	4,554	0,000

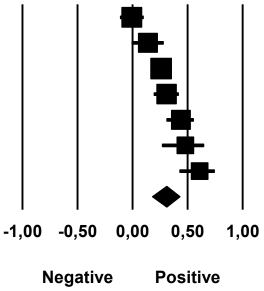

Supplementary Figure 1j. Forest plot for state of recovery.

Study name	Variable	Detachment	Correlation	Lower limit	Upper limit	Z-Value	p-Value
Barber & Santuzzi (2015)	Motivation	Composite	-0,240	-0,343	-0,131	-4,240	0,000
Virga & Paveloni (2015)	Motivation	Composite	-0,101	-0,274	0,079	-1,098	0,272
Demerouti et al. (2012)	Motivation	Composite	-0,080	-0,291	0,138	-0,717	0,473
Shimazu et al. (2012)	Motivation	Composite	-0,080	-0,119	-0,041	-4,022	0,000
Sanz-Vergel et al. (2011)	Motivation	Composite	0,010	-0,272	0,290	0,068	0,946
Sonnentag et al. (2010a)	Motivation	Composite	0,060	-0,052	0,170	1,051	0,293
Kinunnen & Feldt (2013)	Motivation	Composite	0,064	-0,058	0,183	1,025	0,305
White (2011)	Motivation	Composite	0,120	-0,170	0,391	0,809	0,419
De Bloom et al. (2015)	Motivation	Composite	0,125	0,070	0,181	4,378	0,000
Kinnunen et al. (2015)	Motivation	Composite	0,170	0,112	0,227	5,701	0,000
ten Brummelhuis & Bakker (2012)	Motivation	Composite	0,430	0,223	0,600	3,875	0,000
			0,038	-0,059	0,134	0,763	0,446

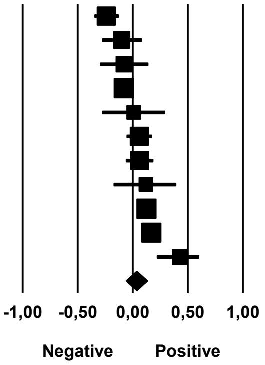

Supplementary Figure 1k. Forest plot for work motivation.

Study name	Variable	Detachment	Correlation	Lower limit	Upper limit	Z-Value	p-Value
Sanz-Vergel et al. (2011)	Task Performance	Composite	-0,100	-0,371	0,186	-0,681	0,496
Binnewies et al. (2009)	Task Performance	Composite	-0,019	-0,122	0,085	-0,355	0,723
Fritz et al. (2010)	Task Performance	Composite	0,040	-0,151	0,228	0,408	0,683
De Bloom et al. (2015)	Task Performance	Composite	0,070	0,014	0,126	2,434	0,015
Shimazu et al. (2012)	Task Performance	Composite	0,120	0,081	0,158	6,050	0,000
Volman et al. (2013)	Task Performance	Composite	0,130	-0,118	0,362	1,029	0,303
Wang et al. (2013)	Task Performance	Composite	0,146	-0,015	0,300	1,773	0,076
Nohe et al. (2014)	Task Performance	Composite	0,260	0,062	0,439	2,552	0,011
			0,085	0,033	0,137	3,179	0,001

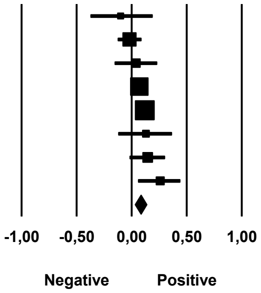

Supplementary Figure 1l. Forest plot for task performance.

Study name	Variable	Detachment	Correlation	Lower limit	Upper limit	Z-Value	p-Value
Binnewies et al. (2009)	Contextual Performance	Composite	-0,220	-0,316	-0,119	-4,208	0,000
Eschleman et al. (2014)_Study 1	Contextual Performance	Composite	-0,183	-0,284	-0,079	-3,409	0,001
Fritz et al. (2010)	Contextual Performance	Composite	-0,090	-0,275	0,102	-0,920	0,357
Eschleman et al. (2014)_Study 2	Contextual Performance	Composite	-0,072	-0,272	0,135	-0,676	0,499
De Bloom et al. (2015)	Contextual Performance	Composite	-0,060	-0,116	-0,004	-2,085	0,037
			-0,130	-0,208	-0,051	-3,199	0,001

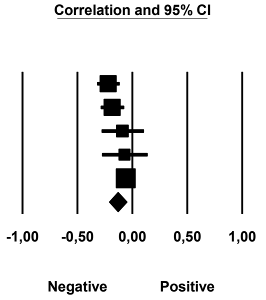

Supplementary Figure 1m. Forest plot for contextual performance.

Study name	Variable	Detachment	Correlation	Lower limit	Upper limit	Z-Value	p-Value
Eschleman et al. (2014)_Study 1	Creativity	Composite	-0,210	-0,309	-0,106	-3,919	0,000
Binnewies et al. (2009)	Creativity	Composite	-0,195	-0,292	-0,093	-3,712	0,000
De Bloom et al. (2015)	Creativity	Composite	-0,080	-0,136	-0,024	-2,783	0,005
de Jonge et al. (2012)	Creativity	Composite	-0,034	-0,132	0,064	-0,683	0,495
Eschleman et al. (2014)_Study 2	Creativity	Composite	0,000	-0,205	0,205	0,000	1,000
			-0,113	-0,186	-0,039	-3,004	0,003

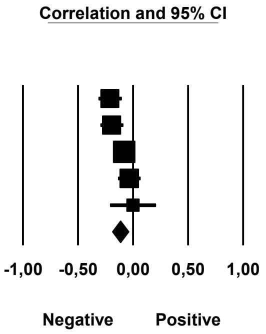

Supplementary Figure 1n. Forest plot for creativity.

Study name	Variable	Detachment	Correlation	Lower limit	Upper limit	Z-Value	p-Value
Wang, Liu, Liao, Gong, Kammeyer-Mueller, & Shi	Job Demands	Composite	-0,558	-0,659	-0,436	-7,608	0,000
Kinnunen, & Feldt	Job Demands	Composite	-0,514	-0,598	-0,419	-9,150	0,000
Rodríguez-Muñoz, Notelaers, & Moreno-Jiménez (sample 1)	Job Demands	Composite	-0,510	-0,541	-0,477	-25,367	0,000
Vahle-Hinz, Bamberg, Dettmers, Friedrich, & Keller	Job Demands	Composite	-0,510	-0,690	-0,270	-3,858	0,000
White	Job Demands	Composite	-0,425	-0,632	-0,160	-3,040	0,002
Nicholson, & Griffin	Job Demands	Composite	-0,422	-0,537	-0,292	-5,901	0,000
Sanz-Vergel, Demerouti, Bakker, & Moreno-Jiménez	Job Demands	Composite	-0,420	-0,627	-0,157	-3,036	0,002
Sonnentag, & Fritz	Job Demands	Composite	-0,392	-0,489	-0,287	-6,786	0,000
Querstedt, & Cropley	Job Demands	Composite	-0,382	-0,443	-0,318	-10,782	0,000
Els et al. (2015)	Job Demands	Composite	-0,379	-0,464	-0,288	-7,601	0,000
Rivkin, Diestel, & Schmidt (Study 1)	Job Demands	Composite	-0,370	-0,448	-0,287	-8,166	0,000
Rodríguez-Muñoz, Notelaers, & Moreno-Jiménez (sample 2)	Job Demands	Composite	-0,370	-0,407	-0,332	-17,501	0,000
Bono, Glomb, Shen, Kim, & Koch	Job Demands	Composite	-0,370	-0,569	-0,130	-2,958	0,003
Nohe, Michel, & Sonntag	Job Demands	Composite	-0,366	-0,528	-0,177	-3,678	0,000
Rivkin, Diestel, & Schmidt (Study 2)	Job Demands	Composite	-0,355	-0,435	-0,269	-7,624	0,000
Kinnunen, Mauno, & Siltaloppi	Job Demands	Composite	-0,352	-0,426	-0,273	-8,246	0,000
Jalonen, Kinnunen, Pulkkinen, & Kokko	Job Demands	Composite	-0,345	-0,473	-0,203	-4,583	0,000
Jalonen et al. (2015)	Job Demands	Composite	-0,345	-0,467	-0,211	-4,831	0,000
Demsky, Ellis, & Fritz	Job Demands	Composite	-0,340	-0,498	-0,161	-3,611	0,000
Sonnentag, Kuttler, & Fritz	Job Demands	Composite	-0,333	-0,474	-0,174	-3,987	0,000
Oosthuizen, Mostert, & Koekemor	Job Demands	Composite	-0,329	-0,417	-0,234	-6,502	0,000
Sonnentag, & Bayer	Job Demands	Composite	-0,309	-0,488	-0,105	-2,926	0,003
Kompier, Taris, & van Veldhoven	Job Demands	Composite	-0,308	-0,332	-0,283	-22,974	0,000
Pereira, Semmer, & Elfering	Job Demands	Composite	-0,302	-0,494	-0,082	-2,663	0,008
Barber & Santuzzi (2015)	Job Demands	Composite	-0,300	-0,399	-0,194	-5,361	0,000
Smit (2015)	Job Demands	Composite	-0,290	-0,458	-0,102	-2,986	0,003
Sonnentag, Unger, & Nägel	Job Demands	Composite	-0,272	-0,375	-0,162	-4,732	0,000
Hülsheger, Lang, Depenbrock, Fehrmann, Zijlstra, & Alberts	Job Demands	Composite	-0,270	-0,430	-0,093	-2,956	0,003
Sonnentag, Binnewies, & Mojza	Job Demands	Composite	-0,252	-0,354	-0,145	-4,512	0,000
Potok, & Littman-Ovadia	Job Demands	Composite	-0,252	-0,420	-0,067	-2,655	0,008
Cropley, Michalianou, Pravettoni, & Millward	Job Demands	Composite	-0,251	-0,360	-0,135	-4,176	0,000
Cropley, & Millward Purvis	Job Demands	Composite	-0,240	-0,451	-0,004	-1,989	0,047
Hülsheger et al. (2015)	Job Demands	Composite	-0,240	-0,390	-0,077	-2,865	0,004
Sonnentag, & Kruel	Job Demands	Composite	-0,230	-0,377	-0,071	-2,820	0,005
Sonnentag, Arbeus, Mahn, & Fritz	Job Demands	Composite	-0,230	-0,401	-0,044	-2,409	0,016
Derks, van Mierlo, & Schmitz	Job Demands	Composite	-0,220	-0,433	0,016	-1,831	0,067
Molino et al. (2015)	Job Demands	Composite	-0,220	-0,295	-0,142	-5,451	0,000
Ohly, & Latour	Job Demands	Composite	-0,210	-0,255	-0,164	-8,818	0,000
Sonnentag, Mojza, Binnewies, & Scholl	Job Demands	Composite	-0,210	-0,354	-0,056	-2,663	0,008
Fritz, Yankelevich, Zarubin, & Barger	Job Demands	Composite	-0,200	-0,376	-0,011	-2,067	0,039
Frone	Job Demands	Composite	-0,188	-0,224	-0,152	-10,132	0,000
Moreno-Jiménez, Rodríguez-Muñoz,Sanz-Vergel, Geurts, & Garrosa	Job Demands	Composite	-0,184	-0,243	-0,123	-5,846	0,000
Cropley, Dijk, & Stanley	Job Demands	Composite	-0,180	-0,334	-0,016	-2,153	0,031
Querstret et al. (2015)	Job Demands	Composite	-0,166	-0,290	-0,036	-2,505	0,012
van Hooff	Job Demands	Composite	-0,162	-0,374	0,066	-1,396	0,163
Shimazu, de Jonge, Kubota, & Kawakami (Sample 2)	Job Demands	Composite	-0,132	-0,186	-0,077	-4,697	0,000
Park, & Fritz	Job Demands	Composite	-0,120	-0,196	-0,043	-3,034	0,002
Moreno-Jiménez, Rodríguez-Muñoz, Pastor, Sanz-Vergel, Geurts, & Garrosa	Job Demands	Composite	-0,116	-0,201	-0,030	-2,632	0,008
Mojza, Sonnentag, & Bornemann	Job Demands	Composite	-0,115	-0,300	0,078	-1,168	0,243
Allen, Holland, & Reynolds	Job Demands	Composite	-0,114	-0,188	-0,039	-2,964	0,003
ten Brummelhuis, & Bakker	Job Demands	Composite	-0,110	-0,330	0,122	-0,931	0,352
Shimazu, de Jonge, Kubota, & Kawakami (Sample 1)	Job Demands	Composite	-0,081	-0,136	-0,026	-2,891	0,004
Demerouti, Bakker, Sonnentag, & Fullgar	Job Demands	Composite	-0,080	-0,291	0,138	-0,717	0,473
Park et al. (2015)	Job Demands	Composite	-0,068	-0,265	0,134	-0,656	0,512
Moreno-Jiménez, Mayo, Sanz-Vergel, Geurts, Rodríguez-Muñoz, & Garrosa	Job Demands	Composite	-0,020	-0,193	0,154	-0,224	0,823
de Jonge, Spoor, Sonnentag, Dormann, & van der Tooren	Job Demands	Composite	-0,014	-0,112	0,084	-0,282	0,778
Sonnentag, & Grant	Job Demands	Composite	-0,010	-0,250	0,231	-0,080	0,936
Mojza, Lorenz, Sonnentag, & Binnewies	Job Demands	Composite	0,030	-0,123	0,181	0,383	0,702
de Bloom, Radstaak, & Geurts	Job Demands	Composite	0,040	-0,230	0,304	0,286	0,775
Volmer, Binnewies, Sonnentag, & Niessen	Job Demands	Composite	0,190	-0,009	0,374	1,875	0,061
			-0,270	-0,281	-0,259	-46,625	0,000

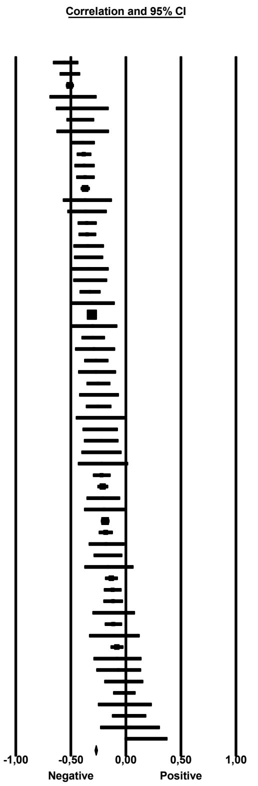

Supplementary Figure 1o. Forest plot for job demands.

Study name	Variable	Detachment	Correlation	Lower limit	Upper limit	Z-Value	p-Value
White	Quantitative demands	Composite	-0,420	-0,629	-0,154	-3,003	0,003
Els et al. (2015)	Quantitative demands	Composite	-0,410	-0,492	-0,321	-8,300	0,000
Jalonen, Kinnunen, Pulkkinen, & Kokko	Quantitative demands	Composite	-0,380	-0,504	-0,241	-5,092	0,000
Jalonen et al. (2015)	Quantitative demands	Composite	-0,380	-0,498	-0,249	-5,367	0,000
Kompier, Taris, & van Veldhoven	Quantitative demands	Composite	-0,360	-0,383	-0,336	-27,191	0,000
Kinnunen, & Feldt	Quantitative demands	Composite	-0,520	-0,603	-0,426	-9,275	0,000
Barber & Santuzzi (2015)	Quantitative demands	Composite	-0,345	-0,440	-0,242	-6,229	0,000
Oosthuizen, Mostert, & Koekemor	Quantitative demands	Composite	-0,330	-0,418	-0,235	-6,532	0,000
Sonnentag, Kuttler, & Fritz	Quantitative demands	Composite	-0,330	-0,472	-0,171	-3,954	0,000
Frone	Quantitative demands	Composite	-0,182	-0,218	-0,146	-9,804	0,000
Sonnentag, & Bayer	Quantitative demands	Composite	-0,300	-0,480	-0,095	-2,836	0,005
Sonnentag, Arbeus, Mahn, & Fritz	Quantitative demands	Composite	-0,290	-0,453	-0,108	-3,074	0,002
Smit (2015)	Quantitative demands	Composite	-0,290	-0,458	-0,102	-2,986	0,003
Vahle-Hinz, Bamberg, Dettmers, Friedrich, & Keller	Quantitative demands	Composite	-0,510	-0,690	-0,270	-3,858	0,000
Sonnentag, Unger, & Nägel	Quantitative demands	Composite	-0,270	-0,373	-0,160	-4,699	0,000
Hülsheger, Lang, Depenbrock, Fehrmann, Zijlstra, & Alberts	Quantitative demands	Composite	-0,270	-0,430	-0,093	-2,956	0,003
Querstret et al. (2015)	Quantitative demands	Composite	-0,250	-0,368	-0,124	-3,823	0,000
Potok, & Littman-Ovadia	Quantitative demands	Composite	-0,240	-0,410	-0,054	-2,520	0,012
Pereira, Semmer, & Elfering	Quantitative demands	Composite	-0,240	-0,442	-0,015	-2,091	0,036
Derks, van Mierlo, & Schmitz	Quantitative demands	Composite	-0,220	-0,433	0,016	-1,831	0,067
Molino et al. (2015)	Quantitative demands	Composite	-0,220	-0,295	-0,142	-5,451	0,000
Sonnentag, Mojza, Binnewies, & Scholl	Quantitative demands	Composite	-0,210	-0,354	-0,056	-2,663	0,008
Sonnentag, Binnewies, & Mojza	Quantitative demands	Composite	-0,210	-0,314	-0,101	-3,729	0,000
Sonnentag, & Fritz	Quantitative demands	Composite	-0,490	-0,576	-0,394	-8,776	0,000
Fritz, Yankelevich, Zarubin, & Barger	Quantitative demands	Composite	-0,200	-0,376	-0,011	-2,067	0,039
Shimazu, de Jonge, Kubota, & Kawakami (Sample 2)	Quantitative demands	Composite	-0,180	-0,233	-0,126	-6,452	0,000
Querstedt, & Cropley	Quantitative demands	Composite	-0,430	-0,488	-0,368	-12,300	0,000
Shimazu, de Jonge, Kubota, & Kawakami (Sample 1)	Quantitative demands	Composite	-0,160	-0,213	-0,106	-5,722	0,000
Sonnentag, & Kruel	Quantitative demands	Composite	-0,130	-0,285	0,032	-1,574	0,115
Moreno-Jiménez, Rodríguez-Muñoz, Pastor, Sanz-Vergel, Geurts, & Garrosa	Quantitative demands	Composite	-0,040	-0,126	0,047	-0,902	0,367
van Hooff	Quantitative demands	Composite	-0,024	-0,248	0,203	-0,205	0,838
Sonnentag, & Grant	Quantitative demands	Composite	-0,010	-0,250	0,231	-0,080	0,936
Park et al. (2015)	Quantitative demands	Composite	0,000	-0,200	0,200	0,000	1,000
			-0,275	-0,320	-0,229	-11,207	0,000

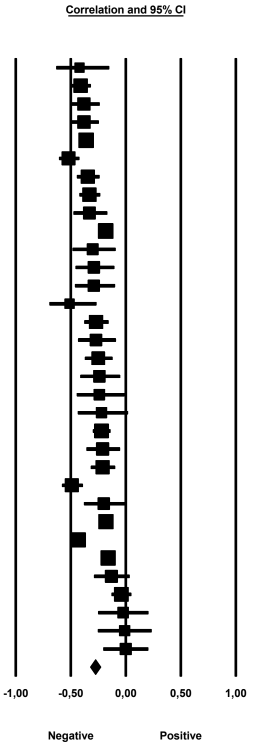

Supplementary Figure 1p. Forest plot for quantitative demands.

Study name	Variable	Detachment	Correlation	Lower limit	Upper limit	Z-Value	p-Value
Wang, Liu, Liao, Gong, Kammeyer-Mueller, & Shi	Social stressors	Composite	-0,558	-0,659	-0,436	-7,608	0,000
Rodríguez-Muñoz, Notelaers, & Moreno-Jiménez (sample 1)	Social stressors	Composite	-0,510	-0,541	-0,477	-25,367	0,000
Rodríguez-Muñoz, Notelaers, & Moreno-Jiménez (sample 2)	Social stressors	Composite	-0,370	-0,407	-0,332	-17,501	0,000
Pereira, Semmer, & Elfering	Social stressors	Composite	-0,360	-0,542	-0,146	-3,220	0,001
Demsky, Ellis, & Fritz	Social stressors	Composite	-0,340	-0,498	-0,161	-3,611	0,000
Nicholson, & Griffin	Social stressors	Composite	-0,200	-0,338	-0,053	-2,659	0,008
Moreno-Jiménez, Rodríguez-Muñoz,Sanz-Vergel, Geurts, & Garrosa	Social stressors	Composite	-0,170	-0,230	-0,109	-5,393	0,000
Moreno-Jiménez, Rodríguez-Muñoz, Pastor, Sanz-Vergel, Geurts, & Garrosa	Social stressors	Composite	-0,160	-0,243	-0,074	-3,637	0,000
Sonnentag, Unger, & Nägel	Social stressors	Composite	-0,143	-0,254	-0,028	-2,442	0,015
Allen, Holland, & Reynolds	Social stressors	Composite	-0,140	-0,213	-0,065	-3,645	0,000
Park et al. (2015)	Social stressors	Composite	-0,110	-0,304	0,093	-1,065	0,287
Volmer, Binnewies, Sonnentag, & Niessen	Social stressors	Composite	0,190	-0,009	0,374	1,875	0,061
			-0,252	-0,359	-0,139	-4,278	0,000

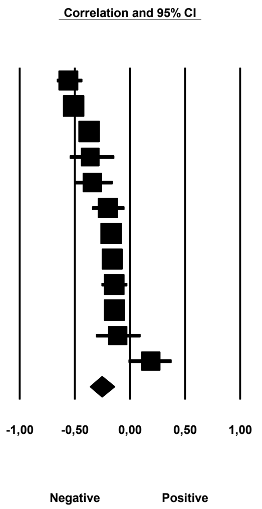

Supplementary Figure 1q. Forest plot for social conflicts.

Study name	Variable	Detachment	Correlation	Lower limit	Upper limit	Z-Value	p-Value
Bono, Glomb, Shen, Kim, & Koch	Emotional demands	Composite	-0,370	-0,569	-0,130	-2,958	0,003
Els et al. (2015)	Emotional demands	Composite	-0,300	-0,391	-0,204	-5,897	0,000
Kompier, Taris, & van Veldhoven	Emotional demands	Composite	-0,280	-0,305	-0,255	-20,755	0,000
Oosthuizen, Mostert, & Koekemor	Emotional demands	Composite	-0,240	-0,334	-0,141	-4,664	0,000
Frone	Emotional demands	Composite	-0,232	-0,267	-0,197	-12,590	0,000
Barber & Santuzzi (2015)	Emotional demands	Composite	-0,120	-0,230	-0,007	-2,089	0,037
de Jonge, Spoor, Sonnentag, Dormann, & van der Tooren	Emotional demands	Composite	-0,017	-0,115	0,081	-0,341	0,733
			-0,216	-0,277	-0,153	-6,576	0,000

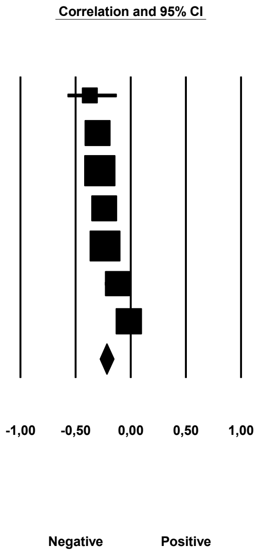

Supplementary Figure 1r. Forest plot for emotional demands.

Study name	Variable	Detachment	Correlation	Lower limit	Upper limit	Z-Value	p-Value
Nicholson, & Griffin	Working time	Composite	-0,420	-0,535	-0,290	-5,871	0,000
Sanz-Vergel, Demerouti, Bakker, & Moreno-Jiménez	Working time	Composite	-0,420	-0,627	-0,157	-3,036	0,002
Kinnunen, & Feldt	Working time	Composite	-0,420	-0,515	-0,315	-7,205	0,000
Kinnunen, Mauno, & Siltaloppi	Working time	Composite	-0,390	-0,461	-0,313	-9,236	0,000
Nohe, Michel, & Sonntag	Working time	Composite	-0,280	-0,456	-0,083	-2,759	0,006
Cropley, Michalianou, Pravettoni, & Millward	Working time	Composite	-0,251	-0,360	-0,135	-4,176	0,000
Hülsheger et al. (2015)	Working time	Composite	-0,240	-0,390	-0,077	-2,865	0,004
Sonnentag, & Fritz	Working time	Composite	-0,230	-0,340	-0,114	-3,834	0,000
van Hooff	Working time	Composite	-0,228	-0,431	-0,003	-1,982	0,047
Sonnentag, & Bayer	Working time	Composite	-0,214	-0,406	-0,003	-1,990	0,047
Mojza, Sonnentag, & Bornemann	Working time	Composite	-0,210	-0,386	-0,019	-2,153	0,031
Ohly, & Latour	Working time	Composite	-0,210	-0,255	-0,164	-8,818	0,000
Sonnentag, Kuttler, & Fritz	Working time	Composite	-0,170	-0,329	-0,002	-1,980	0,048
Querstedt, & Cropley	Working time	Composite	-0,164	-0,234	-0,092	-4,416	0,000
Potok, & Littman-Ovadia	Working time	Composite	-0,150	-0,329	0,039	-1,556	0,120
Jalonen, Kinnunen, Pulkkinen, & Kokko	Working time	Composite	-0,130	-0,277	0,023	-1,664	0,096
Jalonen et al. (2015)	Working time	Composite	-0,130	-0,270	0,015	-1,754	0,079
Sonnentag, Binnewies, & Mojza	Working time	Composite	-0,120	-0,229	-0,009	-2,109	0,035
Park, & Fritz	Working time	Composite	-0,120	-0,196	-0,043	-3,034	0,002
ten Brummelhuis, & Bakker	Working time	Composite	-0,110	-0,330	0,122	-0,931	0,352
Shimazu, de Jonge, Kubota, & Kawakami (Sample 2)	Working time	Composite	-0,080	-0,135	-0,025	-2,842	0,004
Demerouti, Bakker, Sonnentag, & Fullgar	Working time	Composite	-0,080	-0,291	0,138	-0,717	0,473
Querstret et al. (2015)	Working time	Composite	-0,050	-0,179	0,081	-0,749	0,454
Shimazu, de Jonge, Kubota, & Kawakami (Sample 1)	Working time	Composite	-0,050	-0,105	0,005	-1,774	0,076
Rivkin, Diestel, & Schmidt (Study 2)	Working time	Composite	-0,050	-0,144	0,045	-1,029	0,303
Allen, Holland, & Reynolds	Working time	Composite	-0,030	-0,105	0,046	-0,776	0,438
Sonnentag, Arbeus, Mahn, & Fritz	Working time	Composite	-0,020	-0,207	0,169	-0,206	0,837
Moreno-Jiménez, Mayo, Sanz-Vergel, Geurts, Rodríguez-Muñoz, & Garrosa	Working time	Composite	-0,020	-0,193	0,154	-0,224	0,823
Mojza, Lorenz, Sonnentag, & Binnewies	Working time	Composite	0,030	-0,123	0,181	0,383	0,702
de Bloom, Radstaak, & Geurts	Working time	Composite	0,040	-0,230	0,304	0,286	0,775
			-0,165	-0,210	-0,119	-6,931	0,000

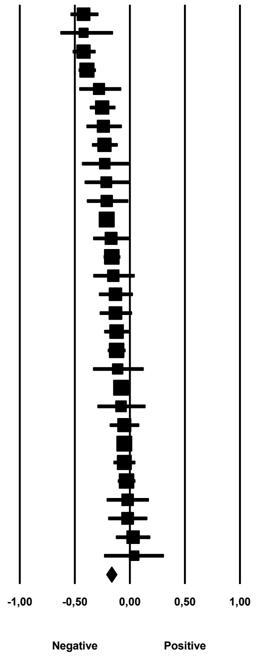

Supplementary Figure 1s. Forest plot for working time.

Study name	Variable	Detachment	Correlation	Lower limit	Upper limit	Z-Value	p-Value
Nohe, Michel, & Sonntag	Role stressors	Composite	-0,260	-0,439	-0,062	-2,552	0,011
Sonnentag, & Kruel	Role stressors	Composite	-0,210	-0,359	-0,050	-2,567	0,010
Potok, & Littman-Ovadia	Role stressors	Composite	-0,197	-0,371	-0,009	-2,055	0,040
Sonnentag, & Fritz	Role stressors	Composite	-0,190	-0,302	-0,072	-3,149	0,002
Rivkin, Diestel, & Schmidt (Study 2)	Role stressors	Composite	-0,180	-0,270	-0,086	-3,743	0,000
Moreno-Jiménez, Rodríguez-Muñoz,Sanz-Vergel, Geurts, & Garrosa	Role stressors	Composite	-0,140	-0,201	-0,078	-4,427	0,000
Moreno-Jiménez, Rodríguez-Muñoz, Pastor, Sanz-Vergel, Geurts, & Garrosa	Role stressors	Composite	-0,080	-0,166	0,007	-1,807	0,071
Frone	Role stressors	Composite	-0,042	-0,079	-0,005	-2,239	0,025
Barber & Santuzzi (2015)	Role stressors	Composite	0,010	-0,103	0,123	0,173	0,862
			-0,124	-0,179	-0,068	-4,334	0,000

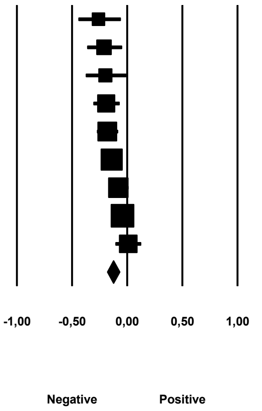

Supplementary Figure 1t. Forest plot for role stressors.

Study name	Variable	Detachment	Correlation	Lower limit	Upper limit	Z-Value	p-Value
Jalonen, Kinnunen, Pulkkinen, & Kokko	Job resources	Composite	-0,100	-0,249	0,054	-1,277	0,202
Jalonen et al. (2015)	Job resources	Composite	-0,100	-0,242	0,046	-1,346	0,178
Park et al. (2015)	Job resources	Composite	-0,100	-0,295	0,103	-0,968	0,333
Richardson, & Thompson	Job resources	Composite	-0,090	-0,253	0,078	-1,052	0,293
de Jonge, Spoor, Sonnentag, Dormann, & van der Tooren	Job resources	Composite	-0,036	-0,133	0,063	-0,712	0,477
Sonnentag, Binnewies, & Mojza	Job resources	Composite	-0,030	-0,141	0,082	-0,525	0,600
Fritz, Yankelevich, Zarubin, & Barger	Job resources	Composite	-0,020	-0,209	0,171	-0,204	0,838
Sonnentag, Unger, & Nägel	Job resources	Composite	0,030	-0,085	0,144	0,509	0,611
Shimazu, de Jonge, Kubota, & Kawakami (Sample 2)	Job resources	Composite	0,038	-0,017	0,093	1,363	0,173
Sonnentag, & Grant	Job resources	Composite	0,070	-0,173	0,305	0,561	0,575
Bono, Glomb, Shen, Kim, & Koch	Job resources	Composite	0,080	-0,175	0,325	0,611	0,541
Shimazu, de Jonge, Kubota, & Kawakami (Sample 1)	Job resources	Composite	0,090	0,035	0,145	3,199	0,001
Sonnentag, & Fritz	Job resources	Composite	0,110	-0,009	0,226	1,808	0,071
Querstedt, & Cropley	Job resources	Composite	0,129	0,057	0,200	3,478	0,001
Kinnunen, & Feldt	Job resources	Composite	0,130	0,009	0,247	2,102	0,036
Querstret et al. (2015)	Job resources	Composite	0,130	-0,000	0,256	1,957	0,050
Kompier, Taris, & van Veldhoven	Job resources	Composite	0,139	0,113	0,166	10,130	0,000
Wang, Liu, Liao, Gong, Kammeyer-Mueller, & Shi	Job resources	Composite	0,200	0,041	0,350	2,450	0,014
White	Job resources	Composite	0,200	-0,089	0,458	1,360	0,174
Sonnentag, Kuttler, & Fritz	Job resources	Composite	0,210	0,043	0,365	2,458	0,014
Oosthuizen, Mostert, & Koekemor	Job resources	Composite	0,219	0,119	0,314	4,233	0,000
Ward, & Steptoe-Warren	Job resources	Composite	0,320	0,121	0,494	3,093	0,002
Els et al. (2015)	Job resources	Composite	0,363	0,271	0,449	7,254	0,000
Frone	Job resources	Composite	0,374	0,342	0,405	20,903	0,000
			0,104	0,039	0,167	3,153	0,002

Statistics for each study

Correlation and 95% CI

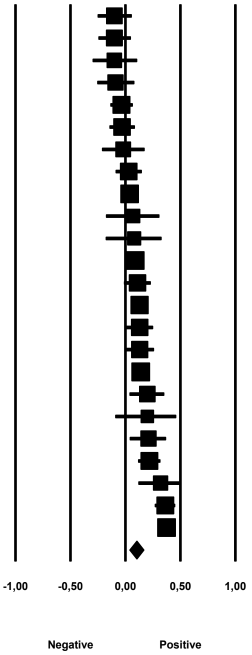

Supplementary Figure 1u. Forest plot for job resources.

Study name	Variable	Detachment	Correlation	Lower limit	Upper limit	Z-Value	p-Value
Shimazu, de Jonge, Kubota, & Kawakami (Sample 2)	Social support	Composite	0,060	0,005	0,115	2,130	0,033
Shimazu, de Jonge, Kubota, & Kawakami (Sample 1)	Social support	Composite	0,130	0,075	0,184	4,635	0,000
Kinnunen, & Feldt	Social support	Composite	0,150	0,029	0,266	2,432	0,015
Wang, Liu, Liao, Gong, Kammeyer-Mueller, & Shi	Social support	Composite	0,201	0,042	0,350	2,462	0,014
Kompier, Taris, & van Veldhoven	Social support	Composite	0,230	0,204	0,256	16,909	0,000
Oosthuizen, Mostert, & Koekemor	Social support	Composite	0,290	0,193	0,381	5,688	0,000
Els et al. (2015)	Social support	Composite	0,390	0,299	0,474	7,846	0,000
			0,206	0,128	0,281	5,096	0,000

Statistics for each study

Correlation and 95% CI

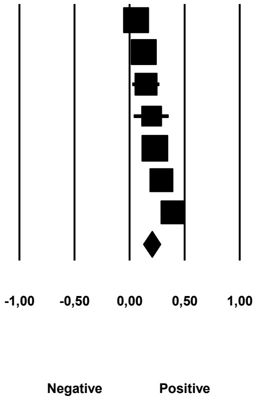

Supplementary Figure 1v. Forest plot for social support.

Study name	Variable	Detachment	Correlation	Lower limit	Upper limit	Z-Value	p-Value
			Statistics for each study				
Jalonen, Kinnunen, Pulkkinen, & Kokko	Job control	Composite	-0,100	-0,249	0,054	-1,277	0,202
Jalonen et al. (2015)	Job control	Composite	-0,100	-0,242	0,046	-1,346	0,178
Park et al. (2015)	Job control	Composite	-0,100	-0,295	0,103	-0,968	0,333
Richardson, & Thompson	Job control	Composite	-0,090	-0,253	0,078	-1,052	0,293
Kinnunen, & Feldt	Job control	Composite	-0,040	-0,160	0,082	-0,644	0,520
Sonnentag, Binnewies, & Mojza	Job control	Composite	-0,030	-0,141	0,082	-0,525	0,600
Fritz, Yankelevich, Zarubin, & Barger	Job control	Composite	-0,020	-0,209	0,171	-0,204	0,838
Shimazu, de Jonge, Kubota, & Kawakami (Sample 2)	Job control	Composite	0,000	-0,055	0,055	0,000	1,000
Shimazu, de Jonge, Kubota, & Kawakami (Sample 1)	Job control	Composite	0,010	-0,045	0,065	0,355	0,723
Sonnentag, Unger, & Nägel	Job control	Composite	0,030	-0,085	0,144	0,509	0,611
Kompier, Taris, & van Veldhoven	Job control	Composite	0,050	0,023	0,077	3,610	0,000
Sonnentag, & Grant	Job control	Composite	0,070	-0,173	0,305	0,561	0,575
Sonnentag, & Fritz	Job control	Composite	0,110	-0,009	0,226	1,808	0,071
Querstedt, & Cropley	Job control	Composite	0,129	0,057	0,201	3,480	0,001
Querstret et al. (2015)	Job control	Composite	0,130	-0,000	0,256	1,957	0,050
Oosthuizen, Mostert, & Koekemor	Job control	Composite	0,190	0,089	0,287	3,665	0,000
White	Job control	Composite	0,200	-0,089	0,458	1,360	0,174
Sonnentag, Kuttler, & Fritz	Job control	Composite	0,210	0,043	0,365	2,458	0,014
Els et al. (2015)	Job control	Composite	0,260	0,162	0,353	5,070	0,000
Ward, & Steptoe-Warren	Job control	Composite	0,320	0,121	0,494	3,093	0,002
			0,059	0,016	0,102	2,701	0,007

Correlation and 95% CI

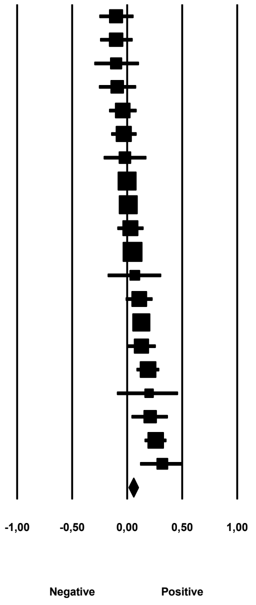

Supplementary Figure 1w. Forest plot for job control.

Study name	Correlation	Lower limit	Upper limit	Z-Value	p-Value
	Statistics for each study				
Park et al. (2011)	-0,480	-0,567	-0,382	-8,530	0,000
Cropley et al. (2013)	-0,440	-0,581	-0,274	-4,839	0,000
Bearber & Jenkins (2014)	-0,435	-0,520	-0,341	-8,232	0,000
Kinnunen et al. (2015)	-0,420	-0,467	-0,370	-14,868	0,000
ten Brummelhuis & Bakker (2012)	-0,400	-0,576	-0,189	-3,570	0,000
Derks & Bakker (2014)	-0,350	-0,542	-0,124	-2,969	0,003
Derks et al. (2014b)	-0,330	-0,524	-0,103	-2,806	0,005
Sonnentag & Bayer (2005)	-0,330	-0,506	-0,128	-3,142	0,002
Richardson & Thompson (2012)	-0,320	-0,462	-0,162	-3,868	0,000
Ward et al. (2013)	-0,300	-0,477	-0,099	-2,887	0,004
Mojza et al. (2010)	-0,270	-0,406	-0,123	-3,535	0,000
Volman et al. (2013)	-0,230	-0,449	0,015	-1,844	0,065
Sonnentag et al. (2010)	-0,210	-0,365	-0,043	-2,458	0,014
Ohly & Latour (2014)	-0,170	-0,216	-0,124	-7,101	0,000
Derks et al. (2014a)	-0,170	-0,376	0,052	-1,506	0,132
Cropley et al. (2006)	-0,130	-0,288	0,035	-1,547	0,122
Mojza et al. (2011)	-0,080	-0,268	0,113	-0,810	0,418
	-0,305	-0,376	-0,232	-7,766	0,000

Correlation and 95% CI

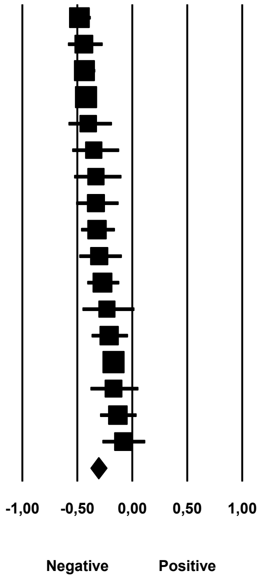

Supplementary Figure 1x. Forest plot for work-related activities.

Study name	Variable	Detachment	Correlation	Lower limit	Upper limit	Z-Value	p-Value
Sonnentag et al. (2008a)	Age	Composite	-0,280	-0,415	-0,133	-3,673	0,000
Mojza et al. (2010)	Age	Composite	-0,270	-0,406	-0,123	-3,535	0,000
Volman et al. (2013)	Age	Composite	-0,230	-0,449	0,015	-1,844	0,065
Vahle-Hinz et al. (2014)	Age	Composite	-0,160	-0,420	0,124	-1,106	0,269
Kinnunen et al. (2010)	Age	Composite	-0,150	-0,234	-0,064	-3,390	0,001
Sonnentag et al. (2013)	Age	Composite	-0,140	-0,251	-0,025	-2,392	0,017
Nohe et al. (2014)	Age	Composite	-0,140	-0,332	0,063	-1,352	0,176
Richardson & Thompson (2012)	Age	Composite	-0,120	-0,281	0,047	-1,406	0,160
Ward et al. (2013)	Age	Composite	-0,110	-0,310	0,099	-1,030	0,303
Sonnentag & Bayer (2005)	Age	Composite	-0,100	-0,304	0,113	-0,920	0,358
Derks et al. (2014a)	Age	Composite	-0,100	-0,313	0,122	-0,880	0,379
Fritz et al. (2010)	Age	Composite	-0,100	-0,284	0,092	-1,023	0,306
Demski et al. (2014)	Age	Composite	-0,100	-0,284	0,092	-1,023	0,306
van Hooff (2013)	Age	Composite	-0,084	-0,304	0,144	-0,719	0,472
Rivkin et al. (2014)_Study 1	Age	Composite	-0,080	-0,172	0,013	-1,686	0,092
Eschleman et al. (2014)_Study 2	Age	Composite	-0,080	-0,280	0,127	-0,756	0,449
Lapierre et al. (2012)	Age	Composite	-0,070	-0,248	0,112	-0,752	0,452
Ohly & Latour (2014)	Age	Composite	-0,050	-0,097	-0,003	-2,070	0,038
Rivkin et al. (2014)_Study 2	Age	Composite	-0,050	-0,144	0,045	-1,029	0,303
Park et al. (2011)	Age	Composite	-0,040	-0,159	0,080	-0,653	0,514
Binnewies et al. (2009)	Age	Composite	-0,038	-0,141	0,066	-0,710	0,478
Cropley et al. (2006)	Age	Composite	-0,020	-0,184	0,145	-0,237	0,813
Shimazu et al. (2014)_Sample 1	Age	Composite	-0,010	-0,065	0,045	-0,355	0,723
Shimazu et al. (2014)_Sample 2	Age	Composite	-0,010	-0,065	0,045	-0,355	0,723
Sanz-Vergel et al. (2011)	Age	Composite	-0,010	-0,290	0,272	-0,068	0,946
Eschleman et al. (2014)_Study 1	Age	Composite	-0,010	-0,116	0,096	-0,184	0,854
Sonnentag & Grant (2012)	Age	Composite	0,000	-0,240	0,240	0,000	1,000
Molino et al. (2015)	Age	Composite	0,010	-0,070	0,090	0,244	0,807
Derks et al. (2014b)	Age	Composite	0,020	-0,216	0,254	0,164	0,870
Mojza et al. (2011)	Age	Composite	0,050	-0,143	0,239	0,505	0,613
ten Brummelhuis & Bakker (2012)	Age	Composite	0,050	-0,181	0,275	0,422	0,673
Sonnentag et al. (2008b)	Age	Composite	0,060	-0,097	0,214	0,750	0,453
Querstedt & Cropley (2012)	Age	Composite	0,072	-0,001	0,145	1,935	0,053
Hülsheger et al. (2015)	Age	Composite	0,080	-0,087	0,243	0,938	0,348
Park et al. (2015)	Age	Composite	0,090	-0,113	0,285	0,870	0,384
Wang et al. (2013)	Age	Composite	0,095	-0,066	0,252	1,157	0,247
Moreno-Jiménez et al. (2009a)	Age	Composite	0,110	-0,065	0,278	1,235	0,217
Demerouti et al. (2012)	Age	Composite	0,110	-0,108	0,318	0,988	0,323
de Jonge et al. (2012)	Age	Composite	0,111	0,013	0,207	2,228	0,026
Frone (2014)	Age	Composite	0,123	0,087	0,159	6,577	0,000
Pereira et al. (2014)	Age	Composite	0,130	-0,098	0,345	1,117	0,264
Sonnentag & Kruel (2006)	Age	Composite	0,150	-0,012	0,304	1,820	0,069
Niks et al. (2015)	Age	Composite	0,160	-0,083	0,385	1,291	0,197
			-0,023	-0,058	0,011	-1,342	0,179

Statistics for each study

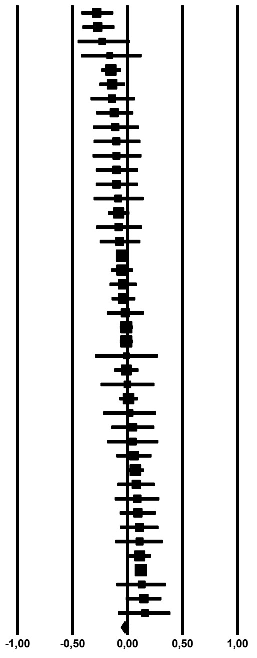

Supplementary Figure 1y. Forest plot for age.

Study name	Variable	Detachment	Correlation	Lower limit	Upper limit	Z-Value	p-Value
Kinnunen et al. (2010)	Gender (Females)	Composite	-0,260	-0,339	-0,177	-5,968	0,000
Moreno-Jiménez et al. (2009a)	Gender (Females)	Composite	-0,250	-0,406	-0,080	-2,856	0,004
Volman et al. (2013)	Gender (Females)	Composite	-0,170	-0,397	0,077	-1,352	0,176
Sonnentag & Bayer (2005)	Gender (Females)	Composite	-0,160	-0,359	0,052	-1,479	0,139
Querstedt & Cropley (2012)	Gender (Females)	Composite	-0,156	-0,226	-0,084	-4,204	0,000
Eschleman et al. (2014)_Study 1	Gender (Females)	Composite	-0,130	-0,233	-0,024	-2,404	0,016
Sonnentag & Kruel (2006)	Gender (Females)	Composite	-0,110	-0,267	0,052	-1,330	0,184
Mojza et al. (2011)	Gender (Females)	Composite	-0,090	-0,277	0,103	-0,911	0,362
Eschleman et al. (2014)_Study 2	Gender (Females)	Composite	-0,080	-0,280	0,127	-0,756	0,449
Vahle-Hinz et al. (2014)	Gender (Females)	Composite	-0,070	-0,342	0,212	-0,481	0,631
Park et al. (2015)	Gender (Females)	Composite	-0,050	-0,248	0,152	-0,483	0,629
Sonnentag & Grant (2012)	Gender (Females)	Composite	-0,040	-0,278	0,202	-0,320	0,749
Hernández et al. (2013)	Gender (Females)	Composite	-0,040	-0,230	0,153	-0,404	0,686
Sonnentag et al. (2010)	Gender (Females)	Composite	-0,030	-0,197	0,139	-0,346	0,729
ten Brummelhuis & Bakker (2012)	Gender (Females)	Composite	-0,010	-0,238	0,219	-0,084	0,933
Rivkin et al. (2014)_Study 2	Gender (Females)	Composite	0,000	-0,095	0,095	0,000	1,000
Park et al. (2011)	Gender (Females)	Composite	0,000	-0,120	0,120	0,000	1,000
Molino et al. (2015)	Gender (Females)	Composite	0,010	-0,070	0,090	0,244	0,807
van Hooff (2013)	Gender (Females)	Composite	0,018	-0,208	0,242	0,154	0,878
Cropley et al. (2006)	Gender (Females)	Composite	0,020	-0,145	0,184	0,237	0,813
Rivkin et al. (2014)_Study 1	Gender (Females)	Composite	0,030	-0,063	0,123	0,631	0,528
de Jonge et al. (2012)	Gender (Females)	Composite	0,030	-0,068	0,128	0,597	0,550
Sanz-Vergel et al. (2011)	Gender (Females)	Composite	0,050	-0,234	0,327	0,339	0,734
Ohly & Latour (2014)	Gender (Females)	Composite	0,050	0,003	0,097	2,070	0,038
Richardson & Thompson (2012)	Gender (Females)	Composite	0,050	-0,117	0,215	0,584	0,560
Pereira et al. (2014)	Gender (Females)	Composite	0,080	-0,148	0,300	0,685	0,493
Derks & Bakker (2014)	Gender (Females)	Composite	0,090	-0,150	0,320	0,733	0,463
Fritz et al. (2010)	Gender (Females)	Composite	0,100	-0,092	0,284	1,023	0,306
Demski et al. (2014)	Gender (Females)	Composite	0,100	-0,092	0,284	1,023	0,306
Frone (2014)	Gender (Females)	Composite	0,116	0,080	0,152	6,208	0,000
Shimazu et al. (2014)_Sample 1	Gender (Females)	Composite	0,120	0,065	0,174	4,275	0,000
Derks et al. (2014a)	Gender (Females)	Composite	0,120	-0,102	0,331	1,058	0,290
Derks et al. (2014b)	Gender (Females)	Composite	0,120	-0,118	0,345	0,987	0,324
Niks et al. (2015)	Gender (Females)	Composite	0,120	-0,124	0,350	0,965	0,335
Shimazu et al. (2014)_Sample 2	Gender (Females)	Composite	0,130	0,075	0,184	4,635	0,000
Burke & El-Kot (2011)	Gender (Females)	Composite	0,130	0,004	0,252	2,021	0,043
Jalonen et al. (2014)	Gender (Females)	Composite	0,130	-0,023	0,277	1,664	0,096
Jalonen et al. (2015)	Gender (Females)	Composite	0,130	-0,015	0,270	1,754	0,079
Sonnentag et al. (2008b)	Gender (Females)	Composite	0,160	0,004	0,308	2,016	0,044
Nohe et al. (2014)	Gender (Females)	Composite	0,170	-0,033	0,359	1,647	0,100
Hahn & Dormann (2013)	Gender (Females)	Composite	0,200	0,072	0,322	3,041	0,002
Sonnentag et al. (2013)	Gender (Females)	Composite	0,210	0,097	0,317	3,618	0,000
Mojza et al. (2010)	Gender (Females)	Composite	0,230	0,080	0,369	2,990	0,003
Sonnentag et al. (2008a)	Gender (Females)	Composite	0,250	0,102	0,388	3,261	0,001
			0,033	-0,006	0,072	1,660	0,097

Statistics for each study

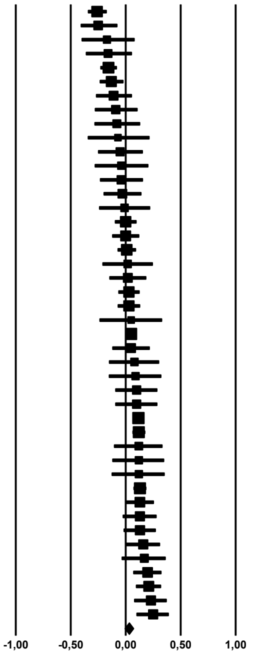

Supplementary Figure 1z. Forest plot for gender.

Study name	Variable	Detachment	Correlation	Lower limit	Upper limit	Z-Value	p-Value
Rodríguez-Muñoz et al. (2011)_Sample1	Negative affectivity/Neuroticism	Composite	-0,650	-0,674	-0,624	-34,949	0,000
Rodríguez-Muñoz et al. (2011)_Sample2	Negative affectivity/Neuroticism	Composite	-0,560	-0,589	-0,529	-28,513	0,000
Sonnentag et al. (2010)	Negative affectivity/Neuroticism	Composite	-0,390	-0,481	-0,291	-7,204	0,000
Sonnentag & Fritz (2007)	Negative affectivity/Neuroticism	Composite	-0,300	-0,447	-0,137	-3,543	0,000
Moreno-Jiménez et al. (2009b)	Negative affectivity/Neuroticism	Composite	-0,250	-0,330	-0,167	-5,757	0,000
Frone (2014)	Negative affectivity/Neuroticism	Composite	-0,216	-0,251	-0,181	-11,695	0,000
Fritz et al. (2010)	Negative affectivity/Neuroticism	Composite	-0,180	-0,358	0,010	-1,856	0,063
Sonnentag & Binnewies (2013)	Negative affectivity/Neuroticism	Composite	-0,180	-0,367	0,021	-1,755	0,079
Binnewies et al. (2009)	Negative affectivity/Neuroticism	Composite	-0,161	-0,260	-0,058	-3,054	0,002
Smit (2015)	Negative affectivity/Neuroticism	Composite	-0,140	-0,325	0,055	-1,409	0,159
Mojza et al. (2010)	Negative affectivity/Neuroticism	Composite	-0,130	-0,277	0,023	-1,669	0,095
Sonnentag et al. (2008a)	Negative affectivity/Neuroticism	Composite	-0,120	-0,267	0,033	-1,539	0,124
Meier et al. (2015) Study 3	Negative affectivity/Neuroticism	Composite	-0,106	-0,327	0,126	-0,897	0,370
Wang et al. (2013)	Negative affectivity/Neuroticism	Composite	-0,075	-0,233	0,086	-0,913	0,361
Meier et al. (2015) Study 2	Negative affectivity/Neuroticism	Composite	-0,054	-0,263	0,160	-0,492	0,622
Sonnentag et al. (2014)	Negative affectivity/Neuroticism	Composite	0,020	-0,169	0,207	0,206	0,837
Hernández et al. (2013)	Negative affectivity/Neuroticism	Composite	0,090	-0,103	0,277	0,911	0,362
			-0,218	-0,348	-0,080	-3,078	0,002

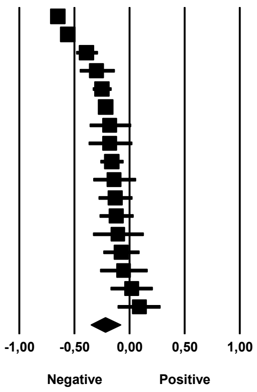

Supplementary Figure 1ab. Forest plot for negative affectivity/neuroticism.

Study name	Variable	Detachment	Correlation	Lower limit	Upper limit	Z-Value	p-Value
Potok, & Littman-Ovadia	Heavy work investment	Composite	-0,520	-0,645	-0,368	-5,934	0,000
de Bloom, Radstaak, & Geurts	Heavy work investment	Composite	-0,370	-0,580	-0,113	-2,774	0,006
Donahue et al. (Study 2)	Heavy work investment	Composite	-0,300	-0,456	-0,126	-3,319	0,001
Shimazu et al. (2014)_Sample 2	Heavy work investment	Composite	-0,300	-0,349	-0,249	-10,974	0,000
Shimazu et al. (2014)_Sample 1	Heavy work investment	Composite	-0,265	-0,316	-0,213	-9,626	0,000
			-0,320	-0,386	-0,251	-8,652	0,000

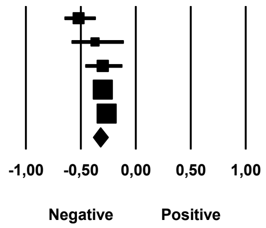

Supplementary Figure 1ac. Forest plot for heavy work investment.

2 Supplementary Figures: Funnel Plots

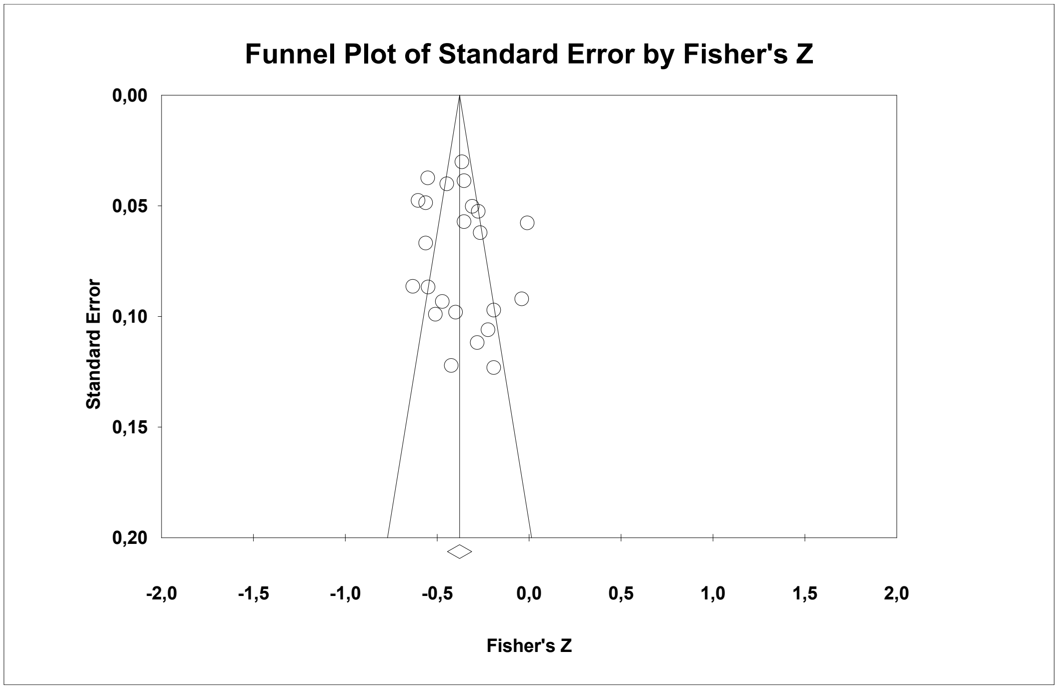

Supplementary Figure 2a. Funnel plot for burnout (exhaustion).

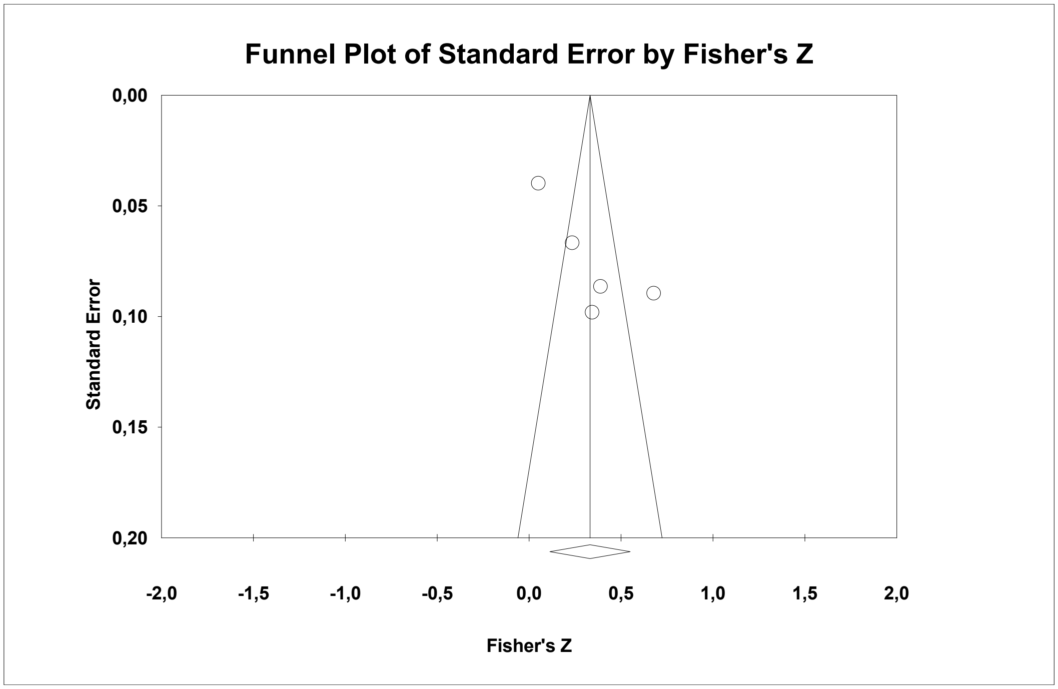

Supplementary Figure 2b. Funnel plot for life satisfaction.

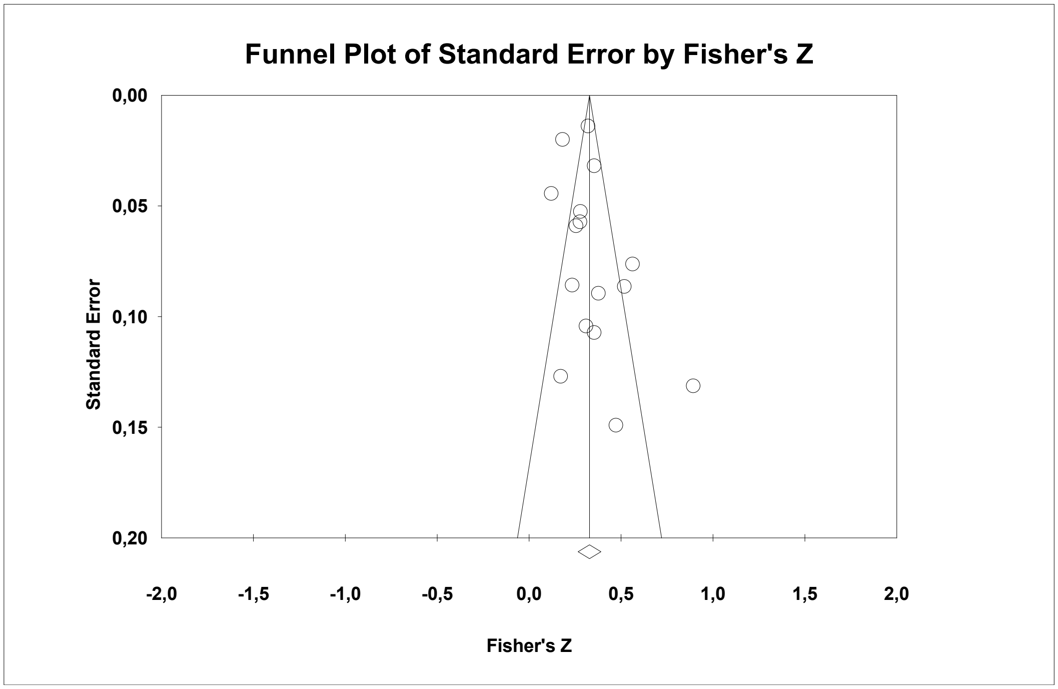

Supplementary Figure 2c. Funnel plot for well-being.

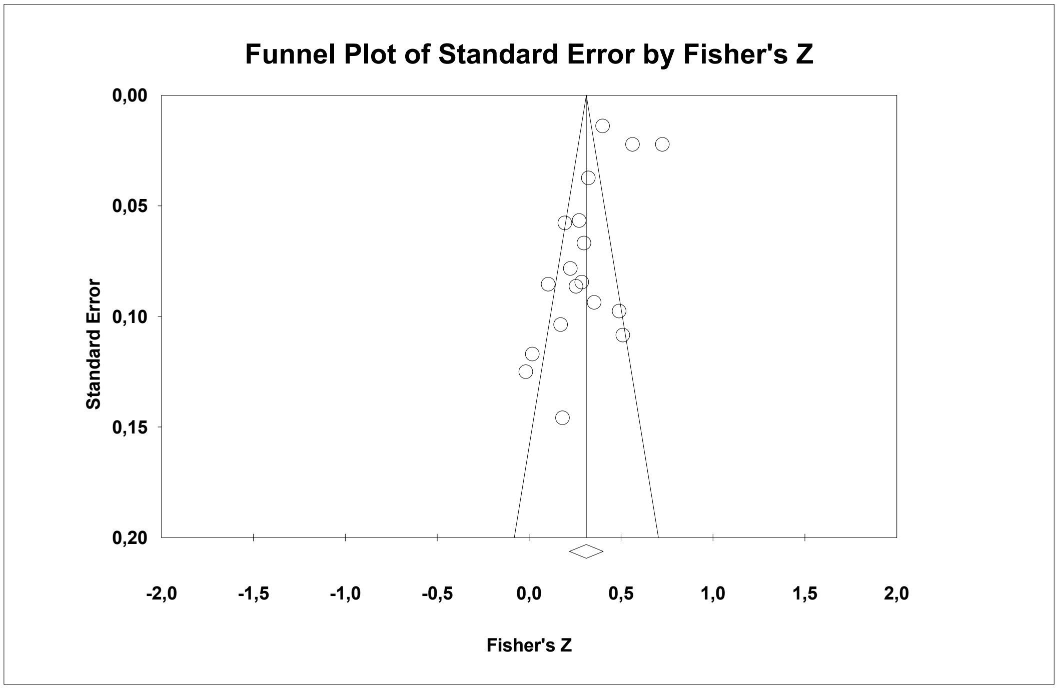

Supplementary Figure 2d. Funnel plot for sleep.

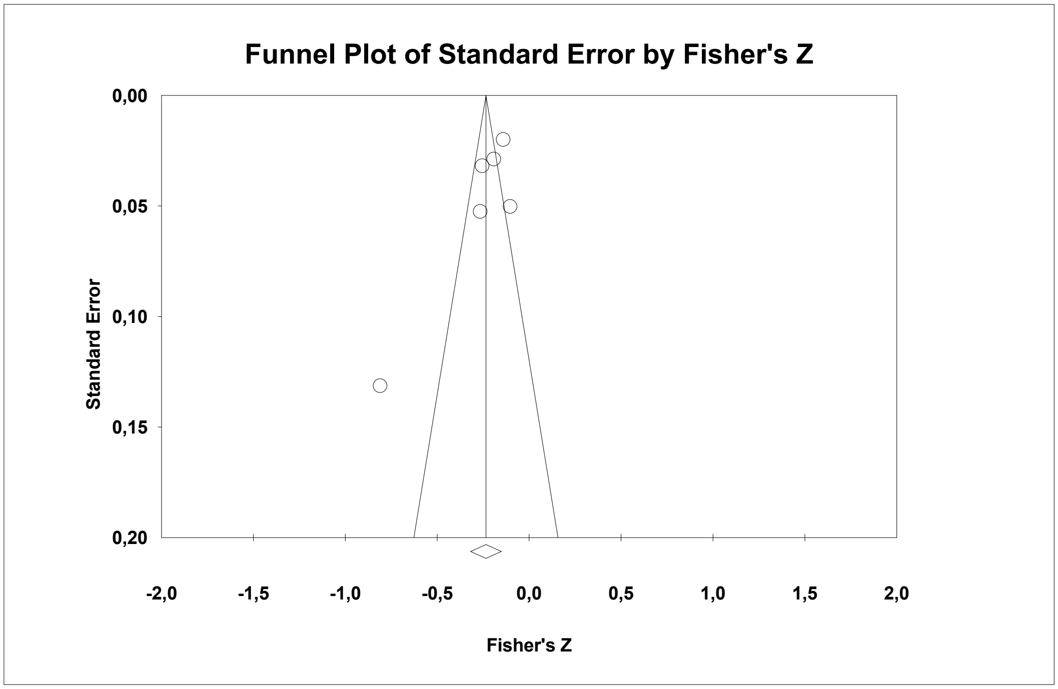

Supplementary Figure 2e. Funnel plot for physical discomfort**.**

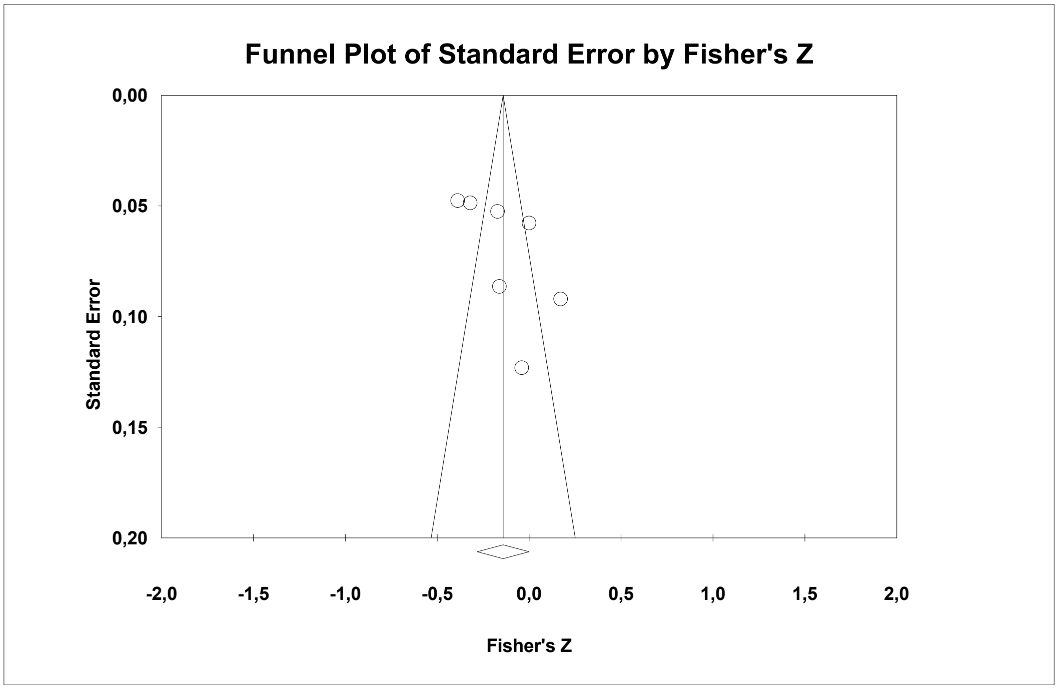

Supplementary Figure 2f. Funnel plot for burnout (others).

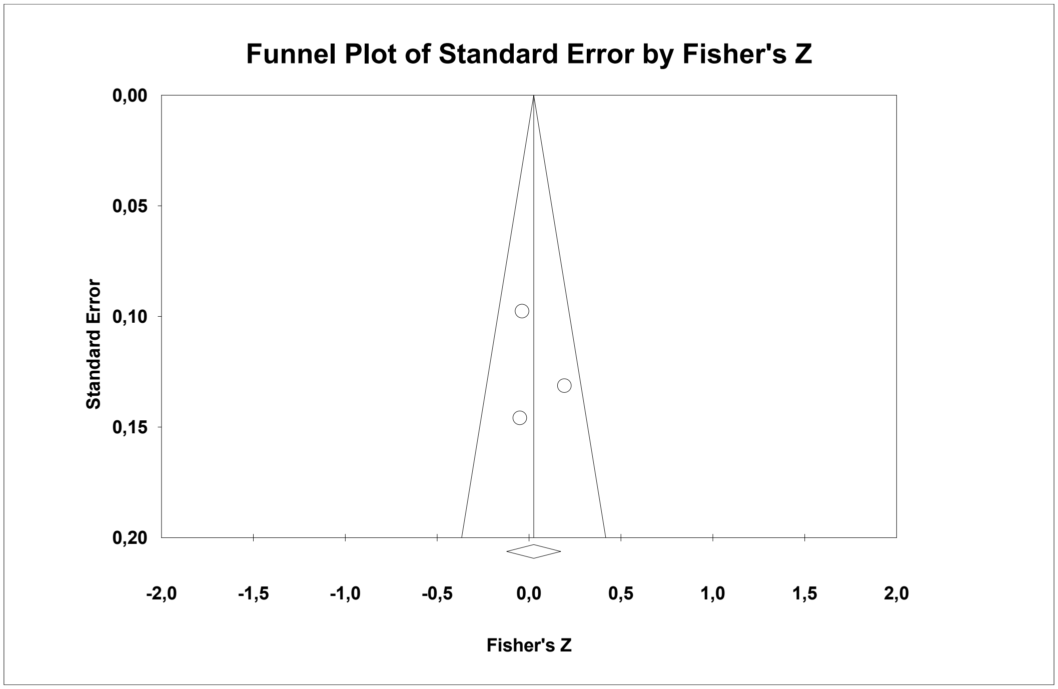

Supplementary Figure 2g. Funnel plot for physiological activation.

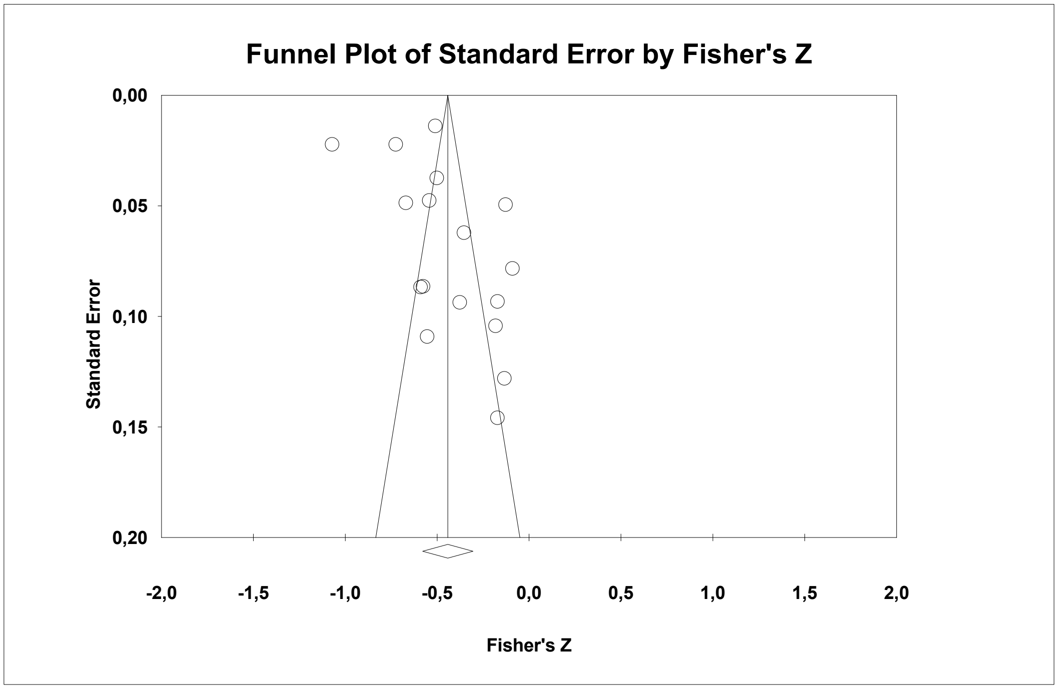

Supplementary Figure 2h. Funnel plot for fatigue.

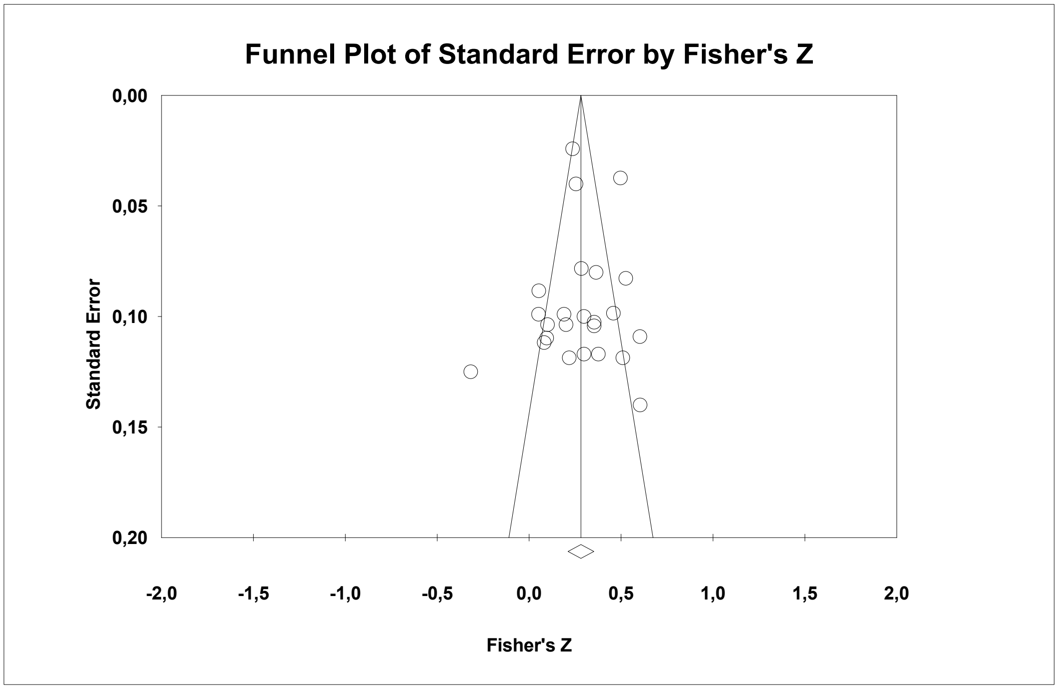

Supplementary Figure 2i. Funnel plot for affect.

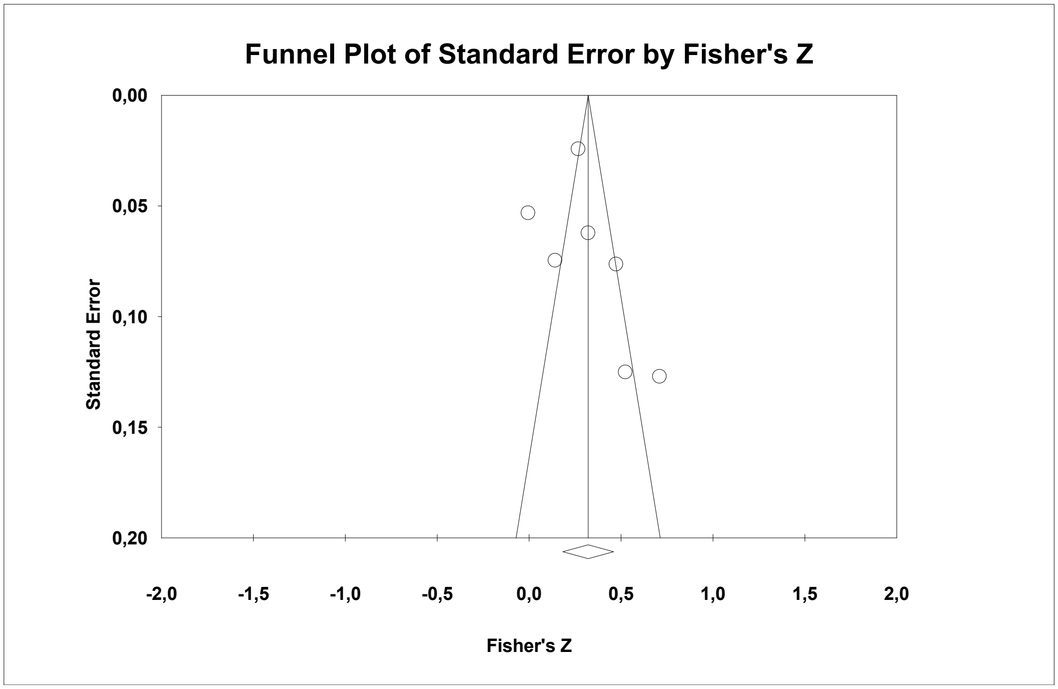

Supplementary Figure 2j. Funnel plot for state of recovery.

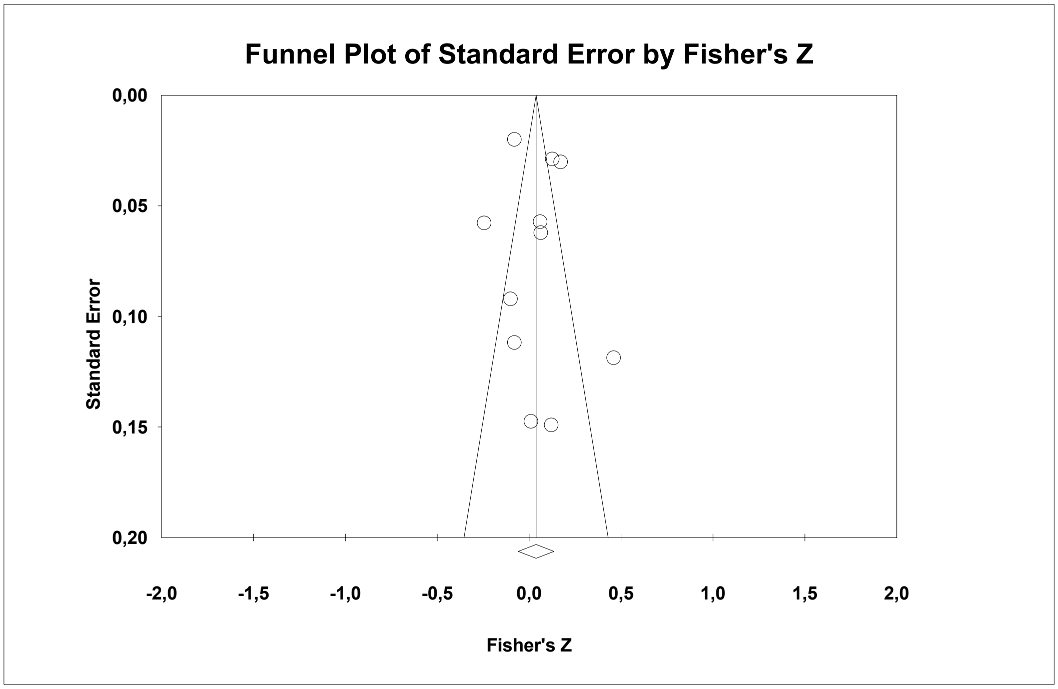

Supplementary Figure 2k. Funnel plot for work motivation.

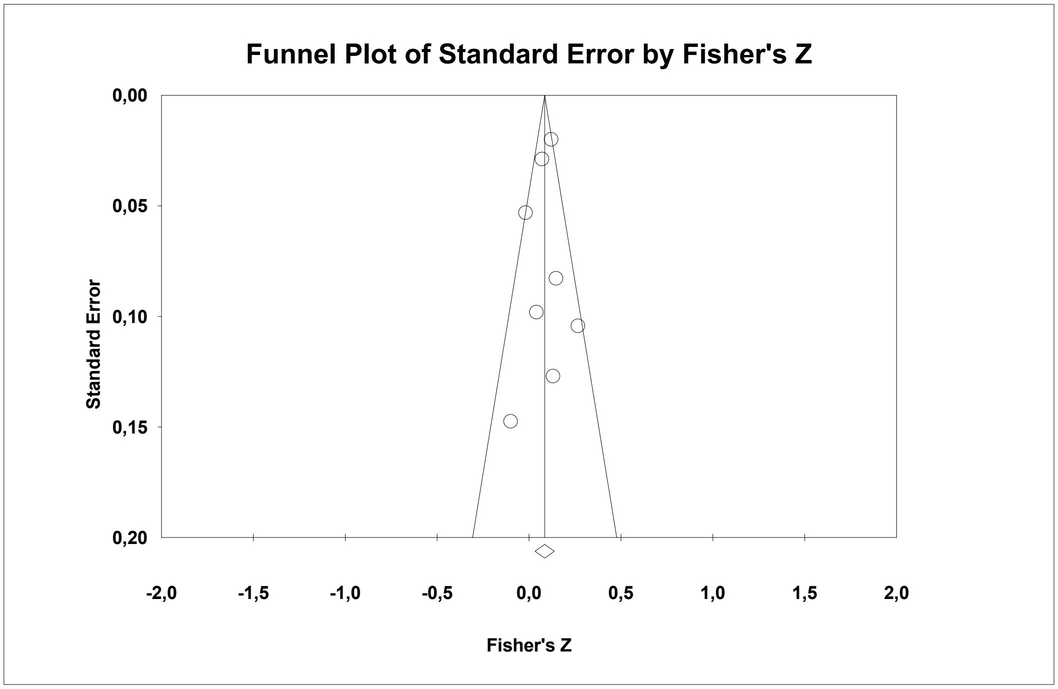

Supplementary Figure 2l. Funnel plot for task performance.

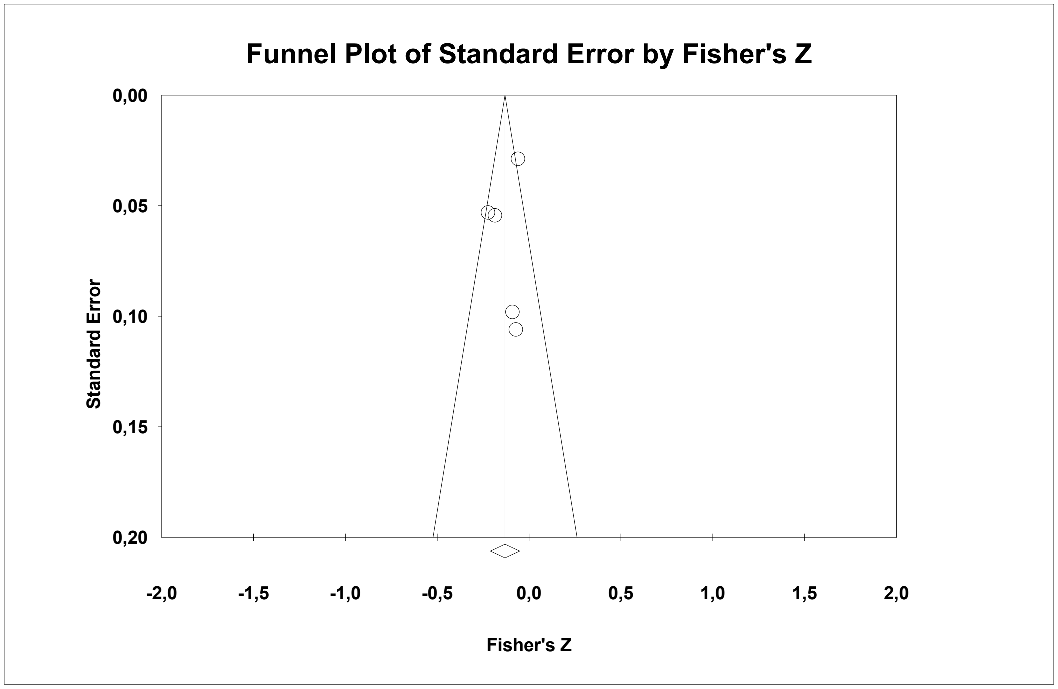

Supplementary Figure 2m. Funnel plot for contextual performance.

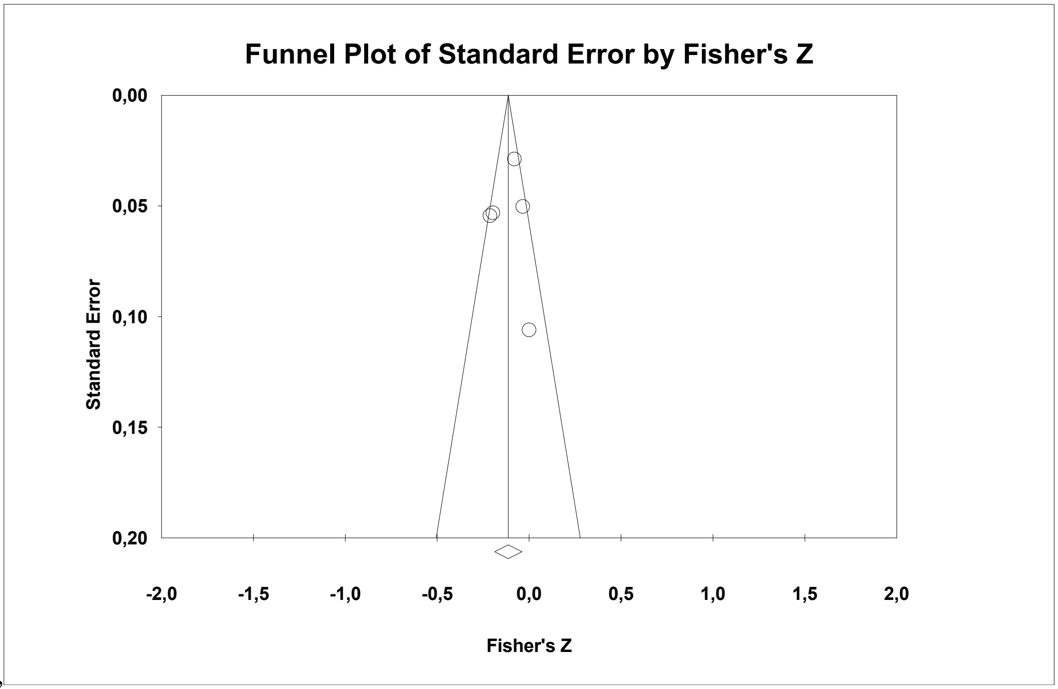

,

Supplementary Figure 2n. Funnel plot for creativity.

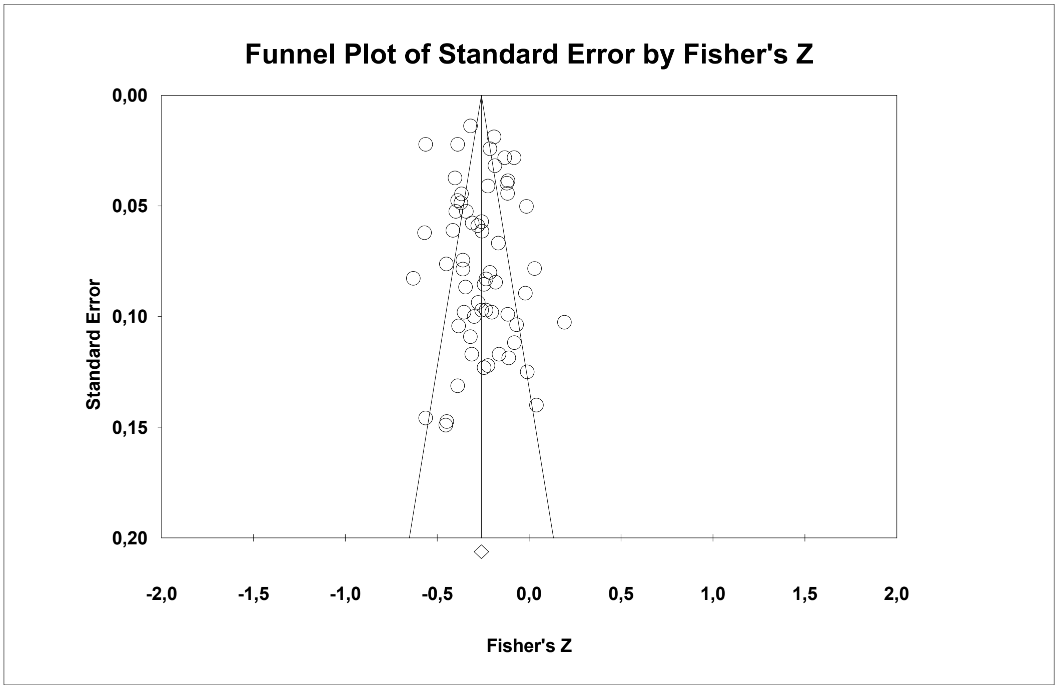

Supplementary Figure 2o. Funnel plot for job demands.

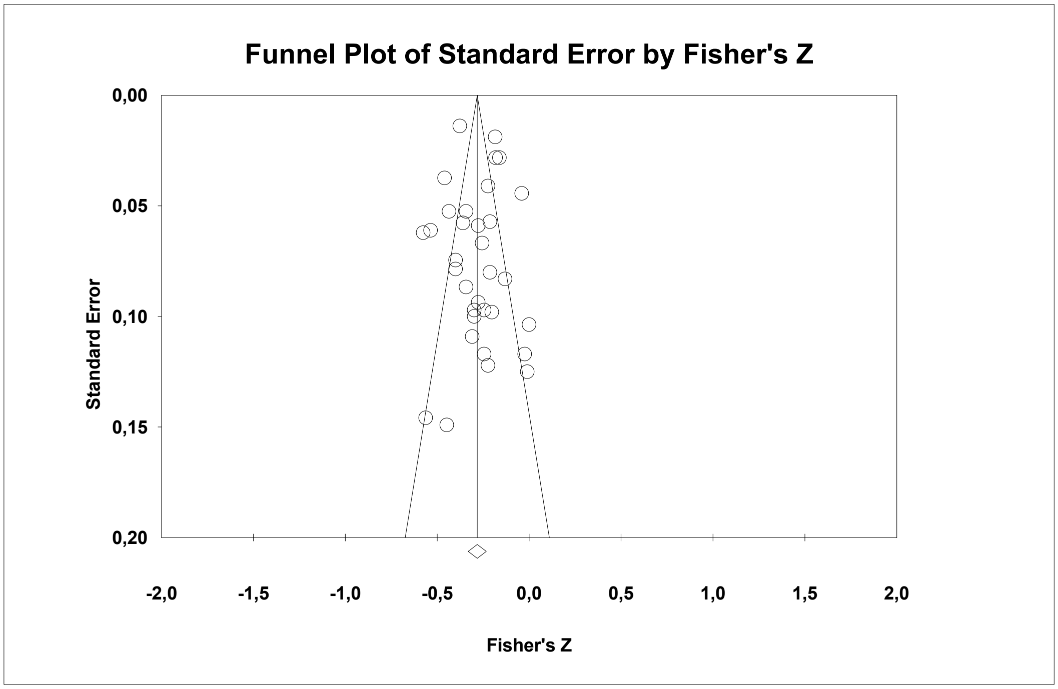

Supplementary Figure 2p. Funnel plot for quantitative demands.

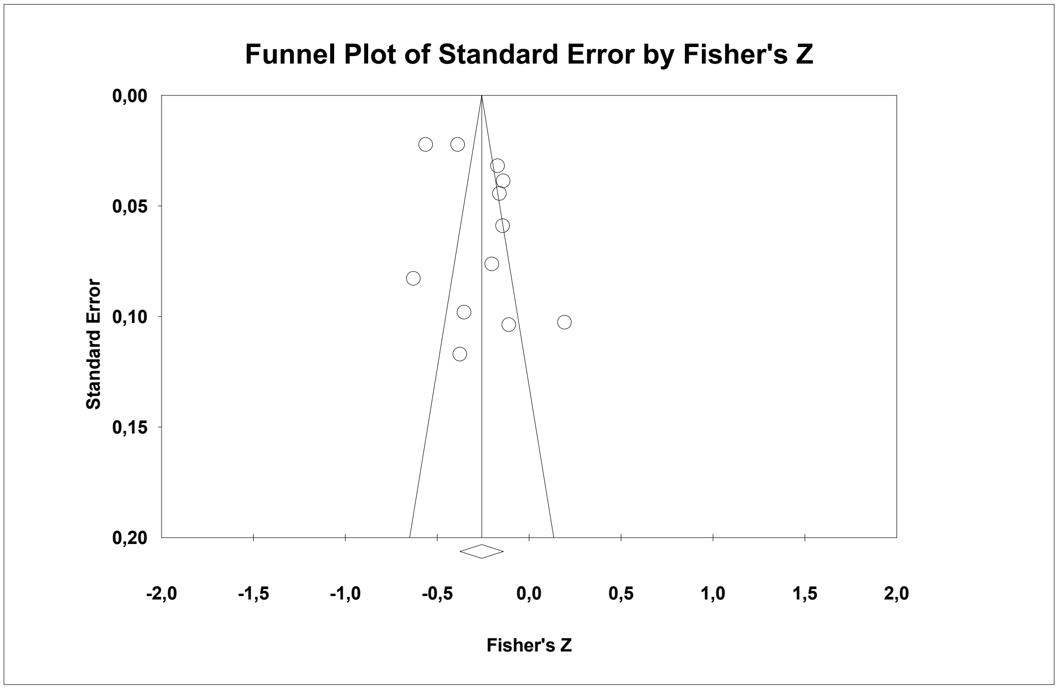

Supplementary Figure 2q. Funnel plot for social conflicts.

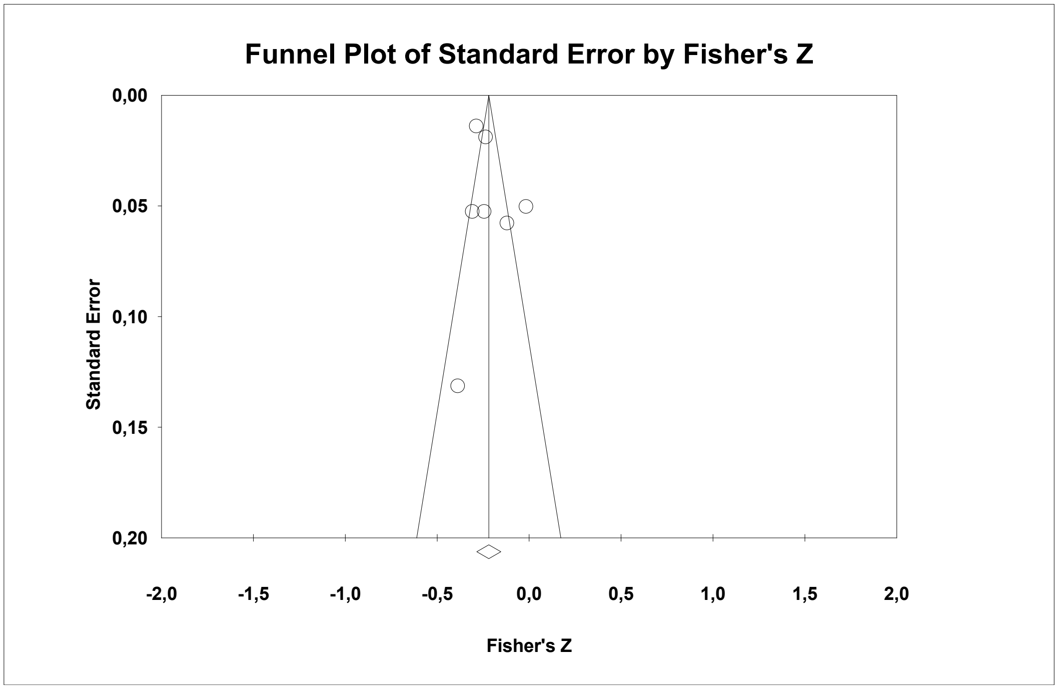

Supplementary Figure 2r. Funnel plot for emotional demands.

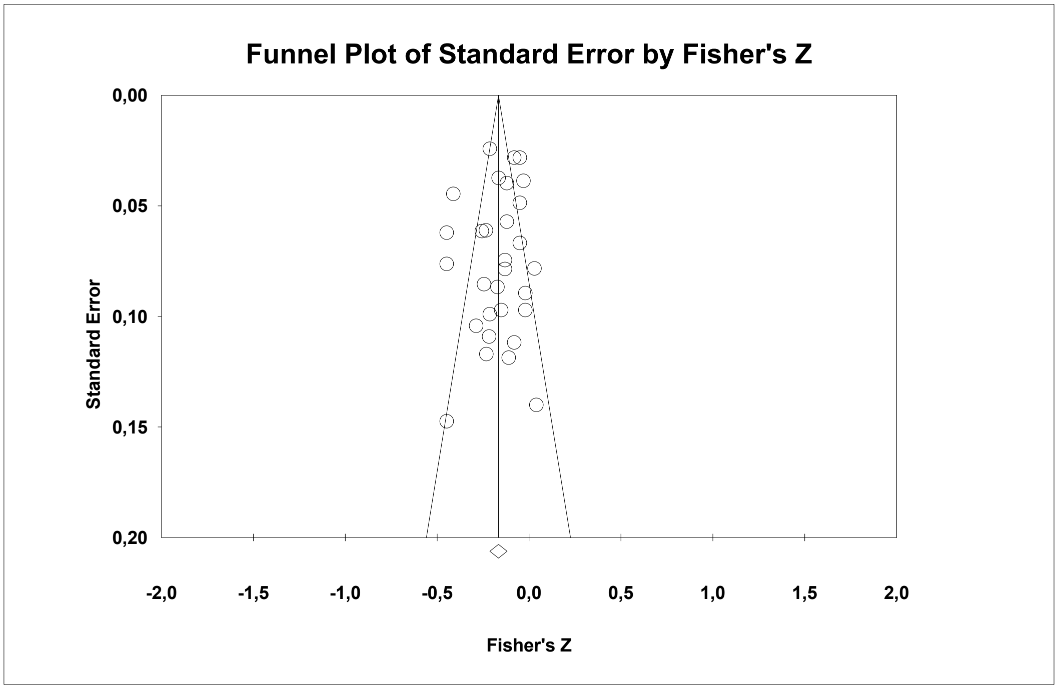

Supplementary Figure 2s. Funnel plot for working time.

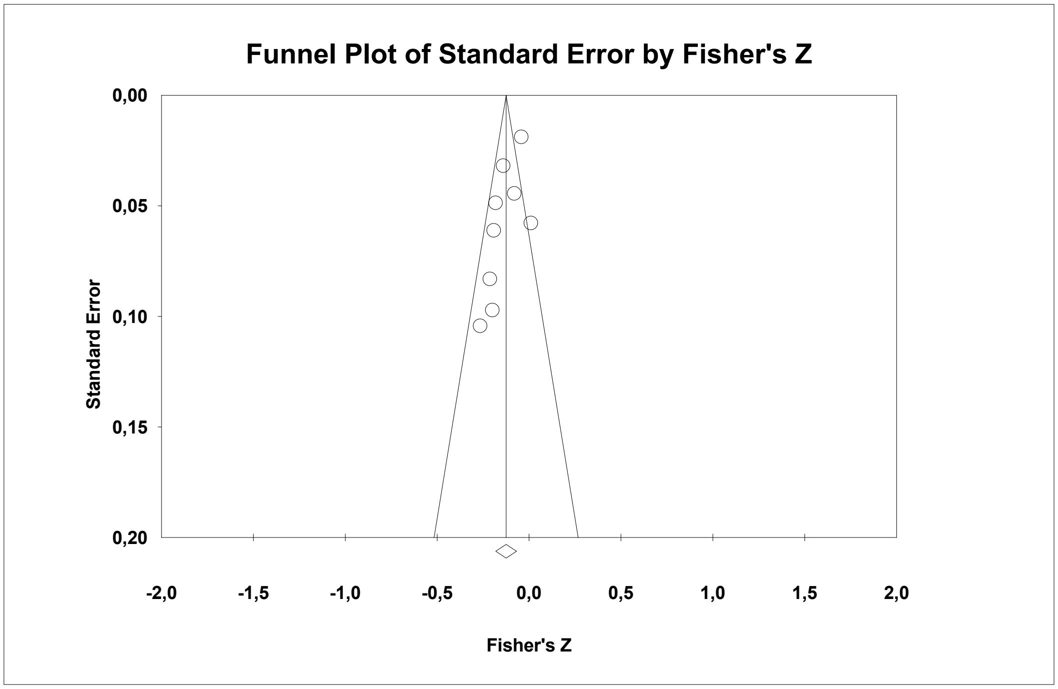

Supplementary Figure 2t. Funnel plot for role stressors.

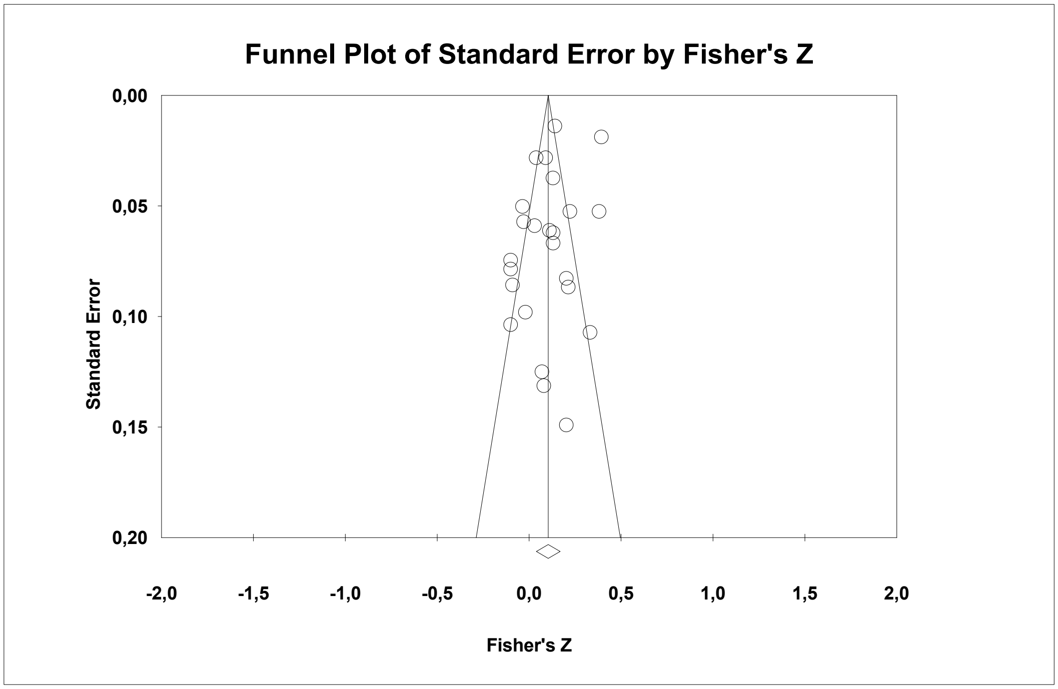

Supplementary Figure 2u. Funnel plot for job resources.

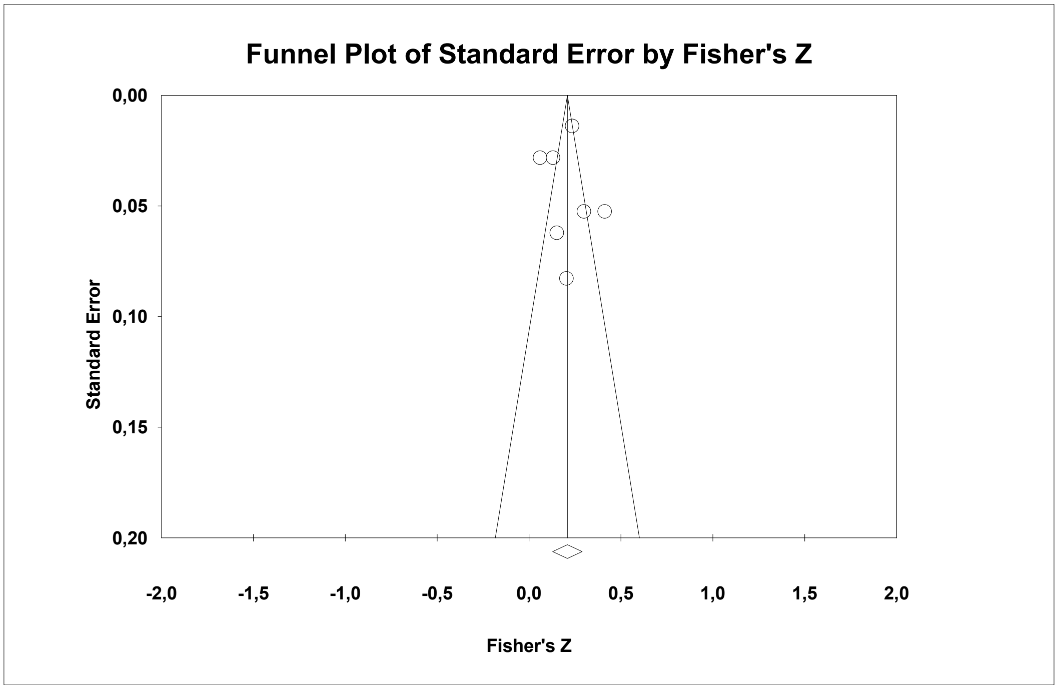

Supplementary Figure 2v. Funnel plot for social support.

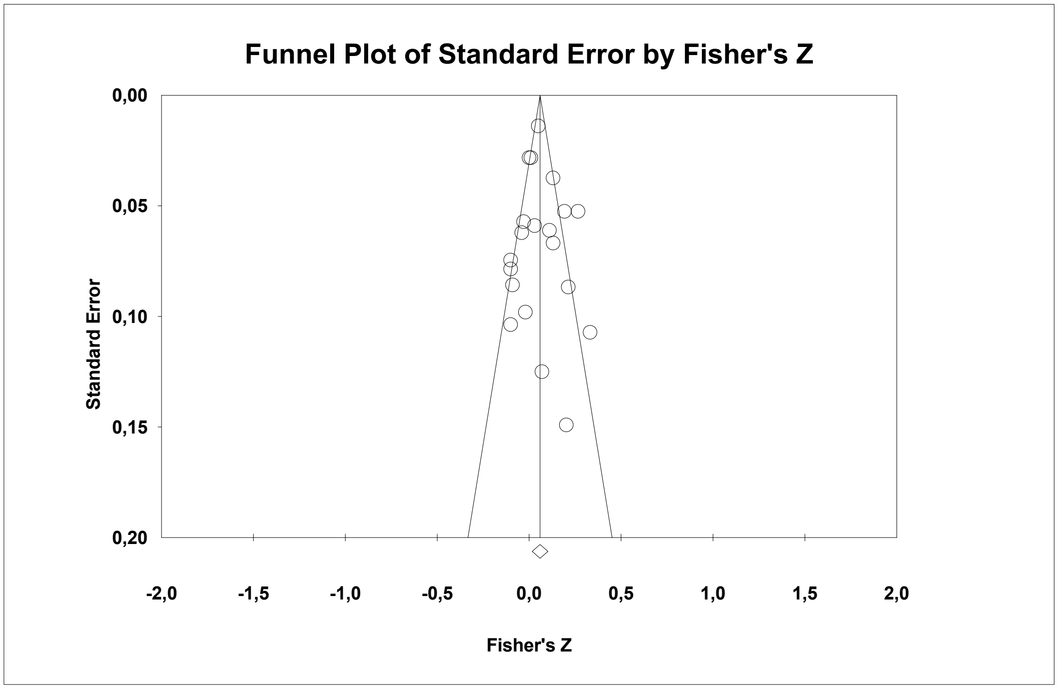

Supplementary Figure 2w. Funnel plot for job control.

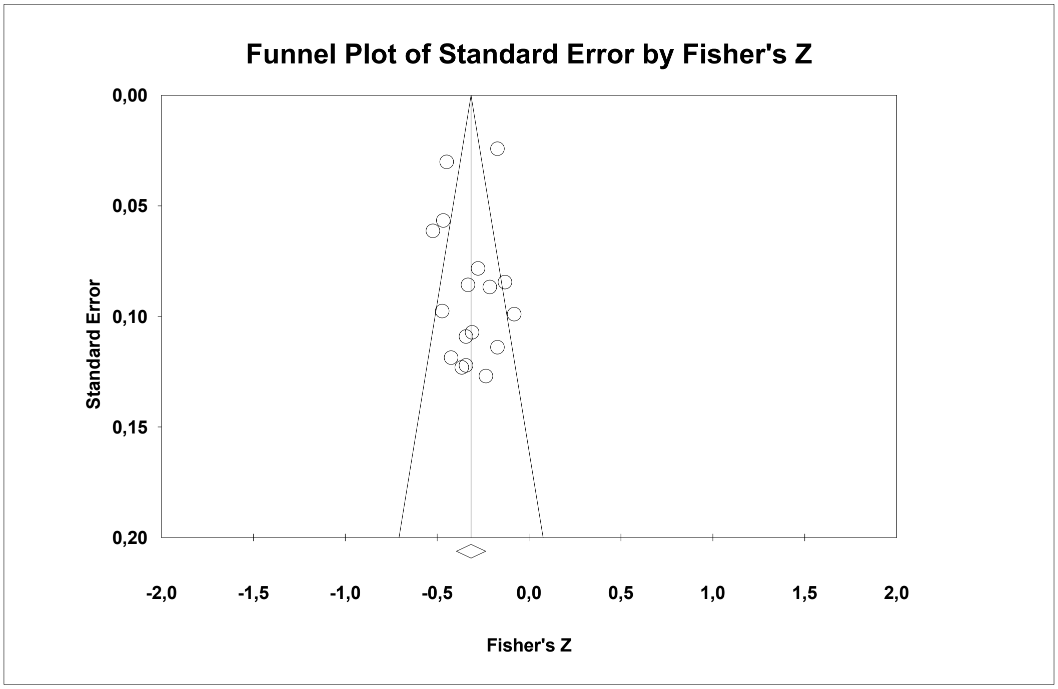

Supplementary Figure 2x. Funnel plot for work-related activities.

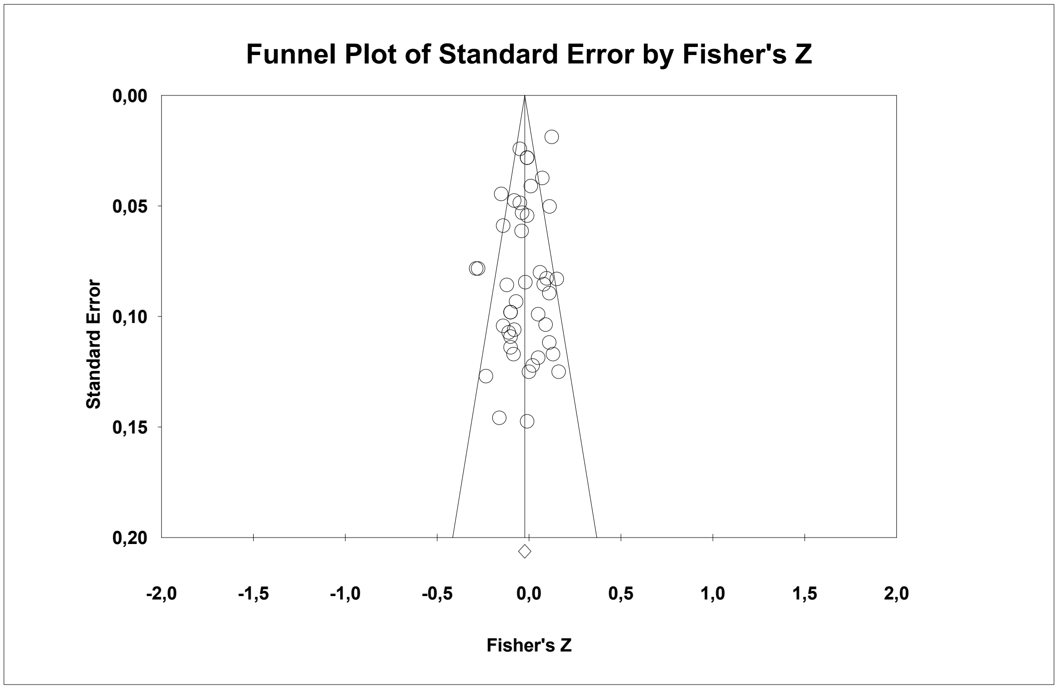

Supplementary Figure 2y. Funnel plot for age.

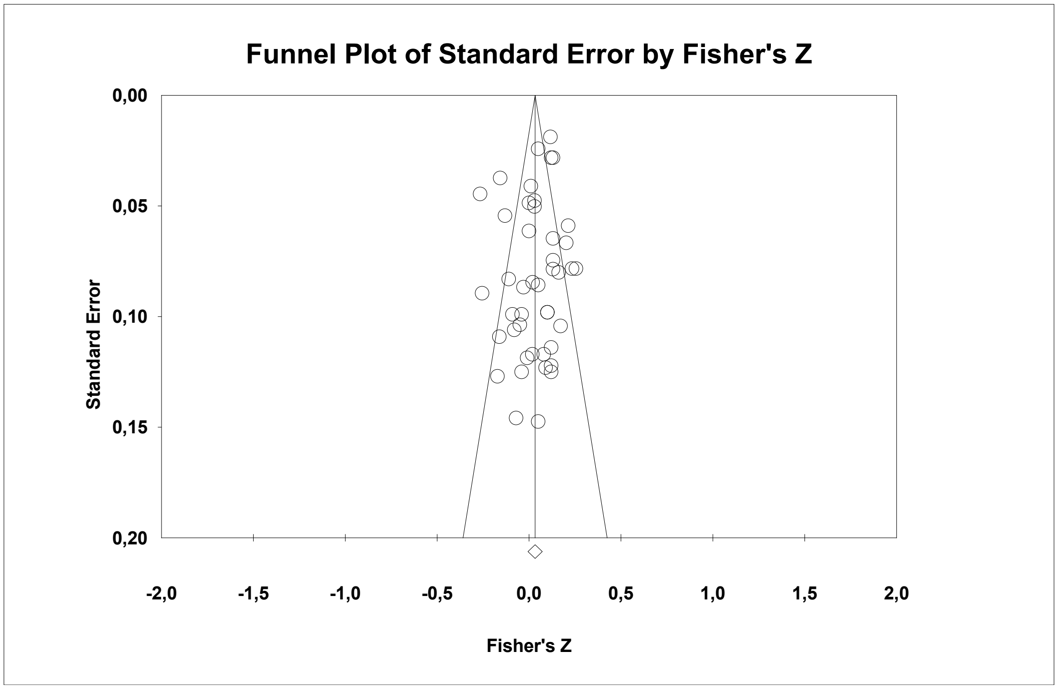

Supplementary Figure 2z. Funnel plot for gender.

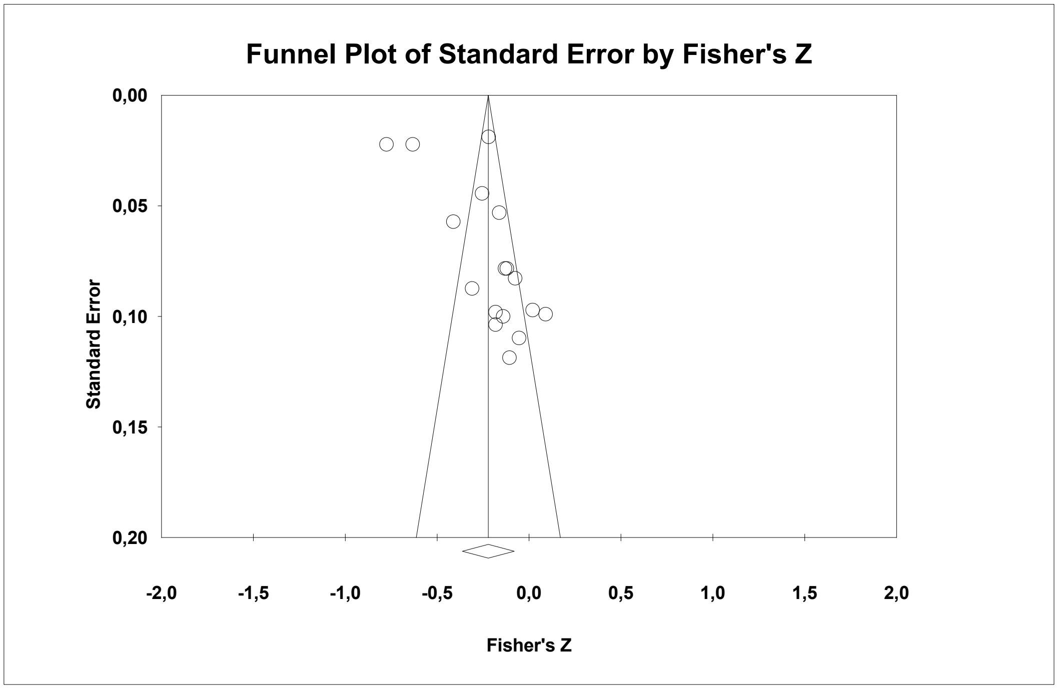

Supplementary Figure 2ab. Funnel plot for negative affectivity/neuroticism.

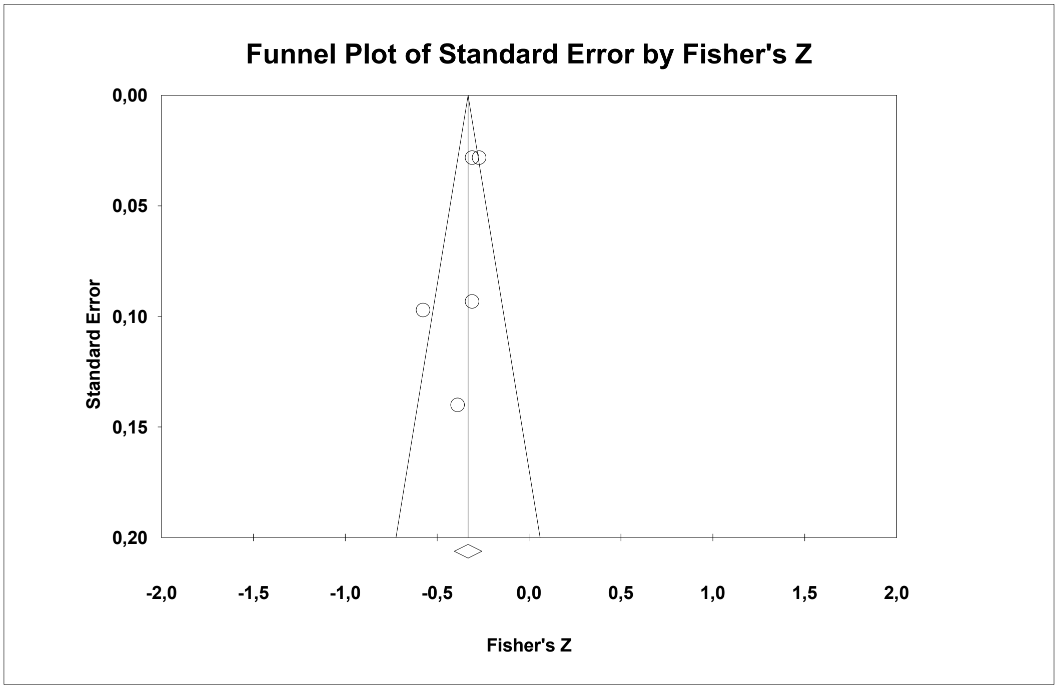

Supplementary Figure 2ac. Funnel plot for heavy work investment.
